# Core fucosylation within the Fc-FcγR degradation pathway promotes enhanced IgG levels *via* exogenous L-fucose

**DOI:** 10.1016/j.jbc.2024.107558

**Published:** 2024-07-11

**Authors:** Yuhan Sun, Xing Xu, Tiangui Wu, Tomohiko Fukuda, Tomoya Isaji, Sayaka Morii, Miyako Nakano, Jianguo Gu

**Affiliations:** 1 Division of Regulatory Glycobiology, Institute of Molecular Biomembrane and Glycobiology, Tohoku Medical and Pharmaceutical University, Sendai, Miyagi, Japan; 2 Graduate School of Integrated Sciences for Life, Hiroshima University, Higashi-Hiroshima, Japan

**Keywords:** immunoglobulins G, Fut8, core fucosylation, L-fucose, Fc receptor

## Abstract

α1,6-Fucosyltransferase (Fut8) is the enzyme responsible for catalyzing core fucosylation. Exogenous L-fucose upregulates fucosylation levels through the GDP-fucose salvage pathway. This study investigated the relationship between core fucosylation and immunoglobulin G (IgG) amounts in serum utilizing WT (*Fut8*^*+/+*^), *Fut8* heterozygous knockout (*Fut8*^*+/−*^), and *Fut8* knockout (*Fut8*^*−/−*^) mice. The IgG levels in serum were lower in *Fut8*^*+/−*^ and *Fut8*^*−/−*^ mice compared with *Fut8*^*+/+*^ mice. Exogenous L-fucose increased IgG levels in *Fut8*^*+/−*^ mice, while the ratios of core fucosylated IgG *versus* total IgG showed no significant difference among *Fut8*^*+/+*^, *Fut8*^*+/−*^, and *Fut8*^*+/−*^ mice treated with L-fucose. These ratios were determined by Western blot, lectin blot, and mass spectrometry analysis. Real-time PCR results demonstrated that mRNA levels of IgG Fc and neonatal Fc receptor, responsible for protecting IgG turnover, were similar among *Fut8*^*+/+*^, *Fut8*^*+/−*^, and *Fut8*^*+/−*^ mice treated with L-fucose. In contrast, the expression levels of Fc-gamma receptor Ⅳ (FcγRⅣ), mainly expressed on macrophages and neutrophils, were increased in *Fut8*^*+/−*^ mice compared to *Fut8*^*+/+*^ mice. The effect was reversed by administrating L-fucose, suggesting that core fucosylation primarily regulates the IgG levels through the Fc-FcγRⅣ degradation pathway. Consistently, IgG internalization and transcytosis were suppressed in *FcγRⅣ*-knockout cells while enhanced in *Fut8*-knockout cells. Furthermore, we assessed the expression levels of specific antibodies against ovalbumin and found they were downregulated in *Fut8*^*+/−*^ mice, with potential recovery observed with L-fucose administration. These findings confirm that core fucosylation plays a vital role in regulating IgG levels in serum, which may provide insights into a novel mechanism in adaptive immune regulation.

Immunoglobulin G (IgG), the primary molecule of the adaptive immune response, is the most abundant immunoglobulin isotype in the plasma. IgG consists of four subclasses in humans (IgG1, IgG2, IgG3, and IgG4) (1) and mice (IgG1, IgG2a, IgG2b, and IgG3) (2), each characterized by distinct structures and functions. The typical structure of IgG is composed of two heavy (H) chains and two light (L) chains linked together by disulfide bonds (3). Each heavy chain comprises a variable domain at the N-terminal and three constant domains (CH1-3). The light chains also possess a variable part at the N-terminal and a constant domain (CL) (3). The ‘‘fragment antigen binding’’ (Fab) domains, known as antigen recognition domains, contain the complementarity determining regions. These regions are located in the N-terminal part of CHs and CLs, responsible for determining the antigen-specificity (4). The “fragment crystallizable’’ (Fc) regions of the C terminal are made up of two CHs (CH2 and CH3), which bind to the immune effector molecules such as Fc receptors (FcγR) (5). Different IgG subclasses have varying affinities for FcγR (6). FcγRs generally exhibit function and structure homology between humans and mice but may also exhibit differences (7). Among IgG subclasses, hIgG1 is the most abundant and dominant subclass of IgG in therapeutics and immune responses (8). Similarly, mIgG2, as orthologs and functional homologs of hIgG1, which shows a preference for Fc-gamma receptor Ⅳ (FcγRⅣ) (9, 10, 11), holds great importance in protective and pathogenic properties in mice, both in innate and adaptive immunity (12).

*N*-glycosylation is the most prevalent modification of IgG. *N*-glycans attached to Fab fragments can influence the antibody’s reactivity. Approximately 25% of Fabs are modified by various glycan types, impacting their structural formation (13, 14), antigen-binding specificity (15, 16), and half-life (17). Notably, the *N*-glycosylation at asparagine 297 (Asn^297^, N^297^) in the Fc fragment is well-known for playing an essential role in the immune response (18, 19, 20). This site has one of the potentially 30 glycan species (21). Among these, core fucosylation, catalyzed by α1,6-fucosyltransferase (Fut8), which transfers L-fucose from GDP-fucose to the innermost GlcNAc, is one of the most pivotal modifications, with more than 94% of IgGs being modified by core fucosylation (22).

The reason why IgGs are highly modified by core fucosylation remains unclear. However, the extent of core fucosylation of IgGs affects the strength of the immune response. A deficiency of core fucosylation on IgG can significantly increase the binding ability between IgG and FcγRIIIα in humans (23) and lead to enhanced complement-dependent cytotoxicity, antibody-dependent cellular cytotoxicity (ADCC) (24, 25). On the other hand, the lower levels of core fucosylated IgG are associated with the severity of some diseases. For instance, in the fetuses mediating fetal or neonatal alloimmune thrombocytopenia, the alloantibodies IgG1 against human platelet antigens during pregnancy contain lower core fucosylation, correlating with disease severity (26). Similarly, recent studies have also shown that afucosylated (noncore fucosylated) IgG1 is closely associated with disease severity in dengue fever (18) and COVID-19 (27). Very recently, we found that the level of afucosylated IgG was increased in the sera of the patients with lung cancer, chronic obstructive pulmonary disease, and interstitial pneumonia compared to healthy subjects (28). Therefore, the degree of core fucosylation of IgG modulates its binding to the Fc receptors, with significant implications for the efficacy of antibody-based therapies, vaccine development, and immune therapy.

In the current study, we investigated the significance of core fucosylation in regulating IgG levels and the underlying mechanisms for altering IgG in *Fut8* heterozygous knockout (*Fut8*^*+/−*^) mice. We observed a reduction in IgG levels in *Fut8*^*+/−*^ mice compared to the WT (*Fut8*^*+/+*^) mice, which could be restored by the administration of L-fucose, thereby enhancing GDP-fucose levels through the salvage pathway (29). This phenomenon was further confirmed by producing specific antibodies against ovalbumin (OVA). Additionally, we found that FcγRⅣ, a mouse IgG receptor that mediates IgG binding, leading to endocytosis and degradation, was highly expressed in *Fut8*^*+/−*^ mice. Interestingly, exogenous L-fucose reduced the expression of FcγRⅣ, resulting in increased IgG levels in *Fut8*^*+/−*^ mice. These findings introduce a novel concept of regulating IgG stability and identify L-fucose as an effective agent for immune therapy.

## Results

### Exogenous L-fucose increased IgG levels and core fucosylation in the spleens of *Fut8*^*+/−*^ mice

**Figure 1:**
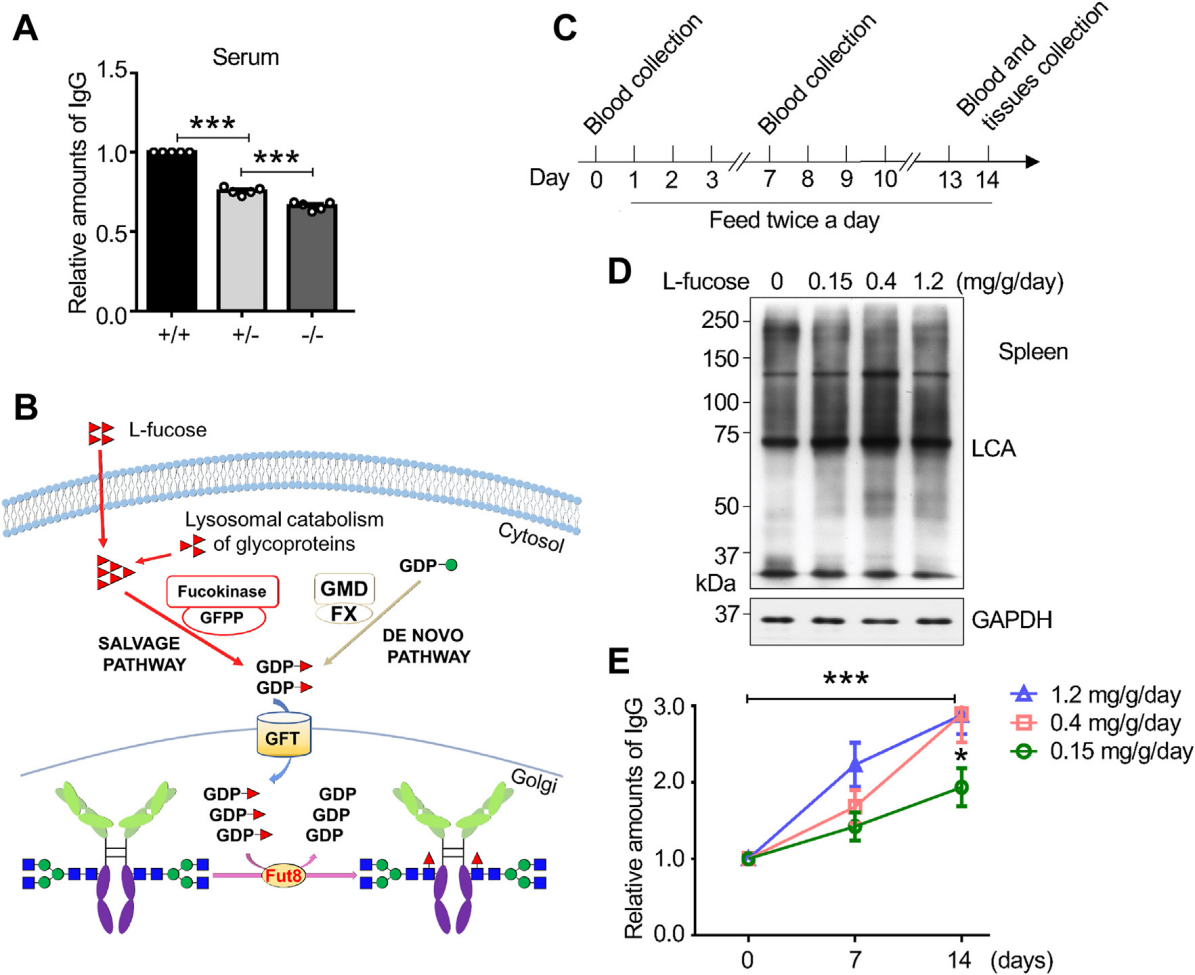
**Effects of exogenous L-fucose on IgG levels and core fucosylation in the spleens of *Fut8***^***+/−***^**mice**. *A*, comparison of IgG levels among the *Fut8*^*+/+*^, *Fut8*^*+/−*^, and *Fut8*^*−/−*^ mice. IgG was purified by immunoprecipitated in serum as described in “Experimental procedures”. Equal serum (5 μl) was immunoprecipitated by Ab Capcher and detected by anti-mouse IgG antibody. The IgG level of the *Fut8*^*+/+*^ group was set as 1.0 and analyzed by one-way ANOVA with Tukey’s *post hoc* analysis by GraphPad Prism version 6 as the mean ± SEM. ∗∗∗*p* < 0.001. The data were obtained from five mice. *B*, there are two pathways for producing GDP-fucose in cells: *de novo* and salvage. The exogenous L-fucose can be metabolized to the GDP-fucose *via* the salvage pathway and provide more substrate for the biosynthesis of core fucosylation. *C*, schedule of L-fucose administration with the concentration at 0.15, 0.4, or 1.2 mg/g/day, twice daily, lasting 14 days. Blood was collected on the 0, 7th, and 14th days. *D*, effects of exogenous L-fucose on core fucosylation in spleen tissues. After the pretreatment described in (*C*), the same amounts of spleen tissues were extracted and detected using LCA lectin blot. GAPDH was used as a loading control. *E*, effects of exogenous L-fucose on IgG levels. The data were obtained from at least three mice and analyzed by one-way ANOVA with Tukey’s *post hoc* analysis as the mean ± SEM. The relative levels of IgGs at 0 days were set as 1.0. ∗*p* < 0.05 ∗∗∗*p* < 0.001. Fut8+/−, Fut8 heterozygous knockout; Fut8^*−/−*^, *Fut8* knockout; IgG, immunoglobulin G; LCA, *Lens culinaris* agglutinin.

Fut8 is the exclusive fucosyltransferase responsible for catalyzing core fucosylation (30). Several studies have explored modifying the fucosylation of therapeutic antibodies to enhance their ADCC activity (24, 25), particularly in the development of monoclonal antibody-based cancer treatments, where ADCC plays a pivotal role in eliminating cancer cells (31). To investigate the effect of core fucosylation on IgG, we compared the expression levels of IgG extracted from sera in *Fut8*^*+/+*^, *Fut8*^*+/−*^*,* and *Fut8* homozygous knockout (*Fut8*^*−/−*^) mice. A significant reduction in IgG level was observed in *Fut8*^*+/−*^ and *Fut8*^*−/−*^ mice compared with *Fut8*^*+/+*^ mice (Fig. 1*A*). It also showed that the IgG level in *Fut8*^*−/−*^ mice was significantly lower than that in the *Fut8*^*+/−*^ mice. However, we encountered difficulties obtaining sufficient viable *Fut8*^*−/−*^ mice and bone marrow samples for the following studies. Given these constraints, we opted to utilize *Fut8*^*+/−*^ mice, which are more viable and allowed us to conduct the present study using exogenous L-fucose. Considering the decreased core fucosylation may be related to the phenomenon, we administered exogenous L-fucose to *Fut8*^*+/−*^ mice. This treatment can increase the donor substrate GDP-fucose through the salvage pathway, leading to upregulation of core fucosylation (29, 32) (Fig. 1*B*). Mice were treated with different concentrations of L-fucose. Their sera were collected on the 0, 7th, and 14th day, as shown in Figure 1*C*. On the 14th day, we isolated the spleen tissues where B cells underwent further development and activation. The levels of core fucosylation in *Fut8*^*+/−*^ mice spleens significantly increased after treatment with L-fucose, as evidenced by *Lens culinaris* agglutinin (LCA) lectin blot (Fig. 1*D*). Interestingly, the concentration of L-fucose at 0.4 mg/g/day was the most effective. The amounts of IgG were significantly increased in *Fut8*^*+/−*^ mice after treatment with L-fucose, compared to the group without treatment (Fig. 1*E*).

### Exogenous L-fucose did not affect the ratio of core fucosylated IgG *versus* total IgG

**Figure 2:**
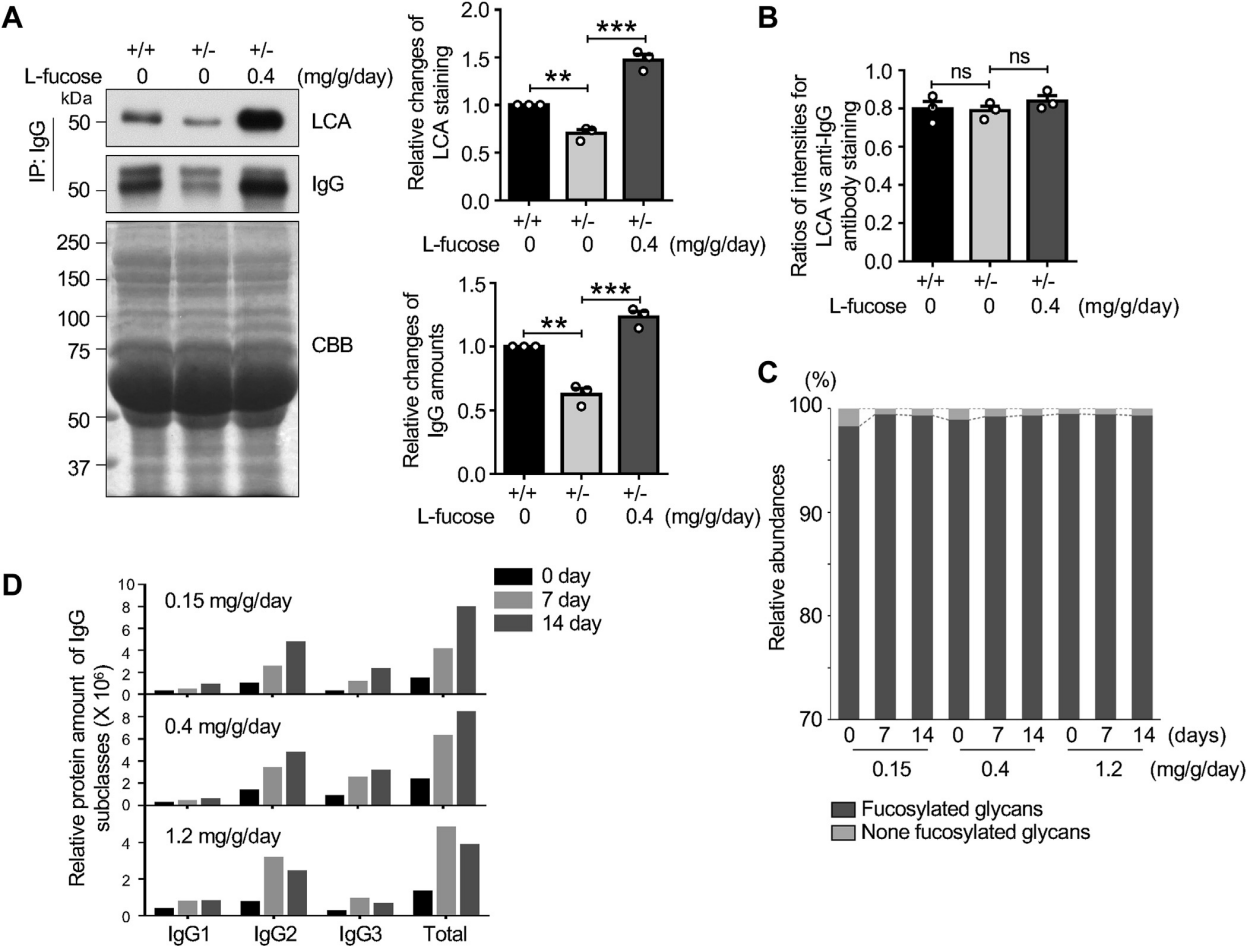
**Effects of exogenous L-fucose on core fucosylated IgG levels**. *A*, equal amount of serum (2 μl) was pulled down by Ab Capcher. IgG levels were evaluated using Western blotting with anti-mouse IgG antibody, while the core fucosylated IgG levels were detected using LCA lectin blot. CBB was used as a loading control. The core fucosylated IgG level of the Fut8^+/+^ group was set as 1.0. The data were repeated from three mice and analyzed by Image J using one-way ANOVA with Tukey’s *post hoc* analysis as the mean ± SEM. ∗∗*p* < 0.01; ∗∗∗*p* < 0.001. *B*, the ratios of core fucosylated IgG to total IgG were analyzed using the data in (*A*). ns, no significance, *p* > 0.05. *C*, comparison of core fucosylated IgG levels in *Fut8*^*+/−*^ with or without L-fucose based on results of liquid chromatography electrospray ionisation tandem mass spectrometry analysis. The detailed results and data are shown in Tables S1–S10 and Figs. S1–S16. Data were obtained from a mixture of three mice. *D*, a comparison of IgG subclass levels in *Fut8*^*+/−*^ with or without L-fucose based on intensities of a peptide of IgG1 (DDPEVQFSWFVDDVEVHTAQTQPR, [M+3H]^3+^ = 949.108 ± 6 ppm), IgG2 (APQVYILPPPAEQLSR, [M+2H]^2+^ = 889.995 ± 6 ppm) and IgG3 (NTPPILDSDGTYFLYSK, [M+2H]^2+^ = 965.977 ± 6 ppm) by liquid chromatography electrospray ionisation tandem mass spectrometry analysis. The data were obtained from a mixture of three mice. The data on intensities are shown in Table S11. CBB, Coomassie brilliant blue; Fut8+/−, Fut8 heterozygous knockout; IgG, immunoglobulin G; LCA, *Lens culinaris* agglutinin; MS, mass spectrometry.

To investigate whether exogenous L-fucose impacted the core fucosylation levels of IgG, we assessed total IgG using Western blot and the core fucosylated IgG *via* LCA lectin blot. Consistent with the findings in Figure 1*E*, both total IgG and core fucosylated IgG amounts were decreased in *Fut8*^*+/−*^ compared to *Fut8*^*+/+*^ and *Fut8*^*+/−*^ mice treated with L-fucose (Fig. 2*A*). However, the ratio of core fucosylated IgG to total IgG showed no significant differences (Fig. 2*B*). Furthermore, we investigated *N*-glycan structures of IgG through mass spectrometry (MS). The detailed data information is in Tables S1–S11 and Figs. S1–S16. We observed no significant differences between the treatments with or without L-fucose in *Fut8*^*+/−*^ mice (Fig. 2*C* and Table S10). The levels of core fucosylated *N*-glycans accounted for more than 98% of all *N*-glycans, even without the treatment. In line with these results, each subclass of IgG showed an increase after L-fucose treatment, with IgG2 being the predominant IgG subclass (Fig. 2*D* and Table S11). These results indicate that a deficiency of Fut8 reduces IgG levels, while exogenous L-fucose can restore both core fucosylation and IgG levels. These suggest that core fucosylation plays an essential role in IgG expression.

It is worth noting that IgG2 is the majority subclass in *Fut8*^*+/−*^ and *Fut8*^*+/+*^ mice (Table S11). However, the expression ratios of IgG1 and IgG3 seem to differ between both mice (Table S11). The underlying mechanisms remain under further study. We also noticed that the expression levels of Igκ (TSTSPIVK, m/z 832.478 shown in Fig. S2) beside IgG were suppressed in *Fut8*^*+/−*^ mice, which were rescued by exogenous L-fucose (Table S11).

### Comparison of the effects of exogenous L-fucose on mRNA levels of IgG

**Figure 3:**
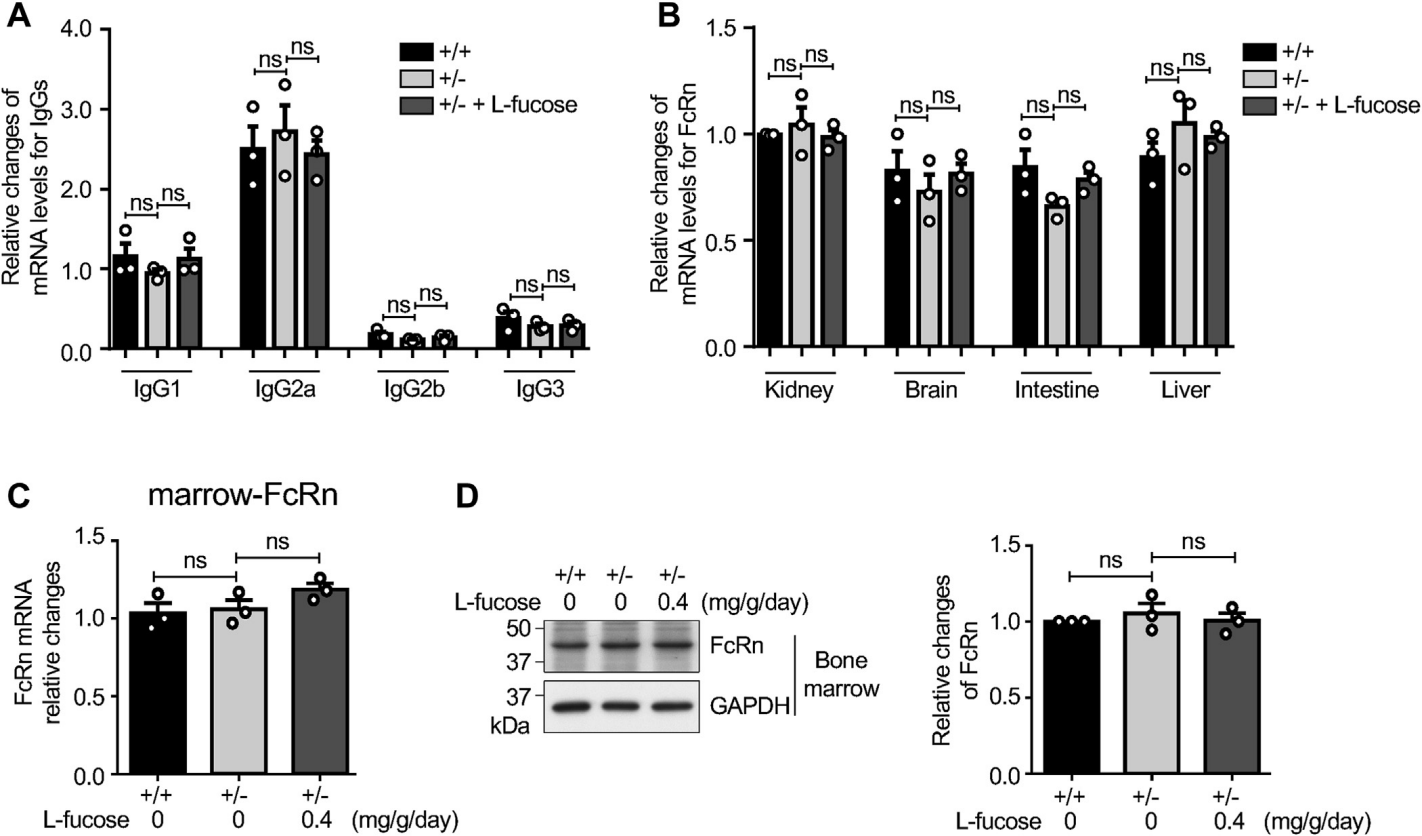
**Comparison of the expression levels of IgG and FcRn in *Fut8***^***+/+***^**mice and *Fut8***^***+/−***^**mice treated with or without L-fucose**. *A*, RNAs were extracted from the same amounts of spleen tissue (50 mg) after the pretreatment described above, and then the mRNA levels for the common Fc region of IgG were examined using qPCR. Comparison of the mRNA levels of IgG among *Fut8*^*+/+*^, *Fut8*^*+/−*^, and *Fut8*^*+/−*^ treated with 0.4 mg/g/day L-fucose mice. The data were obtained from three mice, and GAPDH was used as an internal control. The data of IgG1 mRNA levels in *Fut8*^*+/+*^ mice were set as 1.0. *B*, RNAs were extracted from the same amounts of kidney, brain, intestine, and liver tissues. The mRNA levels of FcRn were examined using qPCR. Comparison of the mRNA levels of FcRn in various tissues among *Fut8*^*+/+*^ mice *Fut8*^*+/−*^, mice and *Fut8*^*+/−*^ treated with L-fucose mice. The data were obtained from three mice, and the data of FcRn mRNA levels in the kidney of *Fut8*^*+/+*^ mice were set as 1.0. *C*, the mRNA levels of FcRn in marrow were examined using qPCR. The data were obtained from three mice, and the data of *Fut8*^*+/+*^ mice were set as 1.0. *D*, the expression levels of FcRn protein in bone marrow were detected using Western blotting, and GAPDH was used as a loading control. The data were obtained from three mice, and the data of *Fut8*^*+/+*^ mice were set as 1.0. The data were analyzed by one-way ANOVA with Tukey’s *post hoc* analysis as the mean ± SEM. ns, no significance, *p* > 0.05. Fut8+/−, Fut8 heterozygous knockout; IgG, immunoglobulin G; qPCR, quantitative PCR.

Since the total amount of IgG were altered without significant changes in the ratio of core fucosylated IgG/total IgG, we examined the mRNA levels of IgG subclasses. As detailed in Table 1, we designed primers for each Fc fragment of different IgG subclasses, including IgG1, IgG2a, IgG2b, and IgG3, and performed real-time PCR. As shown in Figure 3*A*, there were no significant differences between *Fut8*^*+/+*^ and *Fut8*^*+/−*^ mice treated with or without L-fucose regarding IgG mRNA levels. Notably, the administration of exogenous L-fucose also had no significant impact on the mRNA levels for IgG in the *Fut8*^*+/−*^ mice. These results suggest that the reduced IgG levels in the *Fut8*^*+/−*^ mice were not related to the transcriptional changes in IgG.

**Table 1:** Primer sequences for real-time PCR

Target genes	Primer sequences (5′-3′)	Reverse sequences
	Forward sequences	
IgG1-Fc	CTCCACAGGTGTACACCATT	CAGGCCCTCATGTAACACAG
IgG2a-Fc	AAGGGCTAGTCAGAGCTCCA	GGTGCGGTGTCCTTGTAGTT
IgG2b-Fc	TCATGCAACGTGAGACACGA	CTTCAGCTCCACCACTGAGG
IgG3-Fc	GCTTGGTGACTGTACCCTCC	CATCTGGGTCATCCTCGCTC
FcRn	GGCCTGAGACGGAAATCGTT	ATTGCGCAGGAATCGGAACT
FcγRI	CTTCTACGTGGGCAGCAAGA	CACAGTCACCCACTGAGCTT
FcγRIIB	AGGTGCTCAAGGAAGACACG	CGTGATGGTTTCCCCTTCCA
FcγRIII	ATGGTGACACTGATGTGCGA	CGTGATGGTTTCCCCTTCCA
FcγRIV	TTGAGGAAGACAGCGTGACC	GTCCTGAGGTTCCTTGCTCC
GAPDH	ACTCCACTCACGGCAAATTC	CCCTGTTGCTGTAGCCGTAT

### Core fucosylation regulated the expression levels of FcγRⅣ, not FcRn

FcRn plays a crucial role in extending the half-life of IgG in circulation by binding to the Fc portion of IgG, thereby reducing IgG degradation. The FcRn achieves this by binding to IgG in acidic conditions within endosomes, recycling it back to the cell surface, and releasing it at physiological pH (33). FcRn is mainly expressed in the brain, lungs, kidneys, and intestine (34, 35). To investigate whether the changes in IgG levels were related to its stabilization mechanism, we examined the expression levels of the FcRn gene in various tissues of *Fut8*^*+/+*^ and *Fut8*^*+/−*^ mice treated with or without L-fucose using real-time PCR. There were no significant differences in FcRn gene expression levels between *Fut8*^*+/+*^ and *Fut8*^*+/−*^ mice with or without exogenous L-fucose (Fig. 3, *B* and *C*). The Western blot results also showed the expression levels of FcRn have no differences among *Fut8*^*+/+*^, *Fut8*^*+/−*^*,* and *Fut8*^*+/−*^ with exogenous L-fucose mice (Fig. 3*D*), suggesting the core fucose may not affect the FcRn expression and trafficking. In addition, it has been reported that the fucosylation on IgG Fc regions does not seem to significantly alter its interaction with FcRn and affect its serum half-life mediated by FcRn (36). These results suggest that the decreased IgG levels in the *Fut8*^*+/−*^ mice were not due to alterations in its stabilization mechanism.

**Figure 4:**
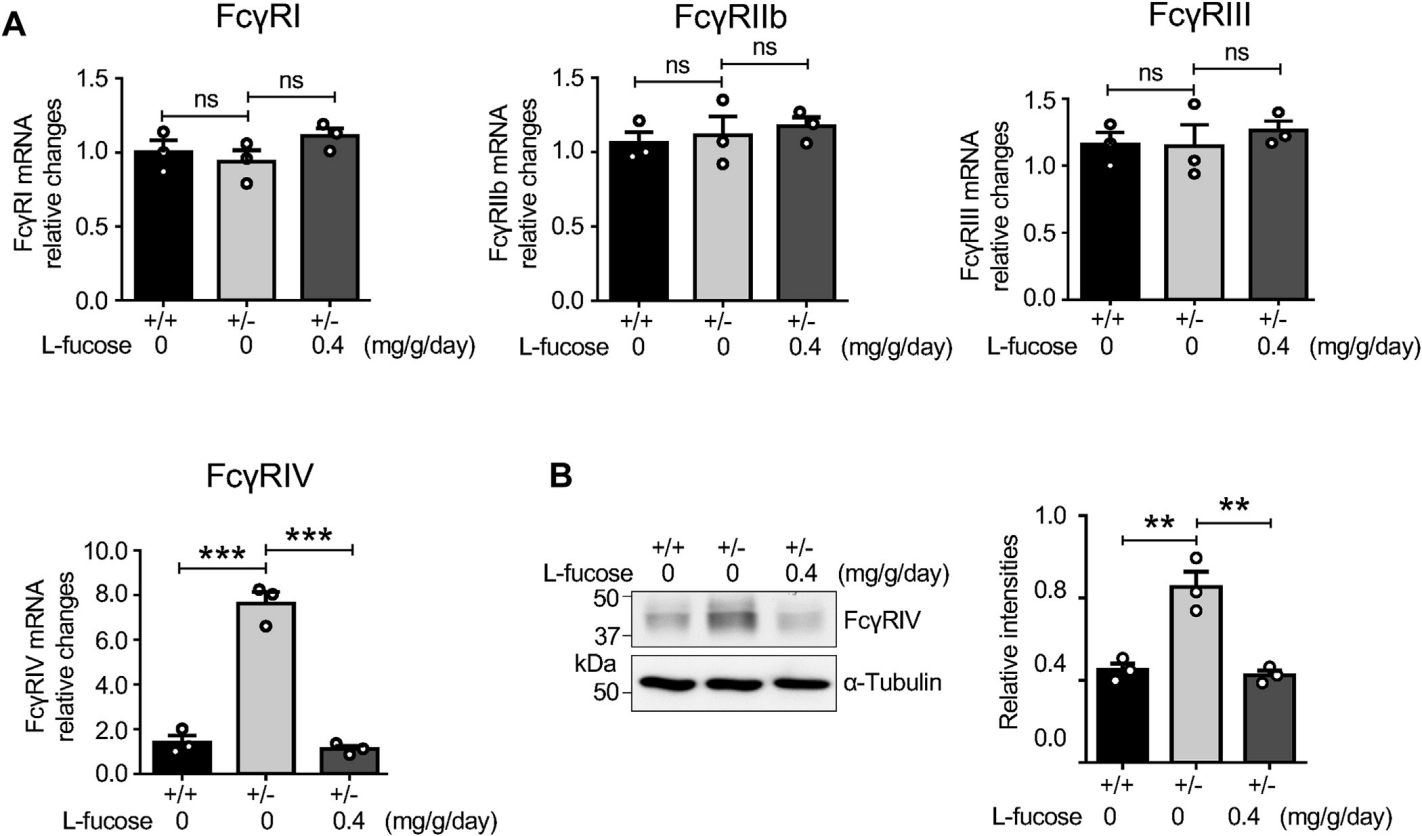
**Effect of exogenous L-fucose on the expression levels of FcγRs**. *A*, RNAs were extracted from bone marrow after the pretreatment as described above. The mRNA levels of FcγRs, including FcγRI, FcγRII, FcγRIII, and FcγRIV, were examined in *Fut8*^*+/+*^, *Fut8*^*+/−*^, and *Fut8*^*+/−*^ mice treated with L-fucose at 0.4 mg/g/day. GAPDH was used as an internal control. Data were repeated in three mice, and the data of *Fut8*^*+/+*^ mice were set as 1.0, which was analyzed by one-way ANOVA with Tukey’s *post hoc* analysis as the mean ± SEM. ns, no significance, *p* > 0.05. ∗∗∗*p* < 0.001. *B*, the expression levels of FcγRIV protein in marrow tissues were detected using Western blot. α-Tubulin was used as a loading control. Data were repeated in three mice and qualified by one-way ANOVA with Tukey’s *post hoc* analysis as the mean ± SEM. ∗∗*p* < 0.01. Fut8+/−, Fut8 heterozygous knockout; FcγRⅣ, Fc-gamma receptor Ⅳ.

Subsequently, we focused on its degradation pathway. FcγRs are cell surface receptors that bind to the Fc region of IgG, allowing the immune cells to recognize and respond to targets marked for destruction by IgG (37) and primarily expressed on all myeloid (38). Therefore, the expression levels of FcγRs could regulate IgG expression levels in the serum. We isolated the bone marrow from *Fut8*^*+/+*^ and *Fut8*^*+/−*^ mice and detected the mRNA levels of FcγRs using quantitative PCR. Notably, the expression levels of the FcγRⅣ gene, but not others, were significantly increased in *Fut8*^*+/−*^ mice compared to the *Fut8*^*+/+*^ mice (Fig. 4*A*). Interestingly, exogenous L-fucose was able to downregulate the gene expression of FcγRⅣ (Fig. 4*A*). Furthermore, the results obtained from Western blots demonstrated that the expression levels of FcγRⅣ protein were also increased in *Fut8*^*+/−*^ mice and it could be suppressed by exogenous L-fucose (Fig. 4*B*). These findings suggest that the decreased IgG levels in *Fut8*^*+/−*^ mice may be attributed to the overexpressed FcγRⅣ, and core fucosylation negatively regulates the expression of FcγRⅣ.

### Exogenous L-fucose upregulated the anti-ovalbumin-specific IgG levels in *Fut8*^*+/−*^ mice

**Figure 5:**
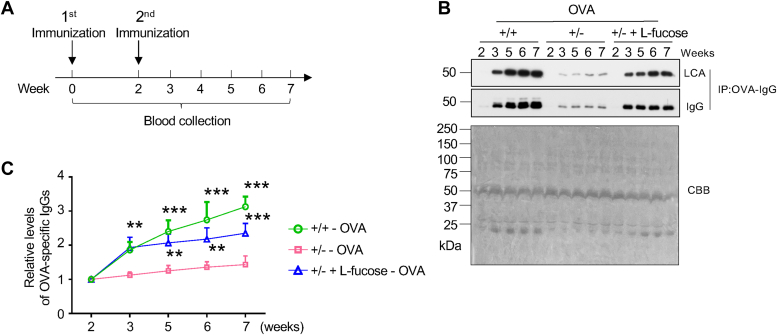
**Effect of exogenous L-fucose on the expression levels of specific anti-OVA antibodies.***A*, mice were treated with the immunization schedule with OVA and administrated with L-fucose at 0.4 mg/g/day. Blood was collected on the 0, second, third, fourth, fifth, sixth, seventh week post immunization. *B*, the OVA-specific IgGs were extracted as described in “Experimental procedures”. The levels of OVA-specific IgGs in an equal amount of serum (2 μl) were detected by anti-mouse IgG antibody, and the levels of core fucosylated IgG were detected using LCA lectin blot analysis. CBB was used as a loading control. *C*, comparison of expression levels of the OVA-specific IgGs among *Fut8*^*+/+*^, *Fut8*^*+/−*^, and *Fut8*^*+/−*^ mice treated with L-fucose. The data were obtained from at least three mice and analyzed by Image J using one-way ANOVA with Tukey’s *post hoc* analysis as the mean ± SEM. The relative levels of OVA-specific IgGs indicate the OVA-specific IgG levels *versus* the OVA-specific IgG levels at the second immunization point, which were set as 1.0. ∗∗*p* < 0.01; ∗∗∗*p* < 0.001. CBB, Coomassie brilliant blue; Fut8+/−, Fut8 heterozygous knockout; IgG, immunoglobulin G; LCA, *Lens culinaris* agglutinin; OVA, ovalbumin.

Different types of antigens lead to the production or switching of distinct IgG subclasses (1). OVA, a major protein found in egg whites, is commonly used as a model antigen to study the immune response. In general, OVA-induced IgG1 and IgG2a are the most studied subclasses in experimental models (39). Based on the observation in Figure 1, we were interested in whether the specific IgG levels could also change in response to antigen stimulation. Therefore, we stimulated mice using OVA and collected the sera described in “Experimental procedures” (Fig. 5*A*). After immunization, we extracted anti-OVA specific IgGs by binding them to OVA-immobilized N-hydroxy-succinimide (NHS) beads. The amounts of anti-OVA-specific IgG were significantly decreased in *Fut8*^*+/−*^ mice compared to *Fut8*^*+/+*^ mice (Fig. 5*B*). Importantly, exogenous L-fucose also increased the total amounts of anti-OVA-specific IgG, which was further confirmed by the time course for the IgG expression levels (Fig. 5, *B* and *C*). Moreover, the levels of core fucosylated IgG detected by LCA lectin also presented a significant increase in *Fut8*^*+/−*^ mice treated with L-fucose (Fig. 5*B*). As observed in Figure 4, we also examined the mRNA levels of FcγRs in the bone marrow of *Fut8*^*+/+*^ and *Fut8*^*+/−*^ mice stimulated by OVA. Although the mRNA expression levels of FcγRⅠ were also increased in *Fut8*^*+/−*^ mice, the mRNA expression levels of FcγRⅣ were significantly upregulated more than 30 folds in *Fut8*^*+/−*^ mice compared to *Fut8*^*+/+*^ mice, and these increases were suppressed by exogenous L-fucose (Fig. S17*A*). Moreover, the protein expression levels of FcγRⅣ were significantly elevated in *Fut8*^*+/−*^ mice. These increases were effectively suppressed by exogenous L-fucose (Fig. S17*B*). In summary, these data suggest that core fucosylation regulates specific-IgG levels through the Fc-FcγRⅣ degradation pathway, which can be modulated by exogenous L-fucose.

### Deficiency of core fucosylation or FcγRⅣ enhanced IgG internalization and transcytosis in BV-2 cells

**Figure 6:**
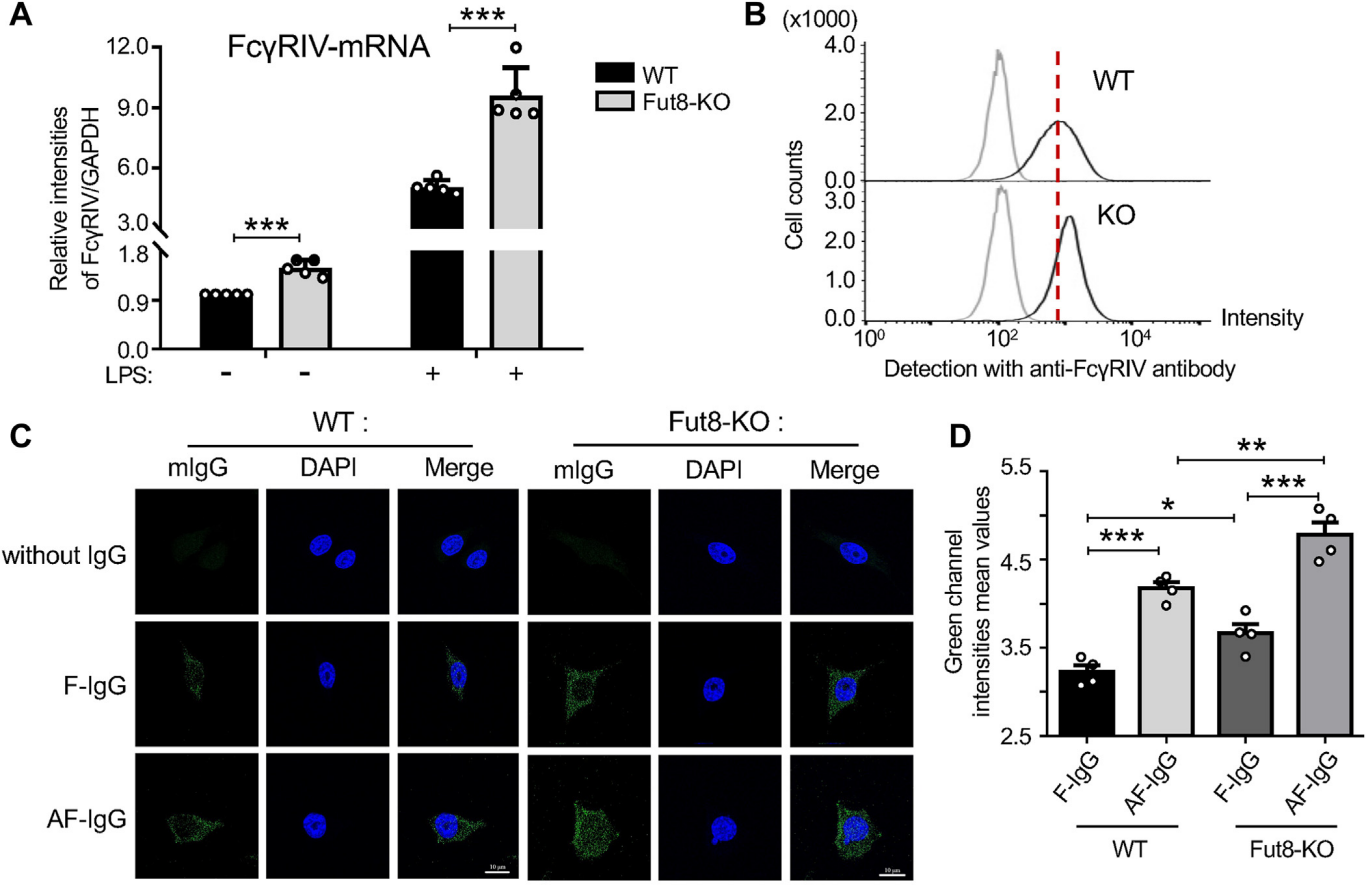
**Comparison of IgG internalization and transcytosis between WT and Fut8-KO BV-2 cells**. *A*, the cells were stimulated with or without LPS (250 ng/ml) for 4 h. Real-time PCR was used to detect the mRNA expression levels of FcγRIV, with GAPDH serving as an internal control. Each value was normalized to that of the GAPDH, and the value of WT cells stimulated without LPS was set as 1.0. The data representing relative intensities of FcγRIV/GAPDH were obtained from three independent experiments and subjected to qualification by an unpaired Student *t* test as the mean ± SEM. ∗∗∗*p* < 0.001. *B*, equal cell numbers (5 × 10^4^ cells) were collected, and each sample was divided into two tubes, one for negative control and another for detecting with Alexa Fluor 488 anti-mouse CD16.2 (FcγRIV) antibody *via* flow cytometry analysis. The negative controls were represented as *gray lines*, and the FcγRIV expression levels on the cell surface were depicted as *black*. *C*, representative confocal fluorescence images depict internalized IgG (*green*) in BV-2 cells, with nuclear staining with 4′,6-diamidino-2-phenylindole (DAPI) (*blue*). Scale bars are 10 μm. F-IgG indicates that the IgG was purified from the *Fut8*^*+/+*^ sera, while AF-IgG was from *Fut8*^*−/−*^ mice. *D*, the fluorescence intensity analysis of internalized IgG (*green*) was calculated using the ZEN 3.3 application. The *y*-axis represents fluorescence intensity mean values. Data were analyzed by one-way ANOVA with Tukey’s *post hoc* analysis as the mean ± SEM (n = 4). ∗*p* < 0.05, ∗∗*p* < 0.01, ∗∗∗*p* < 0.001. AF-IgG, noncore fucosylated IgG; *Fut8*^*−/−*^, *Fut8* knockout; FcγRⅣ, Fc-gamma receptor Ⅳ; IgG, immunoglobulin G; LPS, lipopolysaccharide.

FcγRⅣ is primarily expressed in monocytes/macrophages, mast cells, neutrophils, basophils, dendritic cells, and eosinophils (10). In this study, we compared the mRNA levels of FcγRⅣ between BV-2 (WT) cells and the Fut8 knockout BV-2 (Fut8-KO) cells, generated using the CRISPR/Cas9 system described previously (32). Real-time PCR results revealed higher mRNA levels of FcγRⅣ in Fut8-KO cells compared to WT cells. Moreover, this difference was further amplified with lipopolysaccharide (LPS) stimulation (Fig. 6*A*). Flow cytometry analysis also showed an upregulation of FcγRⅣ expression on the cell surface in Fut8-KO cells (Fig. 6*B*).

To understand the effects of core fucosylation on IgG internalization and transcytosis, we separately added the purified regular core fucosylated mouse IgG (F-IgG) from *Fut8*^*+/+*^ mice and noncore fucosylated IgG (AF-IgG) from *Fut8*^*−/−*^ mice to WT and Fut8-KO cell culture medium. The ability of IgG internalization and transcytosis was evaluated by immunofluorescence assay to monitor the fluorescence signals. Compared with F-IgGs, the AF-IgGs were more easily internalized and transcytosis, detected as small fluorescence dots in the WT cells (Fig. 6, *C* and *D*). Furthermore, this phenomenon was amplified in Fut8-KO cells (Fig. 6, *C* and *D*). These results suggest that core fucosylation closely regulates the ability of IgG internalization and transcytosis, which can be enhanced by a deficiency of Fut8. This may partly explain why the IgG amounts were decreased while the ratios of core fucosylated IgG *versus* total IgG remained the same, as shown in Figure 2.

On the other hand, we detected the mRNA levels of other main FcγRs besides FcγRⅣ. Unexpectedly, the real-time PCR results showed that the deficiency of Fut8 increased the expression levels of not only FcγRⅣ but also other FcγRs in BV2 cells (Fig. S18), which were inconsistent with the results *in vivo* (Fig. 4). In addition, the responses for LPS stimulation were different among these FcγRs (Fig. S18). Thus far, the underlying mechanisms remain a subject for further study.

**Figure 7:**
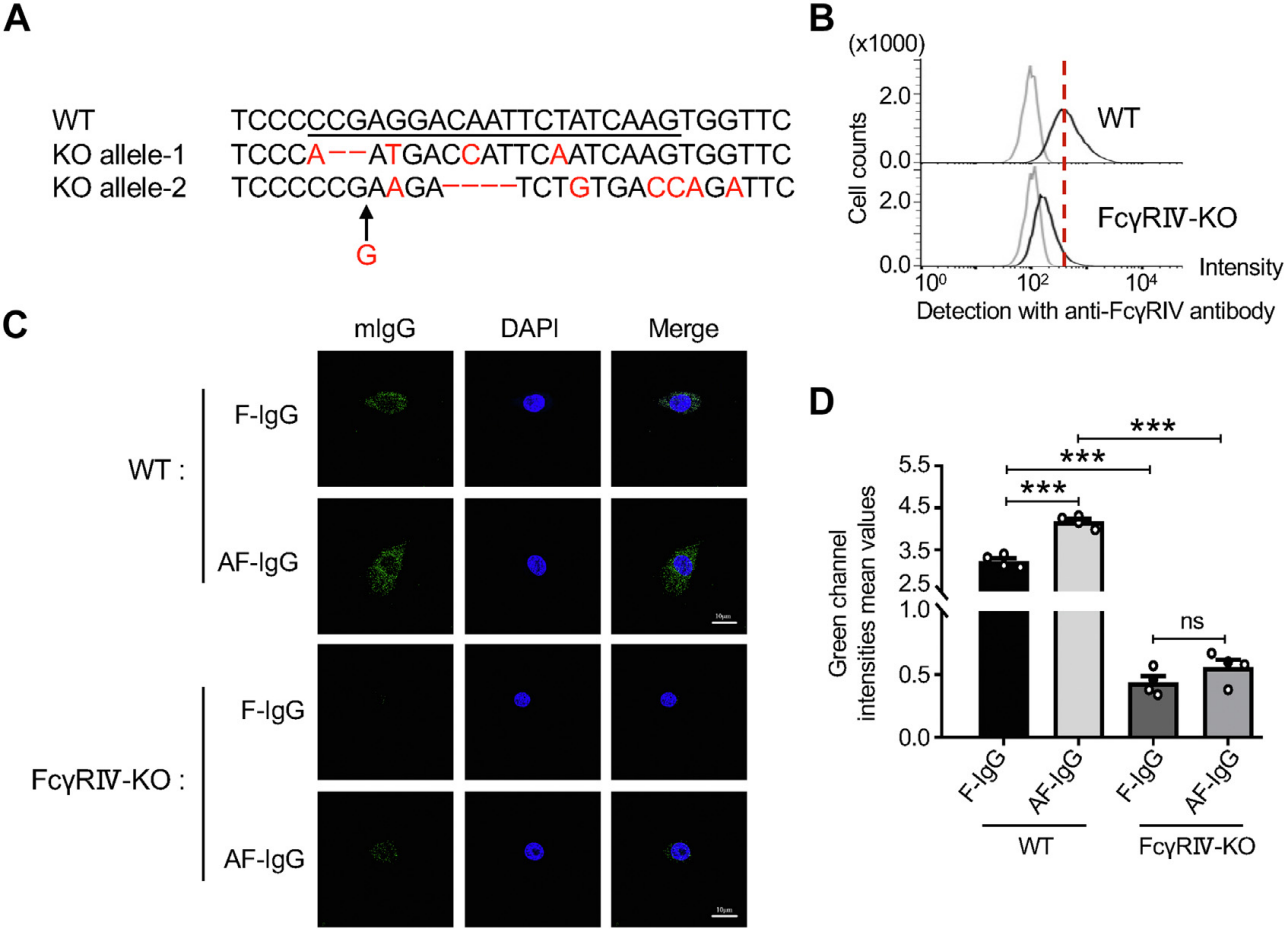
**Comparison of IgG internalization and transcytosis between WT and FcγRⅣ KO.***A*, the FcγRⅣ-targeting gRNA was designed (*underlined*). Compared to WT cells, the sequence of FcγRⅣ KO BV2 cells showed two bases (CG) deletion and four bases mutation (C was replaced by A, G was replaced by T, A was replaced by C, and T was replaced by A in the *red letter*) in allele 1 and 4-base (CAAT) deletion and six mutations (A was replaced by G, A was replaced by C, G was replaced by C, T was replaced by A, and G was replaced by A in the *red letter*) and a G insert between G and A in allele 2. *B*, the same number of cells were incubated with Alexa Fluor 488 anti-mouse CD16.2 (FcγRIV) antibody, and the expression of FcγRⅣ was verified by flow cytometry analysis. *C*, representative confocal fluorescence images depict internalized IgG (*green*) in BV-2 cells, with nuclear staining with DAPI (*blue*). Scale bars are 10 μm. F-IgG indicates that the IgG was purified from the *Fut8*^*+/+*^ sera, while AF-IgG was from *Fut8*^*−/−*^ mice. *D*, the *green* fluorescence intensities analysis of internalized IgG (*green*) was calculated using the ZEN 3.3 application. The *y*-axis represents fluorescence intensity mean values. Data were analyzed by one-way ANOVA with Tukey’s *post hoc* analysis as the mean ± SEM (n = 4). ns, no significance, *p* > 0.05, ∗∗∗*p* < 0.001. AF-IgG, noncore fucosylated IgG; *Fut8*^*−/−*^, Fut8 knockout; FcγRⅣ, Fc-gamma receptor Ⅳ; gRNA, guide RNA; IgG, immunoglobulin G; F-IgG, fucosylated mouse IgG.

To investigate the specific function of FcγRIV in IgG internalization, we established the FcγRⅣ knockout BV-2 (FcγRⅣ-KO) cell line and confirmed by the genomic sequence analysis (Fig. 7*A*) and flow cytometric analysis (Fig. 7*B*). Further, we compared IgG internalization and transcytosis between WT and FcγRⅣ-KO cells. As expected, the abilities of F-IgG internalization and transcytosis monitored by the fluorescence signals were almost lost in the FcγRⅣ-KO cells, while they were clearly observed in the WT cells (Fig. 7, *C* and *D*). The AF-IgG internalization and transcytosis were also significantly decreased in the FcγRⅣ-KO cells compared to the WT cells. These results further support that core fucosylation primarily regulates the IgG levels through the Fc-FcγRⅣ degradation pathway.

## Discussion

The present study demonstrated that a partial deficiency of Fut8 (*Fut8*^*+/−*^ mice) results in reduced IgG levels without affecting the ratio of core fucosylated IgG. We observed a decrease in IgG amounts in *Fut8*^*+/−*^ mice compared to the *Fut8*^*+/+*^ mice, which could be rescued by increasing core fucosylation by administering exogenous L-fucose. Furthermore, we observed an increase in the expression levels of FcγRⅣ, a receptor for the Fc region of mouse IgG, which mediates IgG endocytosis to induce cytokines in *Fut8*^*+/−*^ mice. This increase in FcγRⅣ expression was effectively suppressed by exogenous L-fucose. Furthermore, a deficiency of *FcγRⅣ* significantly suppressed the ability of IgG internalization and transcytosis. Given the critical roles of core fucosylation in IgG biology,where more than 95% of *N*-glycans on IgGs are core fucosylated (40), as also confirmed by the MS results in this study, our findings suggest potential roles of core fucosylation in immune responses, which may be regulated through the Fc-FcγRⅣ axis. Also, these observations may contribute to our understanding of the higher core fucosylation of IgG and its functional implications, such as stabilizing IgG in the serum and regulating various immune responses.

IgG is a class of antibodies produced by B cells in response to the presence of pathogens or foreign substances in the body. Mice possess distinct IgG subclasses: IgG1, IgG2a/b/c (depending on the mouse strain), and IgG3, each with unique functional roles underscored by their differential concentrations, synthetic rates, and biological half-lives (41). IgG has a longer half-life in the bloodstream than other antibody classes, partly due to its interaction with FcRn, which binds to the Fc region of IgG and protects it from degradation. This interaction allows IgG to be recycled back into the bloodstream after being taken up by cells (34). While IgG molecules are highly stable, they can undergo degradation in various ways over time or under certain conditions. For example, when IgG binds to antigens, it can be internalized and degraded within immune system cells through binding to FcγRs (38). Many factors can impact IgG abundance and FcγRs (42). Some studies have reported that deleting FcγRI can increase IgG subclass levels 2 to 5 times after immunization (43). IgG2 is the most abundant subclass, with a specific affinity for FcγRⅣ (11). Therefore, the decrease in IgG levels observed in *Fut8*^*+/−*^ mice may be explained by regulating FcγRIV expression levels by core fucosylation, as shown in Figures 4 and S1. Of course, we could not exclude other possibilities since the mechanisms for the production of specific IgG should be complicated. Previous studies showed that core fucosylation of IgG B cell receptor plays an important role in antigen recognition and antibody production (44). CD4^+^ T cells, as helper T cells, assist in the activation of B cells, and activated CD4^+^ T cells could stimulate the B cell to proliferate and differentiate into plasma cells through releasing cytokine (45). Deficiency of core fucosylation suppressed the activation of CD4^+^ T cells and attenuated the interaction of T-B cells, which is also an important mechanism in regulating the IgG amounts (46).

Core fucosylation has been identified as a critical regulator in modulating the activity of specific immune cells, such as T cells and macrophages. Our previous studies showed that a deficiency of core fucosylation induced an emphysema-like phenotype in Fut8^−/−^ mice (47) and suppressed transforming growth factor-β-mediated signaling, which regulates M2 macrophage activation. Although the underlying mechanisms remain unclear, based on observation in the present study, we can speculate that a lack of core fucosylation significantly enhances M1 macrophage activation, leading to the release of matrix metalloproteinases and the development of an emphysema-like phenotype in Fut8^−/−^ mice. Many monocytes were found to be infiltrated in the lung tissues of Fut8^−/−^ mice (47). Recent studies have also shown that Fut8 negatively regulated M1 macrophage activation (48). Interestingly, it is known that the activation of M1 macrophage can selectively increase the IgG2a FcγR (49), which can be upregulated by several cytokines. Important cytokines in M1 polarization of macrophages, such as interferon gamma and tumor necrosis factor-α, can promote the expression of FcγRII (50).

Additionally, a potential immunotherapeutic agent, interleukin (IL)-15, can enhance the expression of FcγRIV and promote the interaction of macrophages and natural killer cells (51). In this study, the treatment with LPS could induce the expression of FcγRIV in BV-2 cells, and IgG internalization and transcytosis were significantly enhanced in the Fut8-KO cells (Fig. 6). The core fucosylation on inflammation may exhibit cell or tissue-specific variations. Recently, we found that core fucosylation has a negative regulatory effect on inflammation in lung and brain tissues while a positive regulatory effect in the spleen (32). In brain tissues, the decreased core fucosylation leads to the upregulation of complex formation between gp130 and IL-6 receptors and enhances downstream signaling, such as phosphorylation of JAK2, Akt, and STAT3, which can be reduced by exogenous L-fucose (32). Considering cytokine signaling can induce the expression of FcγRs (49, 50), we speculate that exogenous L-fucose suppresses FcγRIV expression partially, at least through the proinflammatory signal pathways such as IL-6 signaling, modulated by core fucosylation of gp130.

The positive impact may be attributed to its ability to positively regulate CD14 through toll-like receptor 4 signaling (52, 53). In addition, curiously, Jin , *et al.* reported that the Fut8-catalyzed core fucosylation positively regulated amyloid-β oligomer-induced microglia activation using human induced pluripotent stem cells-derived microglia (54). Although the underlying mechanisms of core fucosylation on inflammation remain unclear, we speculate that core fucosylation may increase IgG levels by reducing the expression of FcγRIV, potentially through the modulation of cytokines associated with macrophage polarization.

It is worth noting that most immune molecules, including FcγRs, are glycosylated (55). FcγRIV, for instance, has three *N*-glycosylated sites, while the homologous human FcγRIIIa (CD16a) has five *N*-glycosylated sites, which are crucial for their stability and activity (11, 55). Research has shown that the presence or absence of glycans at these *N*-linked sites can affect the binding of FcγRIIIa to IgG antibodies. Specifically, the presence of glycans at position 162 enhances the binding of FcγRIIIa to IgG, while the presence of glycans at position 45 inhibits this binding (56, 57, 58). Among the subclasses, IgG2 shows a higher affinity for binding to FcγRIV in mice (10). Afucosylated mIgG2 displays a 10-fold increased affinity and is particularly inclined to bind to FcγRIV (11), resulting in enhanced ADCC (25). However, it is important to note that afucosylated IgG antibodies, while more potent in activating immune cells, can potentially lead to increased inflammation and may induce specific side effects, such as thrombotic (59) and graft injury (60). Therefore, there are no significant changes in the ratios of core fucosylated *versus* total IgG in *Fut8*^*+/+*^ and *Fut8*^*+/−*^ mice with or without L-fucose (Fig. 2), which could be very meaningful. The highly expressed afucosylated IgG can increase IgG-FcγR binding to induce cytokine production, which may harm tissue physiology, as mentioned above as well as in the Introduction (18, 26, 27).

Exogenous L-fucose, serving as the substrate for the formation of core fucosylation (29), can increase the core fucosylation levels through the salvage pathway, consequently enhancing the immune response (61, 62). Reports have indicated that L-fucose can influence macrophage polarization (63, 64), and it is considered an effective treatment for safely augmenting intratumoral immune cells and enhancing immunotherapy efficacy in conditions such as melanoma (62). In our study, we observed that exogenous L-fucose effectively increased the level of core fucosylation (Fig. 1) and reversed the decreased IgG levels in *Fut8*^*+/−*^ mice through the downregulation of FcγRIV expression (Figs. 4 and S17). These results collectively demonstrate that core fucosylation plays a critical role in regulating IgG levels. Exogenous L-fucose is a valuable tool for enhancing IgG levels, further influencing adaptive immune responses. This insight may have far-reaching implications in the field of medical treatments, particularly in the realms of cancer therapy and autoimmune diseases.

## Experimental procedures

### Antibodies and reagents

The experiments were conducted using the following antibodies and reagents: Biotinylated *Lens culinaris* agglutinin (LCA) (J207), which preferentially recognizes core fucose (65), was obtained from J-oil Mills. The anti-GAPDH antibody (G9545), ovalbumin (OVA) (A5503), complete Freund's adjuvant (344289), and incomplete Freund's adjuvant (344291) were from Sigma-Aldrich. The secondary antibody about horseradish peroxidase-conjugated goat against rabbit (#7074) was purchased from Cell Signaling Technology. Ab-Capcher MAG2 was purchased from ProteNova. ABC kit (PK-4000) was from Vector Laboratories. The Alexa Fluor 488 anti-mouse CD16.2 (FcγRIV) antibody (149524) was from Biolegend. L-fucose (F0065) was purchased from TCI. The anti-FcRn antibody (ab228975) was from Abcam. The streptavidin conjugate Alexa Fluor 647 antibody was from Invitrogen. The Goldenrod Animal Lancet (18310300) was from the Bio Research Center.

### Animals

All animal experiments adhered to protocols approved by the Animal Care and Use Committee of the Graduate School of Pharmaceutical Sciences, Tohoku Medical and Pharmaceutical University. *Fut8*^*+/+*^ littermates and *Fut8*^*+/−*^ mice were obtained by intercrossing the Institute of Cancer Research mice genetic background heterozygous mice (66). All experiments were conducted with 6-week-old mice. The mice were housed in groups under standard vivarium conditions, including a 12-h light/dark cycle, with lights on from 7:00 to 19:00, an ambient temperature of 22 ± 2 °C, and a relative humidity of 55 ± 5%. They had free access to food and water.

### Submandibular bleeding method

We developed a more rapid and humane method to draw blood samples from mice. Blood was collected from the orbital venous plexus using a sterile, single-use mouse bleeding lancet (67). After collecting the blood, it clotted for 30 min at room temperature (RT). Subsequently, the serum was obtained from the clot by centrifuging at 2000*g* for 10 min.

### Animal immunization

Mice were immunized by subcutaneous injection with 100 μg OVA mixed with an equivalent volume of complete Freund's adjuvant. Two weeks later, mice were subcutaneously injected with 100 μg of OVA mixed with an equal volume of incomplete Freund's adjuvant. Mice sera were collected at 0, second, third, fourth, fifth, sixth, and seventh week post immunization.

### Immobilization of OVA on NHS beads

OVA (50 μg) was dissolved in 200 μl of immobilization buffer (25 mM Hepes-NaOH at pH 7.0) and immobilized on NHS beads using a microtube mixer TM-282 (AS ONE) at 4 °C. After incubation for 30 min, the mixture was centrifuged at 15,000 rpm for 5 min at 4 °C to remove the supernatant. The beads were incubated with the blocking buffer (1 M aminoethanol, 0.1% NP-40) using a microtube mixer TM-282 at 4 °C overnight, then centrifuged at 15,000 rpm for 5 min at 4 °C to remove the supernatant. The OVA-NHS beads were stored in storage buffer (10 mM Hepes-NaOH at pH 7.9, 50 mM KCl, 1 mM EDTA, and 10% glycerol) at 4 °C.

### Establishment of FcγRⅣ-KO cell line

The pSpCas9(BB)-2A-GFP (PX458) plasmid was acquired from Addgene (PX458: Addgene #48138). The FcγRⅣ-KO cell was constructed by guide RNA (5′-CCGAGGACAATTCTATCAAG-3′), targeted to the FcγRⅣ gene localized adjacent to Cas nine in the pSpCas9(BB)-2A-GFP vector. The BV2 FcγRⅣ-KO cell line was established by electroporating cells and performed according to the manufacturer's recommendations (Amaxa cell line Nucleofector kit; Lonza). Twenty-four hours post transfection, the cells with positive fluorescence were sorted using the FACSAria II (BD Biosciences). After sorting the GFP-expressing cells, each signal GFP-positive cell was seeded into a 96-well plate and cultured. After incubation for 3 weeks, those single clones were expanded. We extracted the genome and validated the CRISPR target region by PCR amplification using the following primers: forward primer, 5′-GTGCTTCCCTGCCTAGATACA-3′; reverse primer, 5′-GGTCACTGATCGTGGAGAGG-3′, and then sequenced with the forward primer.

### Western blot, lectin blot, and immunoprecipitation

For bone marrow tissue extraction, tibia and femur bone were harvested bilaterally, and bone marrow was flushed out with a syringe filled with RPMI 1640 medium containing 10% fetal bovine serum (68). The tissues were then homogenized and lysed in the cell lysate buffer (20 mM Tris–HCl, pH 7.4, 150 mM NaCl, 1% Triton X-100), including 1% protease and phosphatase inhibitors (Nacalai Tesque) for 30 min on ice. After centrifugation at 15,000 rpm for 15 min, the supernatants were collected, and the concentration was detected by the bicinchoninic acid protein assay kit (Pierce Manufacturing). Equal amount of proteins (10 μg) were used for Western blot and lectin blot analysis.

Western blot and lectin blot were performed as follows: proteins (10 μg) or immunoprecipitants (10 μl) were equally loaded into 7.5% or 12% SDS-PAGE at 100 V and then transferred to polyvinylidene difluoride membranes (Millipore Sigma) at 10 V for 1 h. After blocking (5% bovine serum albumin (BSA) for lectin blot/5% nonfat dry milk for Western blot) for 1 h at RT, the membranes were stained with LCA lectin or indicated primary antibodies at 4 °C overnight. After washing four times, the membranes were incubated with appropriate secondary antibodies. Immunoreactive bands were detected using an immobilon Western Chemiluminescent HRP Substrate (Millipore) based on the manufacturer's instructions.

For immunoprecipitation, 2 μl serum was combined with 10 μl Ab-Capcher MAG2 or OVA-immobilized beads at 4 °C for 2 h using a microtube mixer TM-282. After washing the mixture three times, the immunoprecipitates were detected by Western blotting and LCA lectin blotting.

### LC–ESI MS glycoproteomic analysis of IgG

The purified IgG proteins were dissolved in a denaturing solution, reduced with DTT, and alkylated with iodoacetamide. The proteins were digested with trypsin after desalting using a NAP-5 gel filtration column according to previous procedures (69). Tryptic peptides were dried by SpeedVac without the process of glycopeptide enrichment for the subsequent liquid chromatography electrospray ionisation tandem mass spectrometry analysis (69). Monoisotopic masses were assigned with possible monosaccharide compositions on peptide using the GlycoMod software tool (mass tolerance for precursor ions is ±0.01 Da; https://web.expasy.org/glycomod/). Xcalibur software, version 2.2 (Thermo Fisher Scientific), was used to show extracted ion chromatogram to analyze MS and MS/MS data. The relative abundances (%) of each glycan structure on each peptide were calculated by setting the total peak intensities of all detected glycopeptides on each *N*-glycan binding site in each extracted ion chromatogram as 100%.

### Flow cytometry analysis

Single-cell suspensions of bone marrow were prepared by gently grinding with frosted slides and then filtered through 39-μm nylon mesh. After washing with ice-cold PBS, the cells were resuspended at 1 × 10^6^ cells/ml density and incubated with biotinylated LCA and Alexa Fluor 488 anti-mouse CD16.2 (FcγRIV) antibody in 0.1% BSA in PBS for 1 h on ice. Subsequently, the cells were incubated with streptavidin conjugate Alexa Fluor 647 (1:500) for 25 min on ice in the dark. Then, the cells were washed and resuspended in 1 ml 0.1% BSA in PBS. The fluorescence intensities were detected by Attune flow cytometer (BD Biosciences) following flow cytometry experiment standard (70), and we analyzed the positive cells in the monocytes class of bone marrow cells (71) using FlowJo software (https://www.flowjo.com/).

### Real-time PCR (quantitative PCR)

RNAs were extracted with TRIzol reagent (Invitrogen), and 1 μg of total RNA was reverse-transcribed into complementary DNA by PrimeScript RT reagent with genomic DNA Eraser (Takara) according to the manufacturer's instructions. The sequences of those primers are listed in Table 1. The PCR products were diluted to 50 ng/μl and then detected by StepOnePlus (Applied Biosystem). The real-time PCR assays were performed using a TB Green Premix Ex Taq II (Tli RNaseH Plus) (Takara), and the conditions were as follows: inactivation of RTase at 95 °C for 10 s, then 40 cycles of denaturation at 95 °C for 5 s followed by annealing and extension at 60 °C for 30 s.

### Cell lines and cell culture

The mouse microglia cell line BV-2 cells were kindly provided by Professor Elisabetta Blasi (University of Modena and Reggio Emilia, Modena, Italy). BV-2 Fut8-KO cells were established using the CRISPR/Cas9 system as previously described (32). Cells were cultured in Dulbecco's modified Eagle's medium with 10% fetal bovine serum under a standard atmosphere at 37 °C and 5% CO_2_. These cells were free from *mycoplasma*, which was validated by the e-Myco *Mycoplasma* PCR Detection kit (iNtRON Biotechnology).

### IgG internalization and immunofluorescence

IgGs were purified from the *Fut8*^*+/+*^ and *Fut8*^*−/−*^ mice sera through pulling down by Ab-Capcher MAG2, as described previously, and quantified by bicinchoninic acid protein quantification protocol (Thermo Fisher Scientific). Equal cells (5 × 10^4^ cells) were cultured on glass-bottom dishes treated with 0.3 μg/ml IgG (from *Fut8*^*+/+*^ or *Fut8*^*−/−*^ mice) for 25 min. After incubation, the cells were washed with PBS to remove excess IgG in the culture medium. The cells were fixed with 4% paraformaldehyde for 30 min. Subsequently, cells were treated with 0.1% TritonX-100 in PBS for 10 min and then incubated with 5% BSA in PBS at RT for 2 h to block nonspecific staining. Finally, the cells were incubated with the goat anti-mouse IgG Alexa Fluor 488 for 1 h and 4′,6-diamidino-2-phenylindole for 8 min in the dark at RT. Detection was performed using a ZEISS LSM 900 confocal microscope objective Plan-Apochromat 63x/1.4 Oil M27 (FWD = 0.19 mm).

### Statistical analysis

All data are presented as the mean ± SEM obtained from at least three independent experiments. Statistics analysis was performed using a one-way analysis of variance (ANOVA) with Tukey's *post hoc* test or an unpaired Student *t* test by GraphPad Prism 6.0 software (GraphPad Software [www.graphpad.com], Inc). A probability value of *p* was considered as follows: ns (no significance). *p* > 0.05; ∗*p* < 0.05; ∗∗*p* < 0.01; ∗∗∗*p* < 0.001.

## Data availability

All data were provided in the figures, tables, and supplementary information in this manuscript. Glycoproteomic raw MS data and the identification result file for analysis of glycan structures on peptides have been deposited at the GlycoPOST (announced ID: GPST000407).

## Supporting information

This article contains supporting information.

## Conflicts of interest

The authors declare that they have no conflicts of interest with the contents of this article.
