# Supplementary material for: Core fucosylation within the Fc-FcγR degradation pathway promotes enhanced IgG levels via exogenous L-fucose
Source: J Biol Chem. 2024 Jul 11;300(8):107558. doi: 10.1016/j.jbc.2024.107558 (PMC11345378; doi:10.1016/j.jbc.2024.107558)
Supplement: Supplementary Figures [file mmc1.pdf]

Supplemental Fig. 1

Base Peak Chromatogram (BPC) in MS

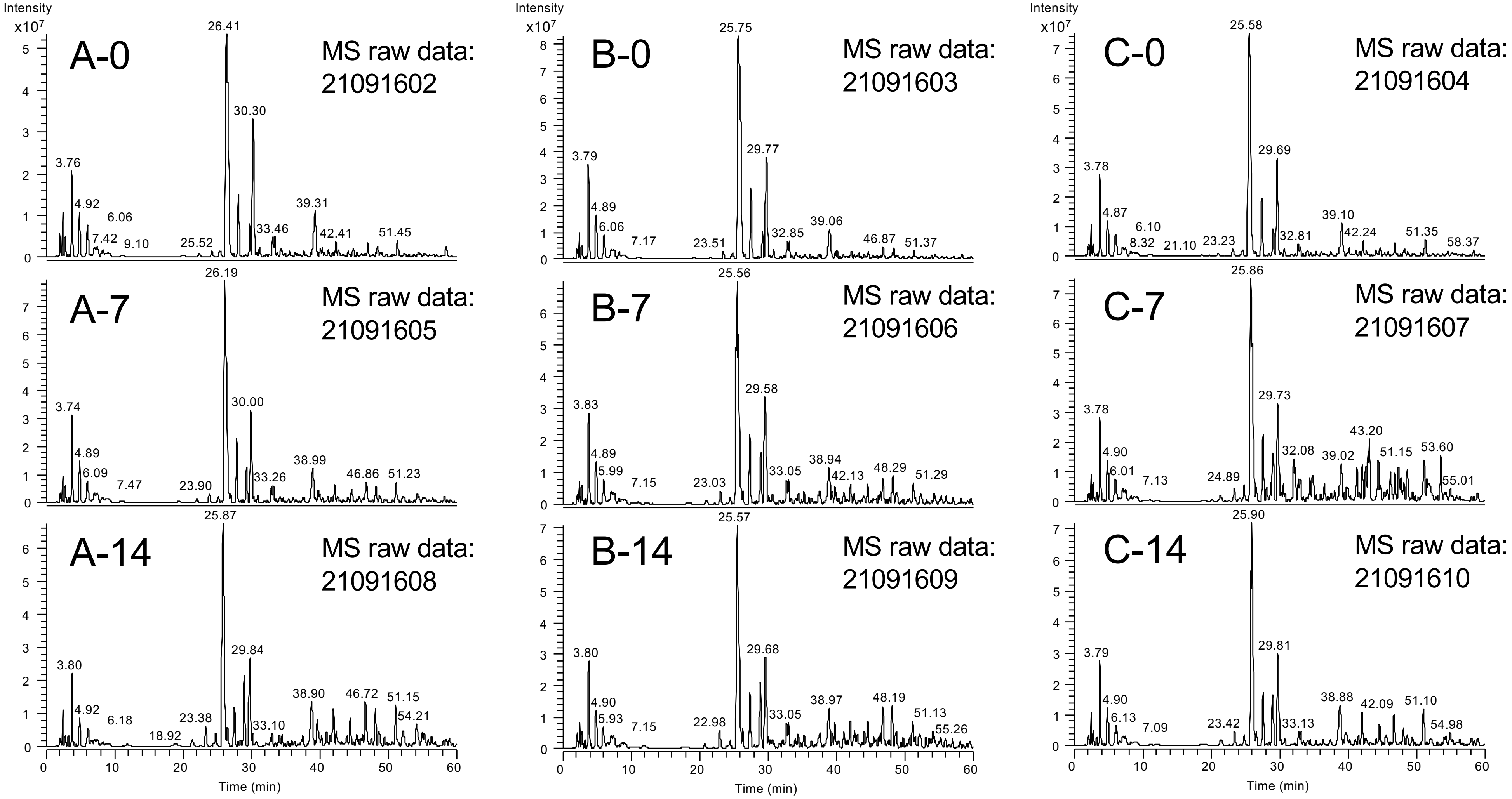

These glycoproteomic raw MS data and the identification result file for analysis of glycan structures on peptides have been deposited to the GlycoPOST (announced ID: GPST000407).

Supplemental Fig. 2 A-0: before treatment with 0.15 mg/g/day L-fucose (0th day) MS raw data: 21091602

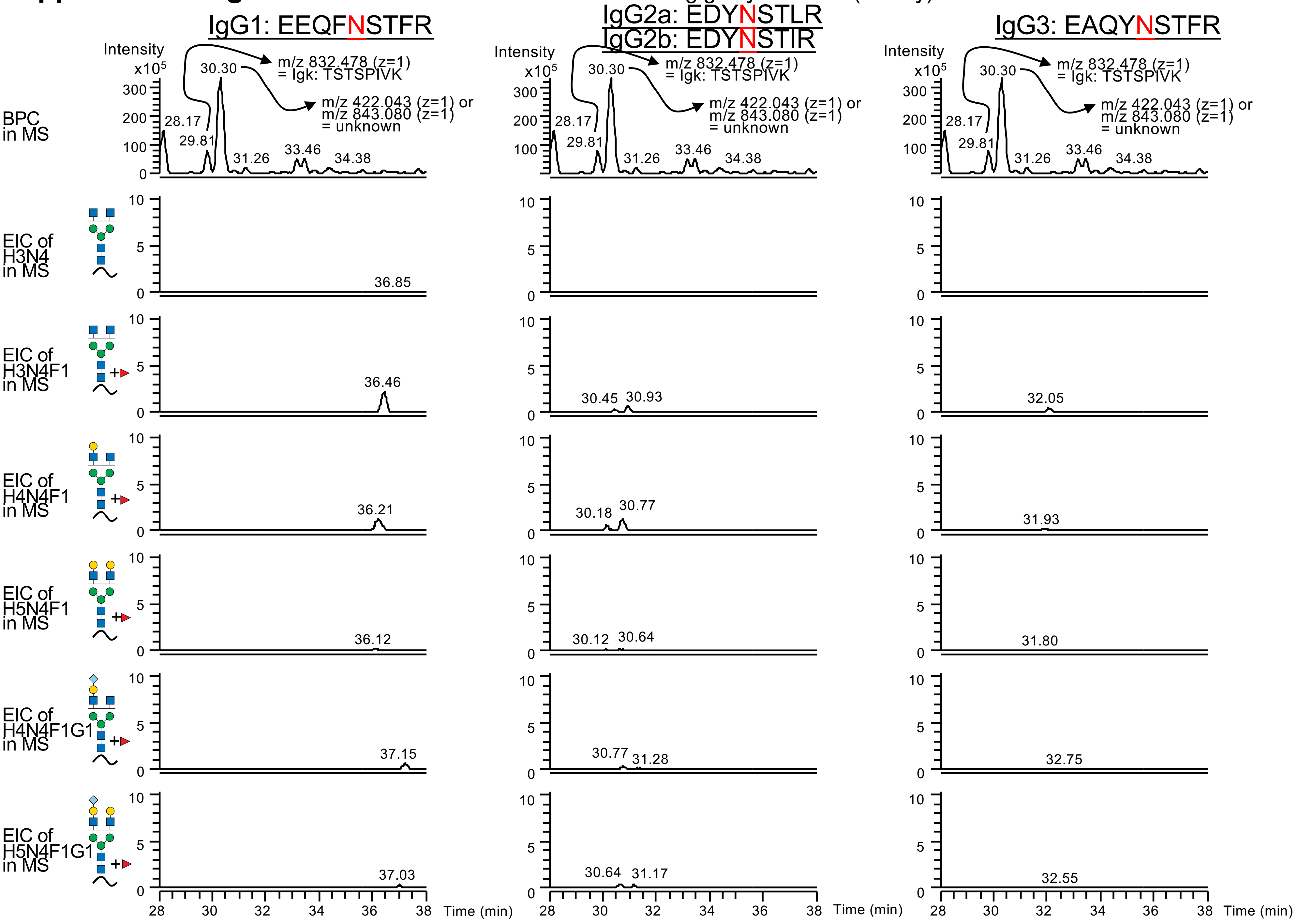

LC-MS analysis of *N*-glycans on IgGs obtained from *Fut8*+/– mice before treatment with 0.15 mg/g/day L-fucose_0th day (A-0).

Supplemental Fig. 3

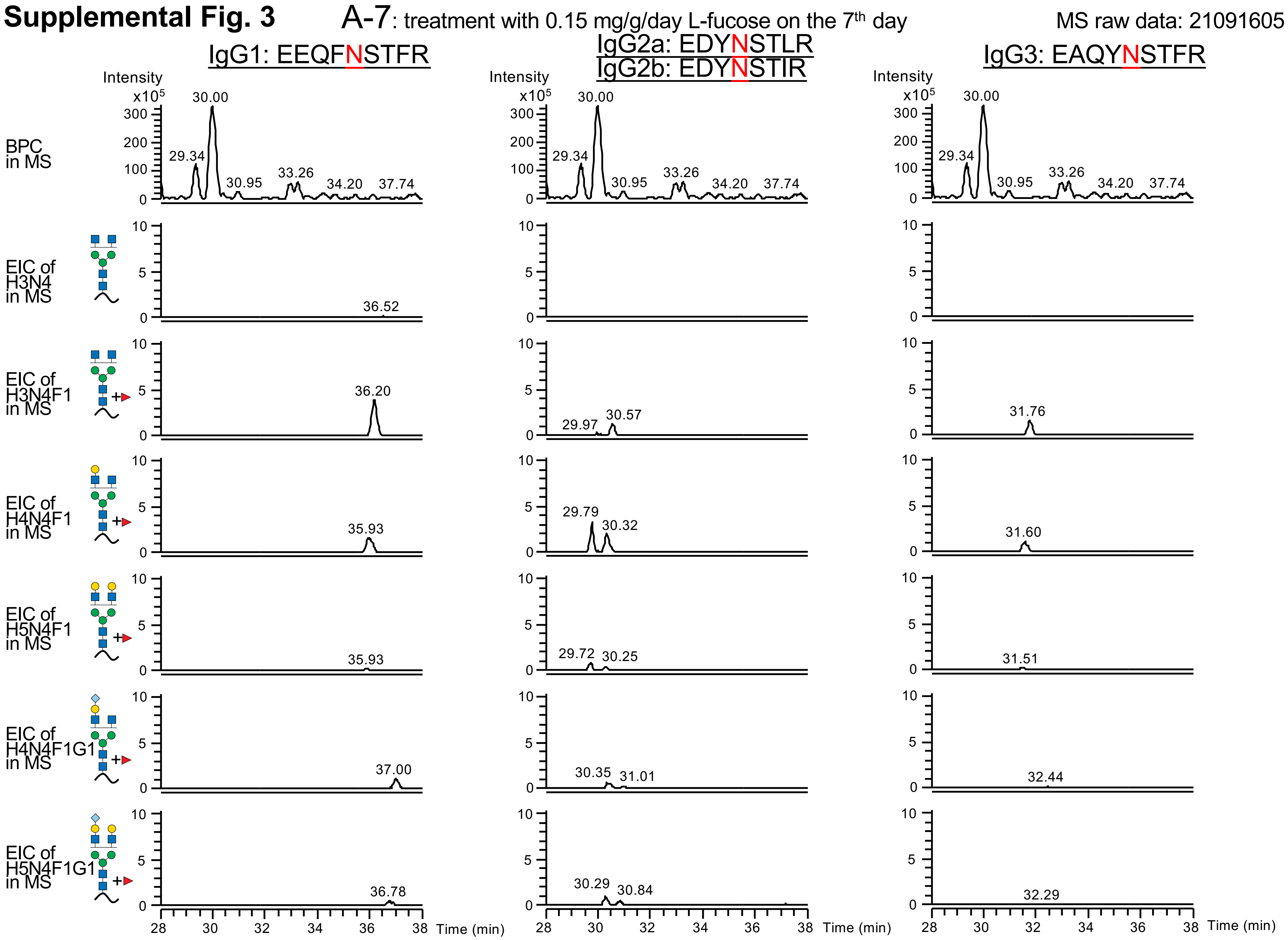

LC-MS analysis of *N*-glycans on IgGs obtained from *Fut8*$^{+/-}$ mice treated with 0.15 mg/g/day L-fucose on the 7th day (A-7).

Supplemental Fig. 4

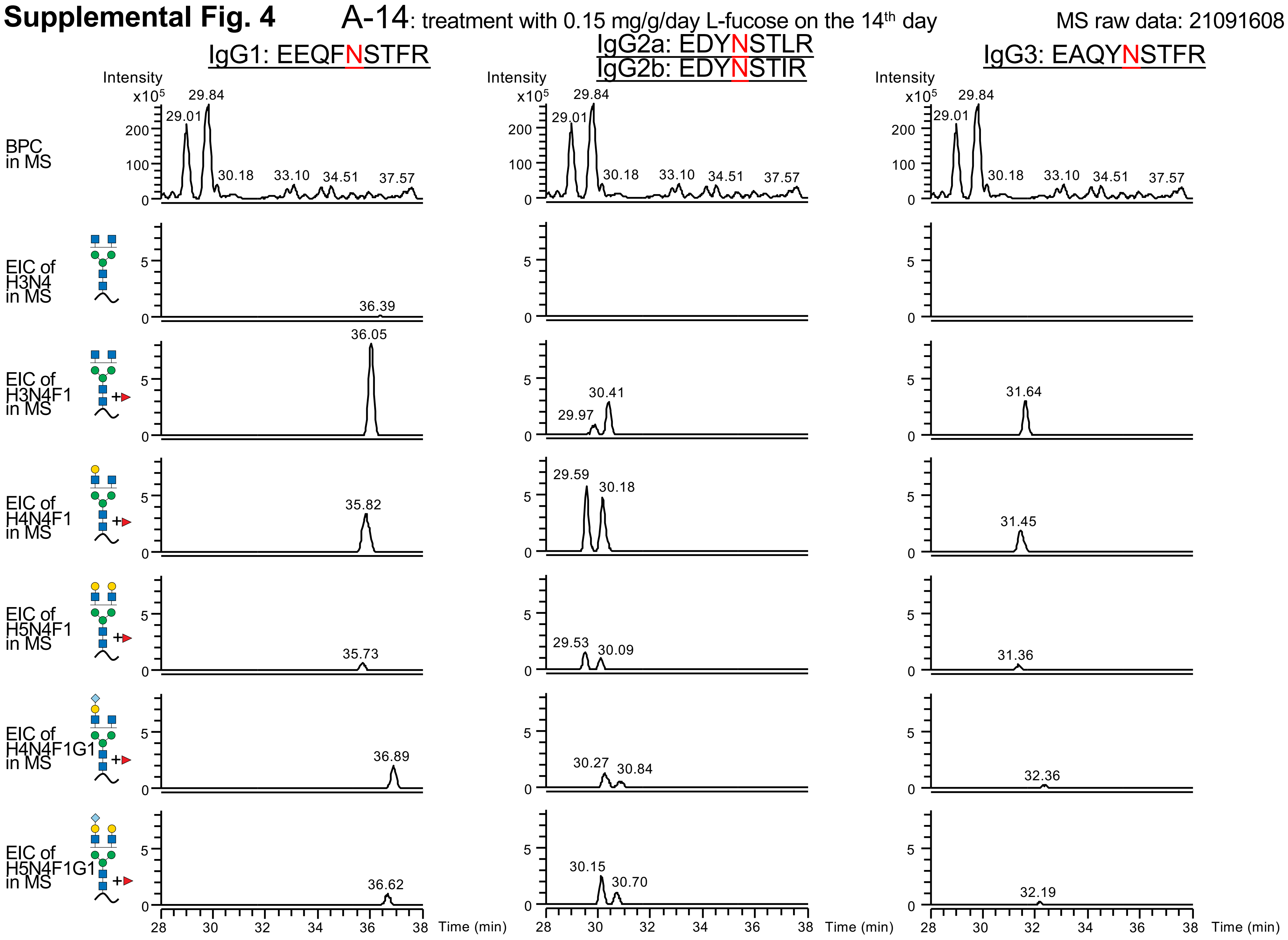

LC-MS analysis of *N*-glycans on IgGs obtained from *Fut8*$^{+/-}$ mice treated with 0.15 mg/g/day L-fucose on the 14th day (A-14).

Supplemental Fig. 5 B-0: before treatment with 0.4 mg/g/day L-fucose (0th day) MS raw data: 21091603

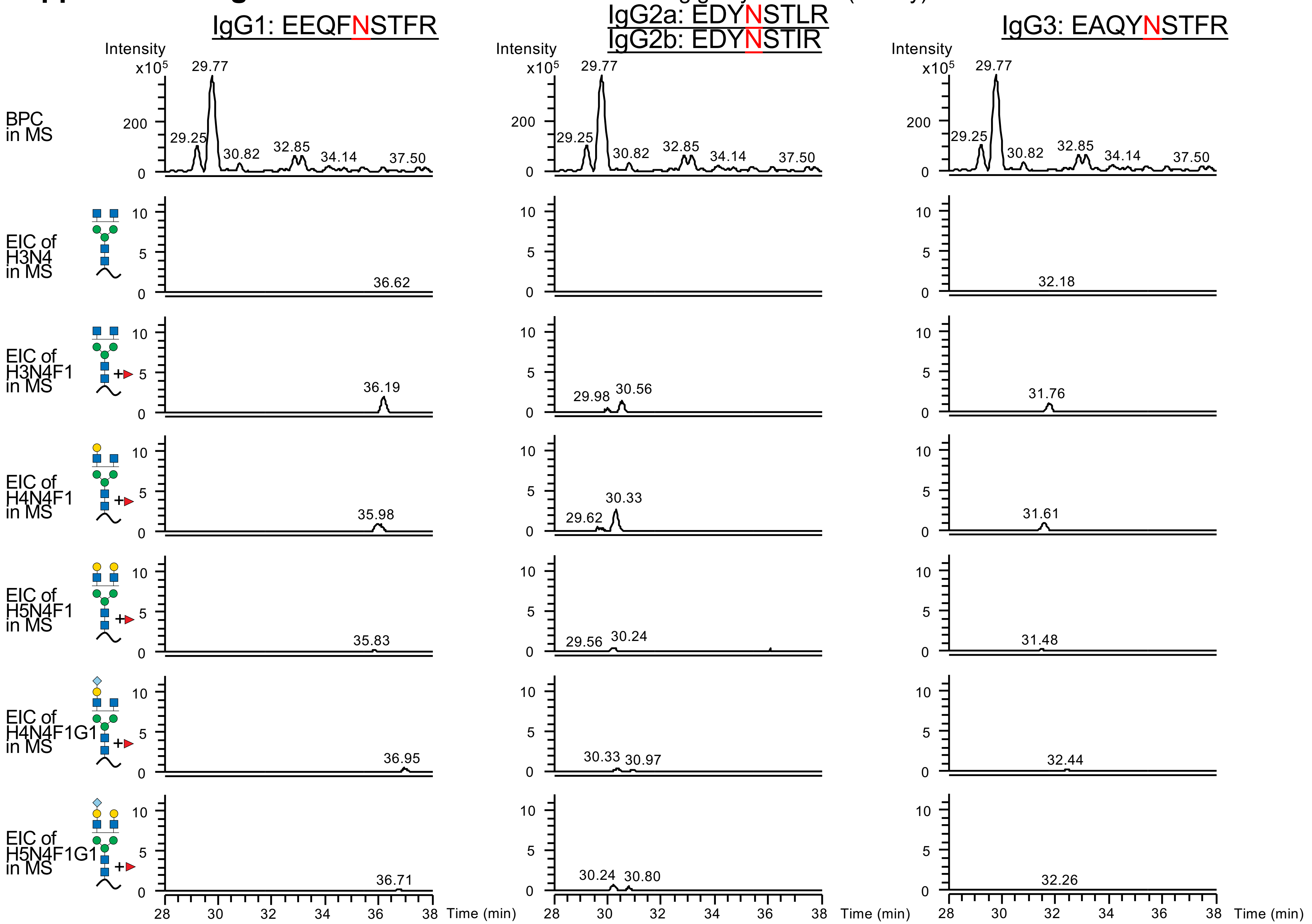

LC-MS analysis of *N*-glycans on IgGs obtained from *Fut8*+/− mice before treatment with 0.4 mg/g/day L-fucose_0th day (B-0).

Supplemental Fig. 6

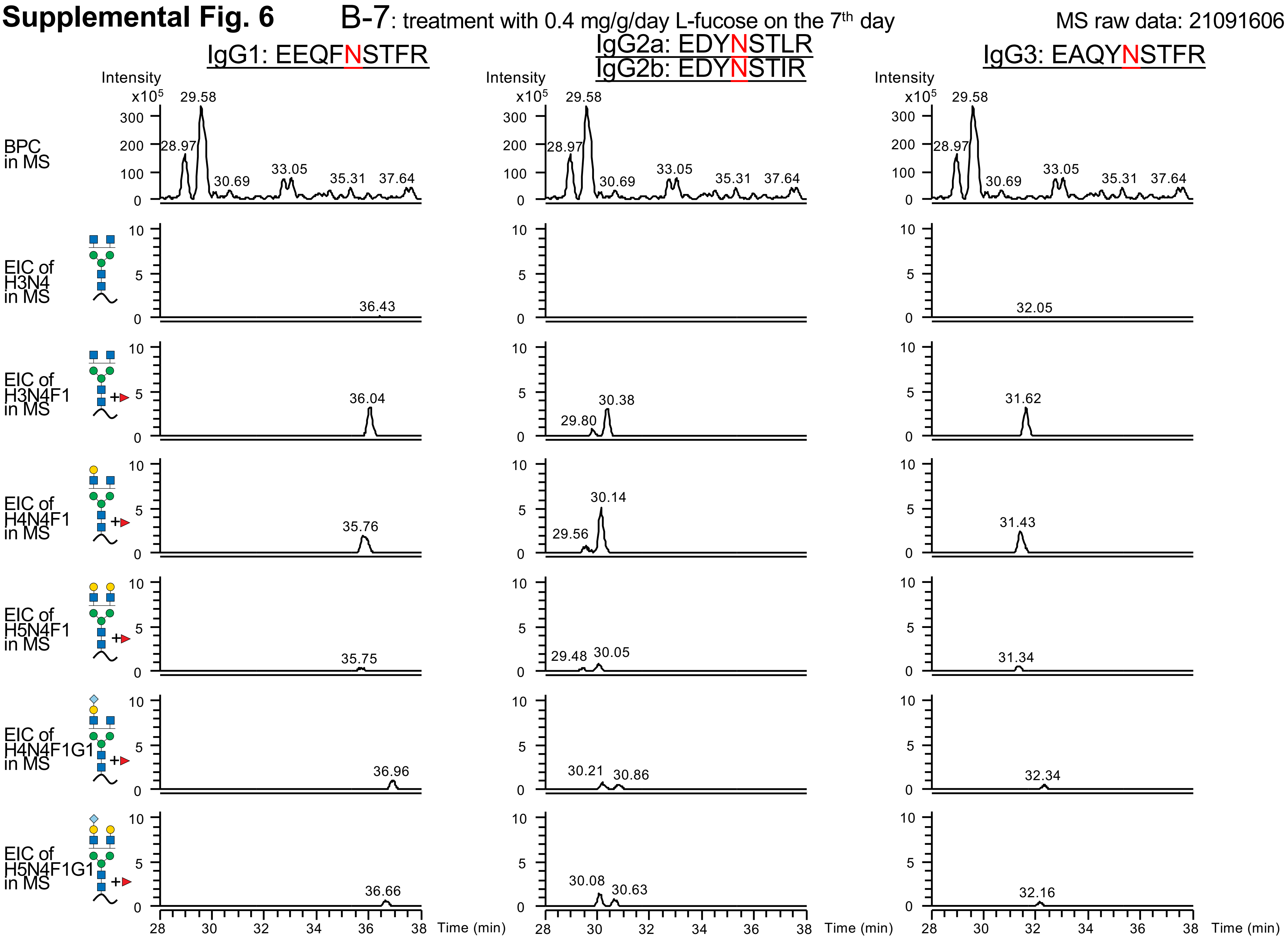

LC-MS analysis of *N*-glycans on IgGs obtained from *Fut8*+/– mice treated with 0.4 mg/g/day L-fucose on the 7th day (B-7).

Supplemental Fig. 7

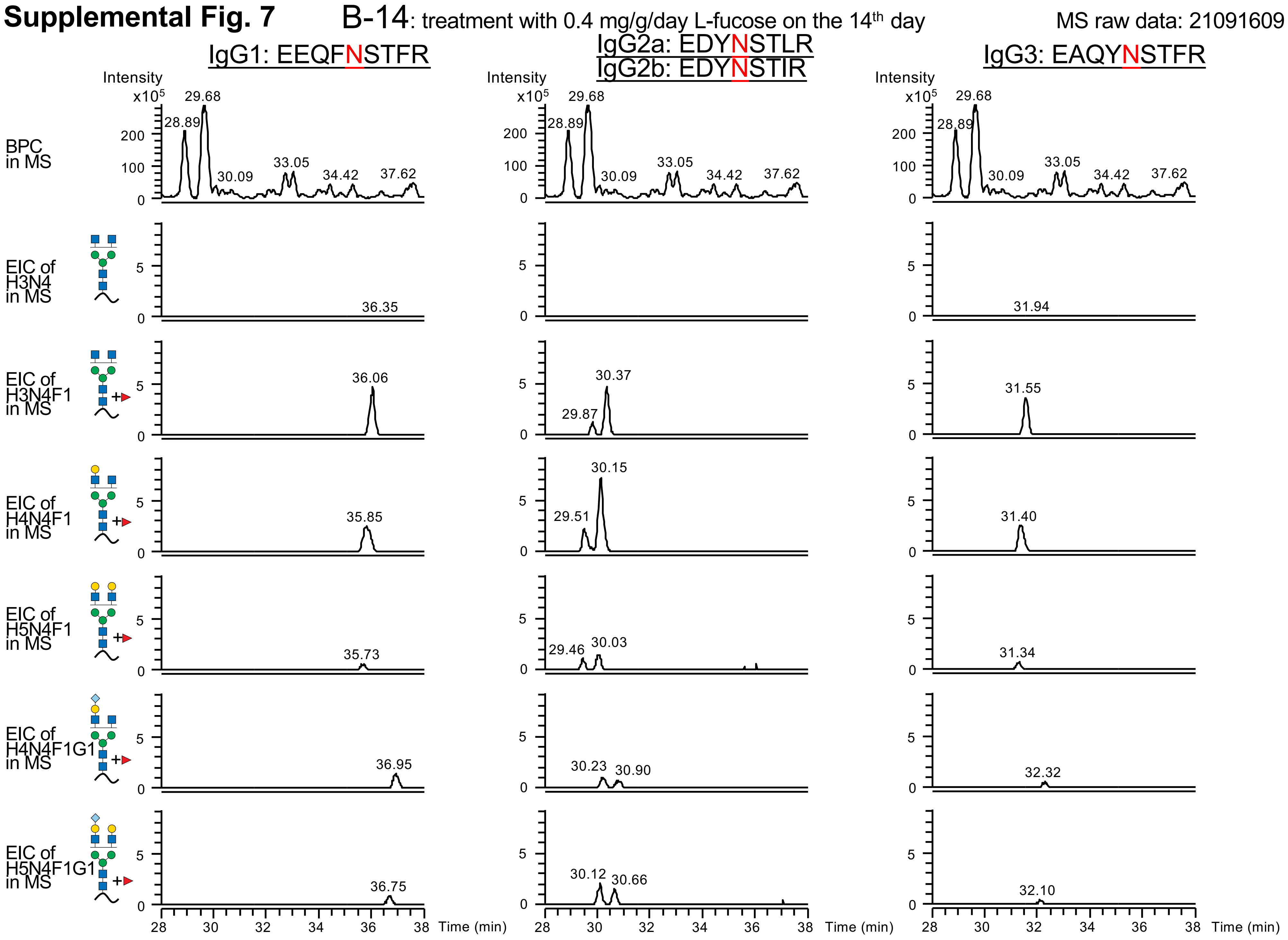

LC-MS analysis of *N*-glycans on IgGs obtained from *Fut8*+/− mice treated with 0.4 mg/g/day L-fucose on the 14th day (B-14).

Supplemental Fig. 8 C-0: before treatment with 1.2 mg/g/day L-fucose (0th day) MS raw data: 21091604

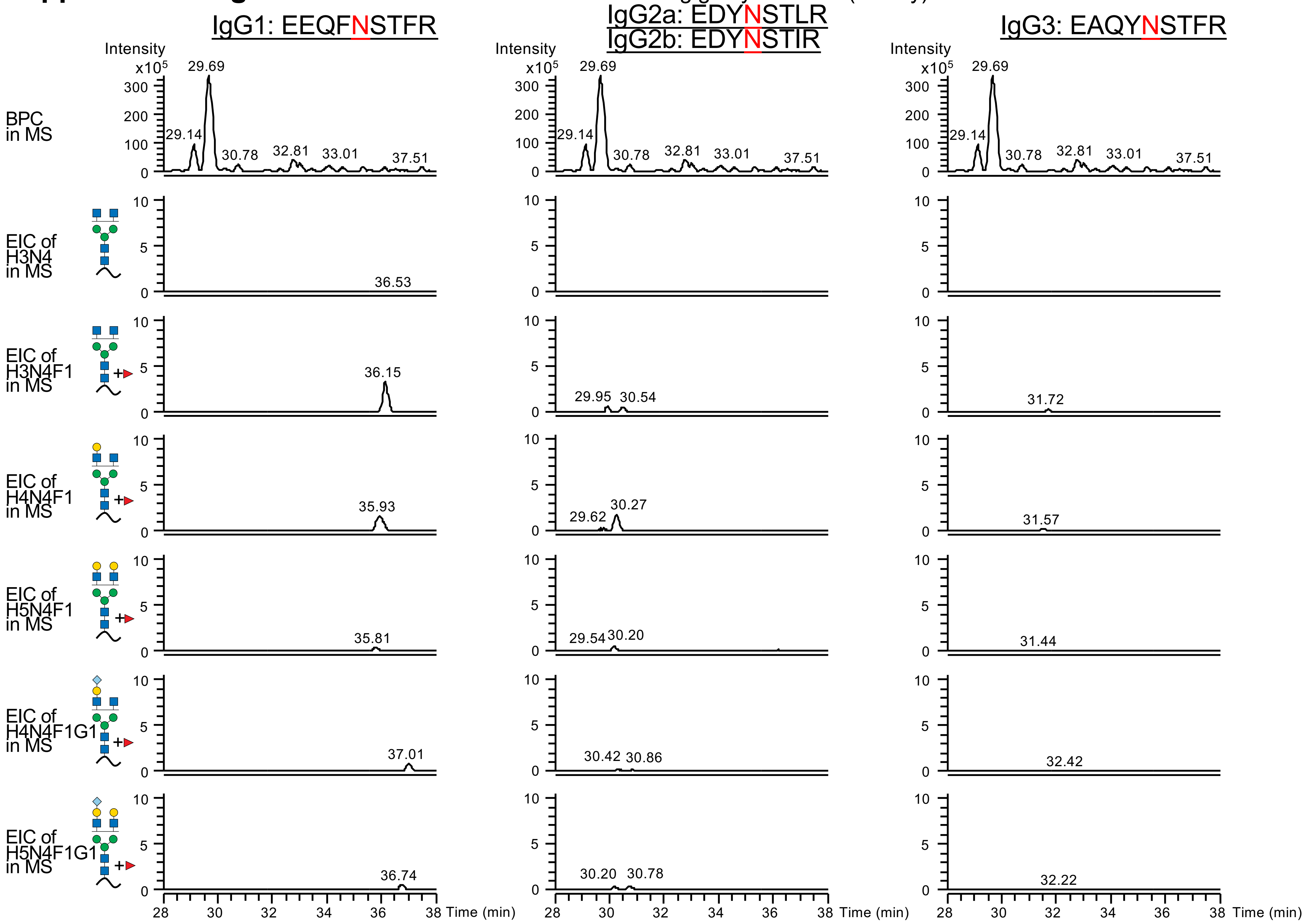

LC-MS analysis of *N*-glycans on IgGs obtained from *Fut8*+/− mice before treatment with 1.2 mg/g/day L-fucose_0th day (C-0).

Supplemental Fig. 9 C-7: treatment with 1.2 mg/g/day L-fucose on the 7th day MS raw data: 21091607

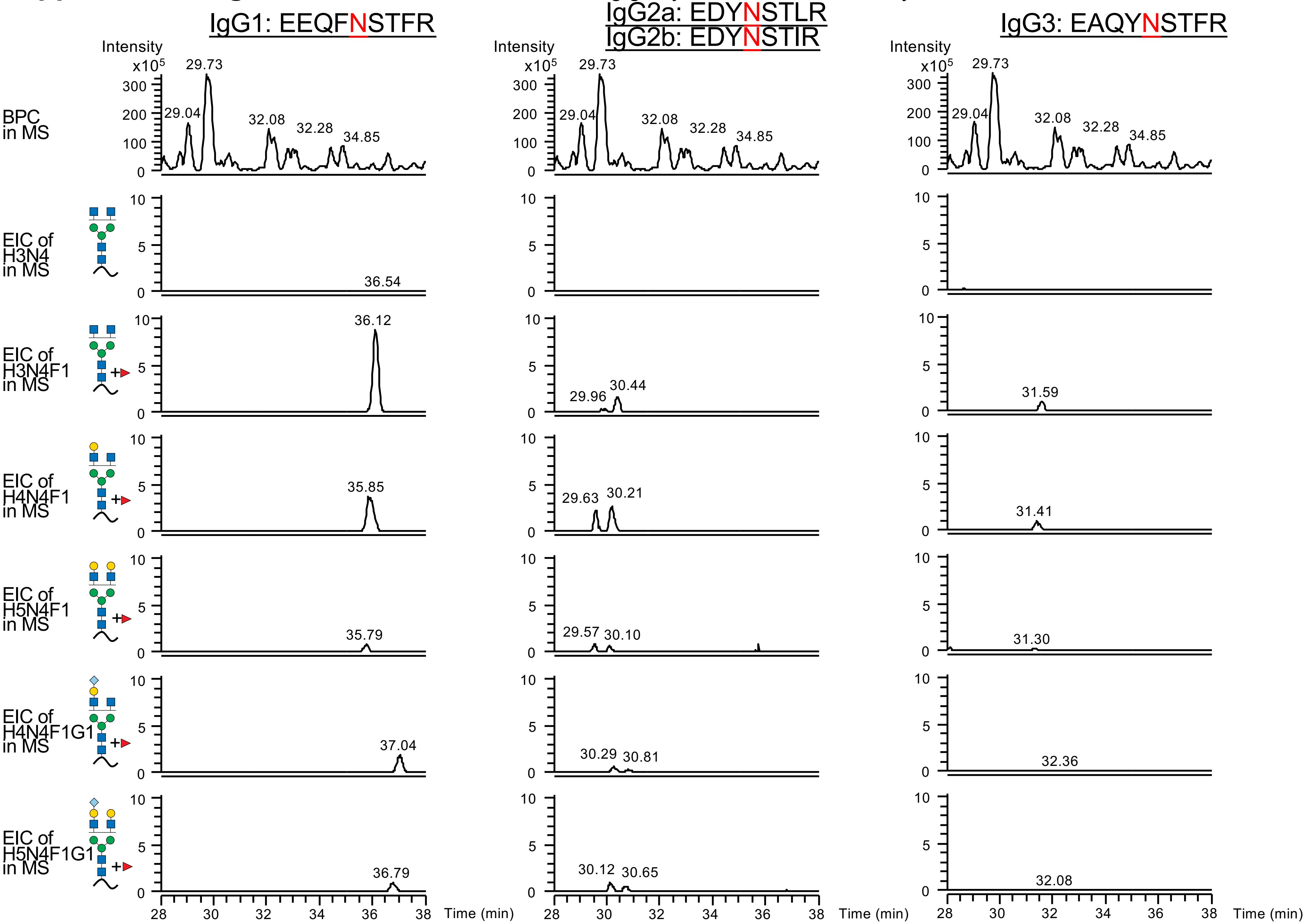

LC-MS analysis of *N*-glycans on IgGs obtained from *Fut8*+/− mice treated with 1.2 mg/g/day L-fucose on the 7th day (C-7).

Supplemental Fig. 10 C-14: treatment with 1.2 mg/g/day L-fucose on the 14th day MS raw data: 21091610

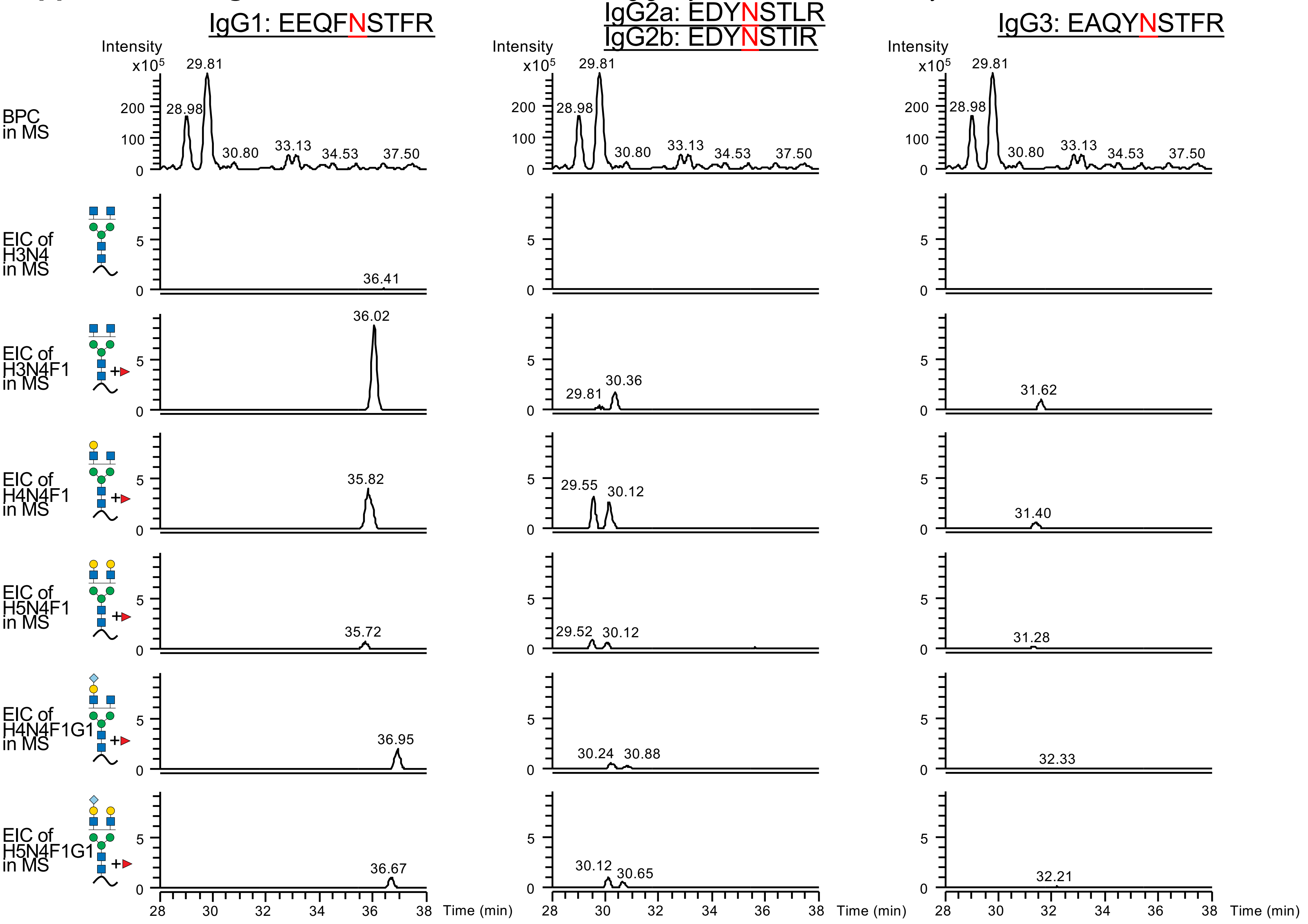

LC-MS analysis of *N*-glycans on IgGs obtained from *Fut8*+/– mice treated with 1.2 mg/g/day L-fucose on the 14th day (C-14).

Supplemental Fig. 11

Identification of the linkage of fucose (Core or Lewis) based on the retention time

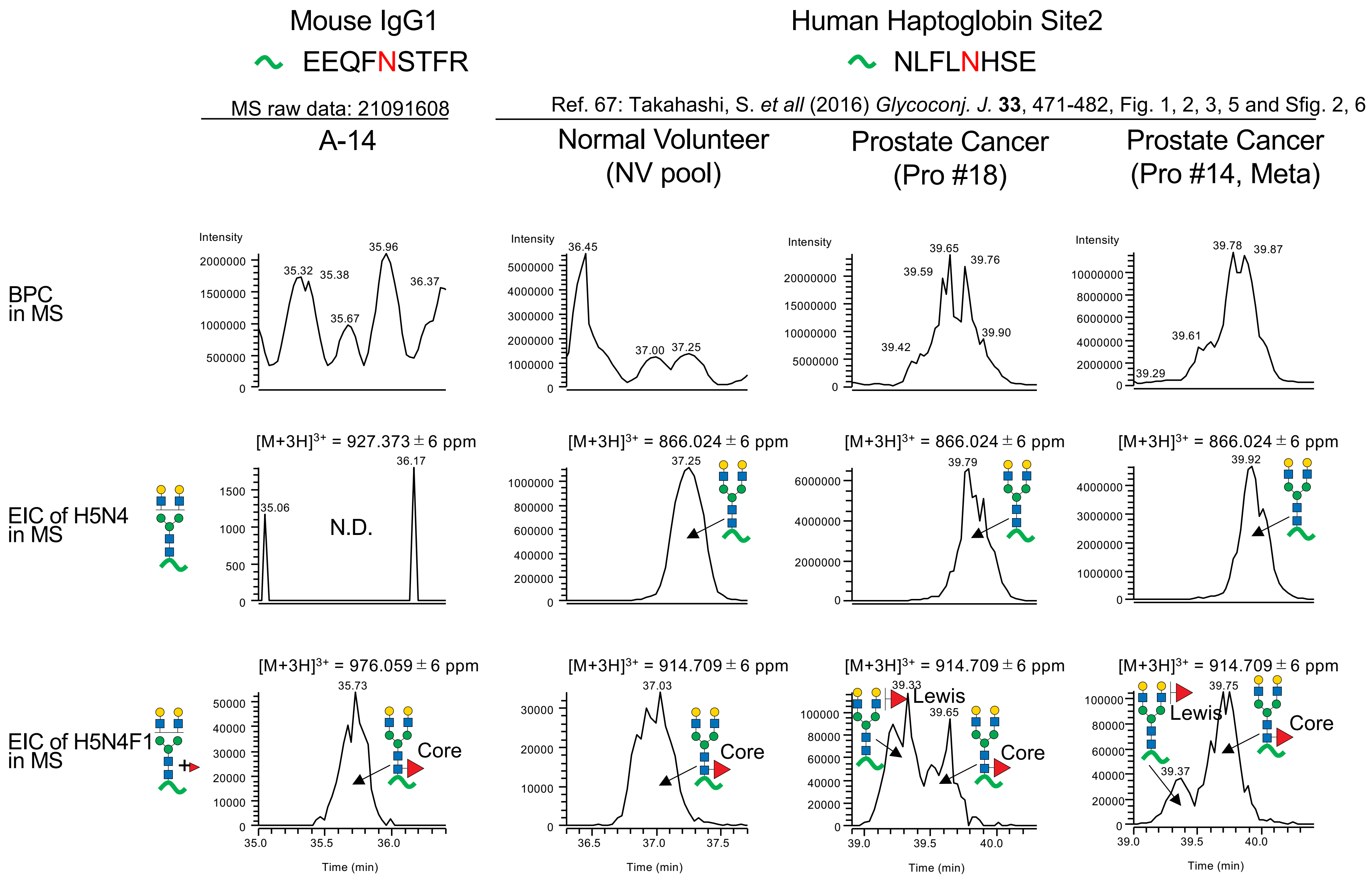

Supplemental Fig. 12

MS raw data: 21091608

Identification of the linkage of fucose (Core or Lewis) based on the retention time

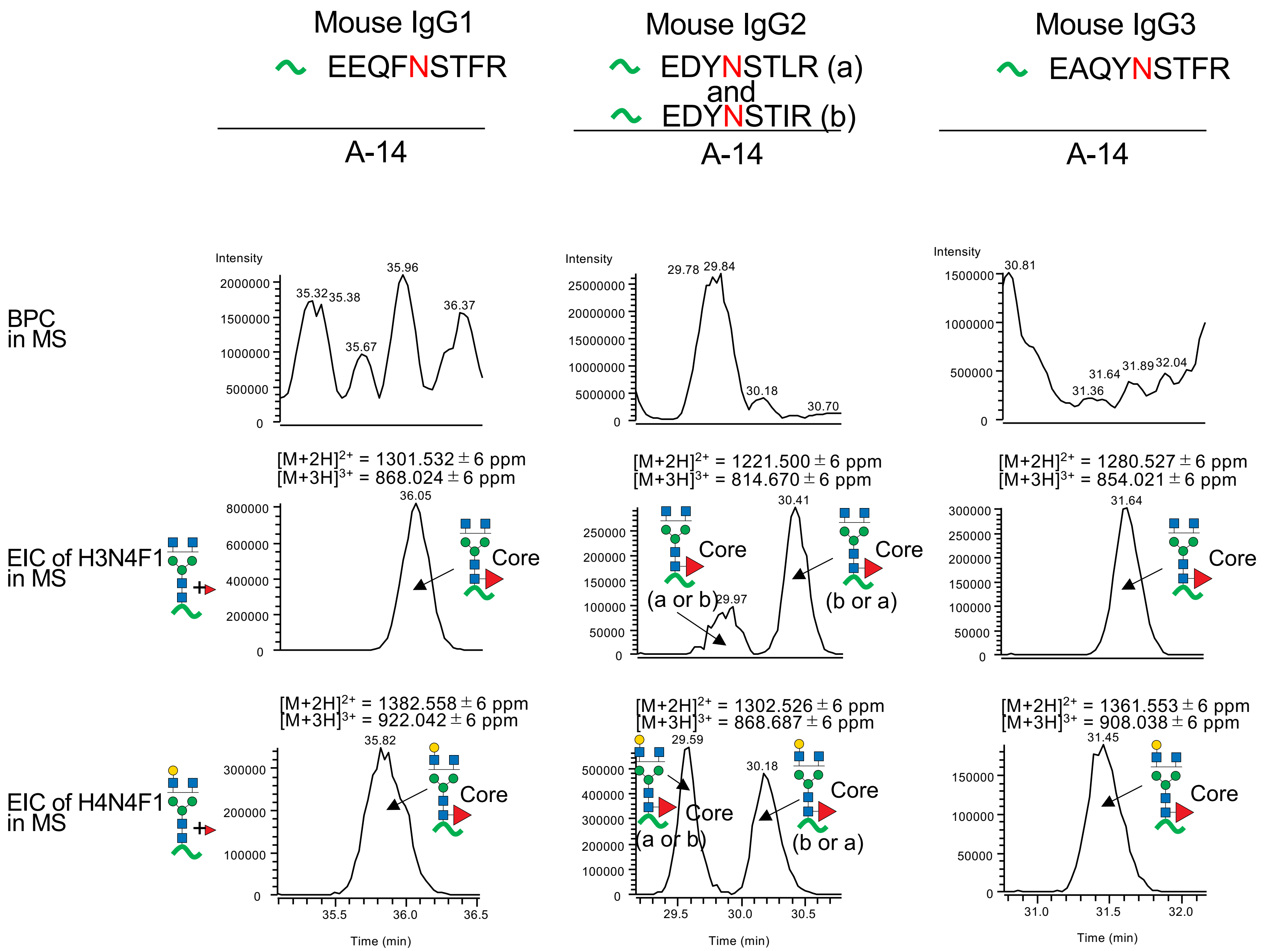

Supplemental Fig. 13

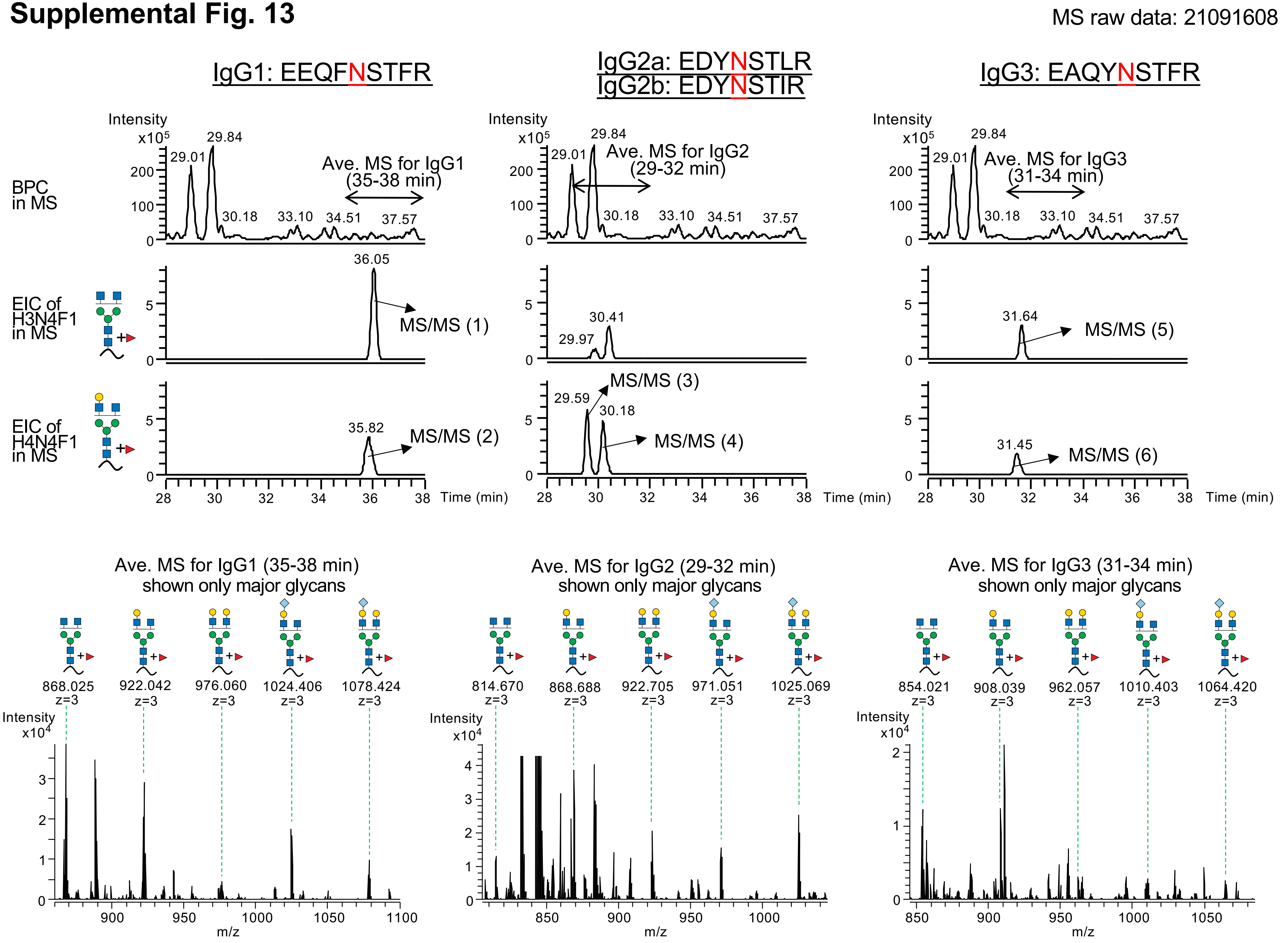

Supplemental Fig. 14

MS raw data: 21091608

A-14 Identification of the linkage of fucose (Core or Lewis) based on MS/MS fragmentation

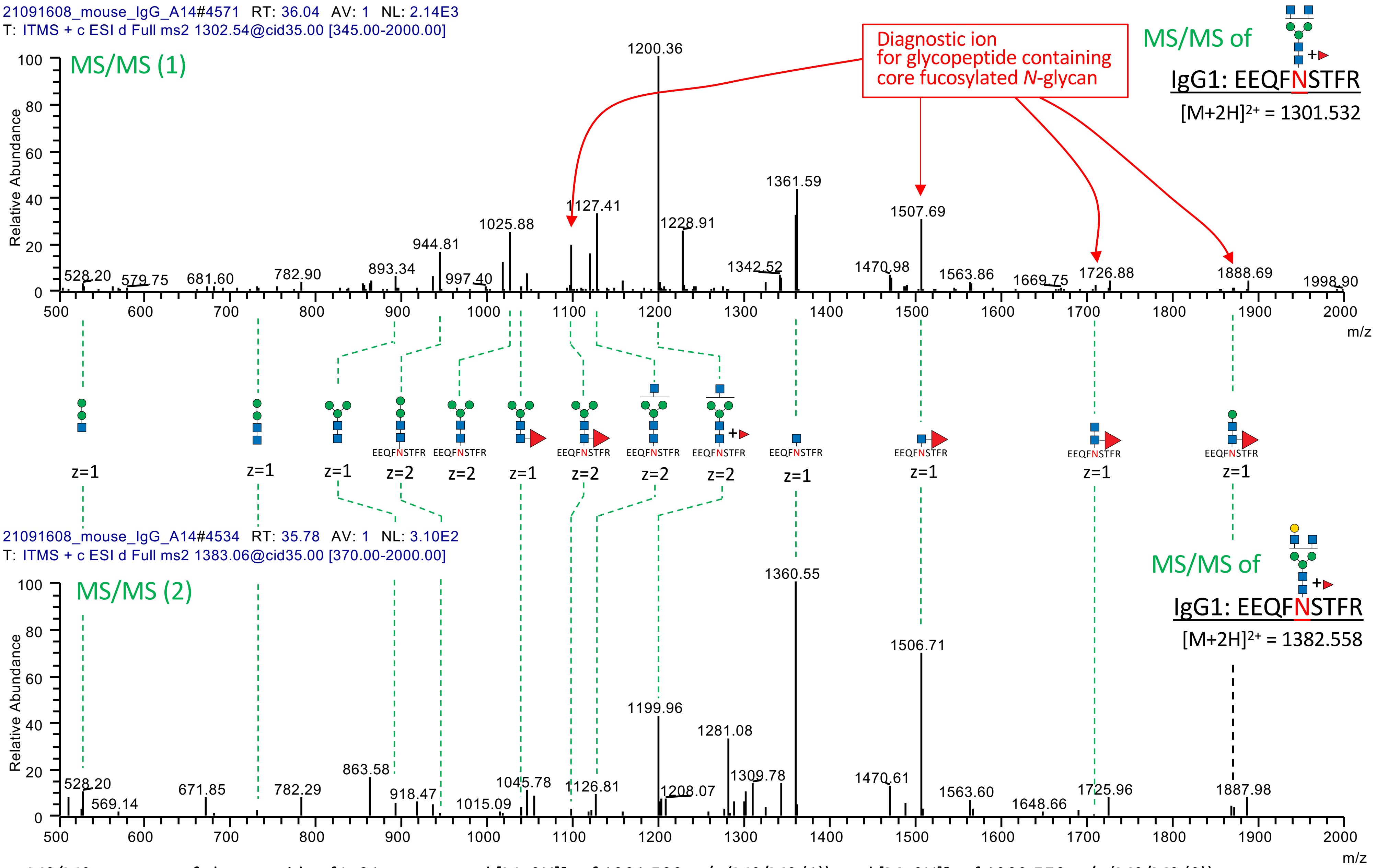

MS/MS spectrum of glycopeptide of IgG1 represented $[M+2H]^{2+}$ of 1301.532 m/z (MS/MS (1)) and $[M+2H]^{2+}$ of 1382.558 m/z (MS/MS (2)). These diagnostic ions revealed that these two glycopeptides contain core fucosylated *N*-glycans.

Supplemental Fig. 15

MS raw data: 21091608

A-14

Identification of the linkage of fucose (Core or Lewis) based on MS/MS fragmentation

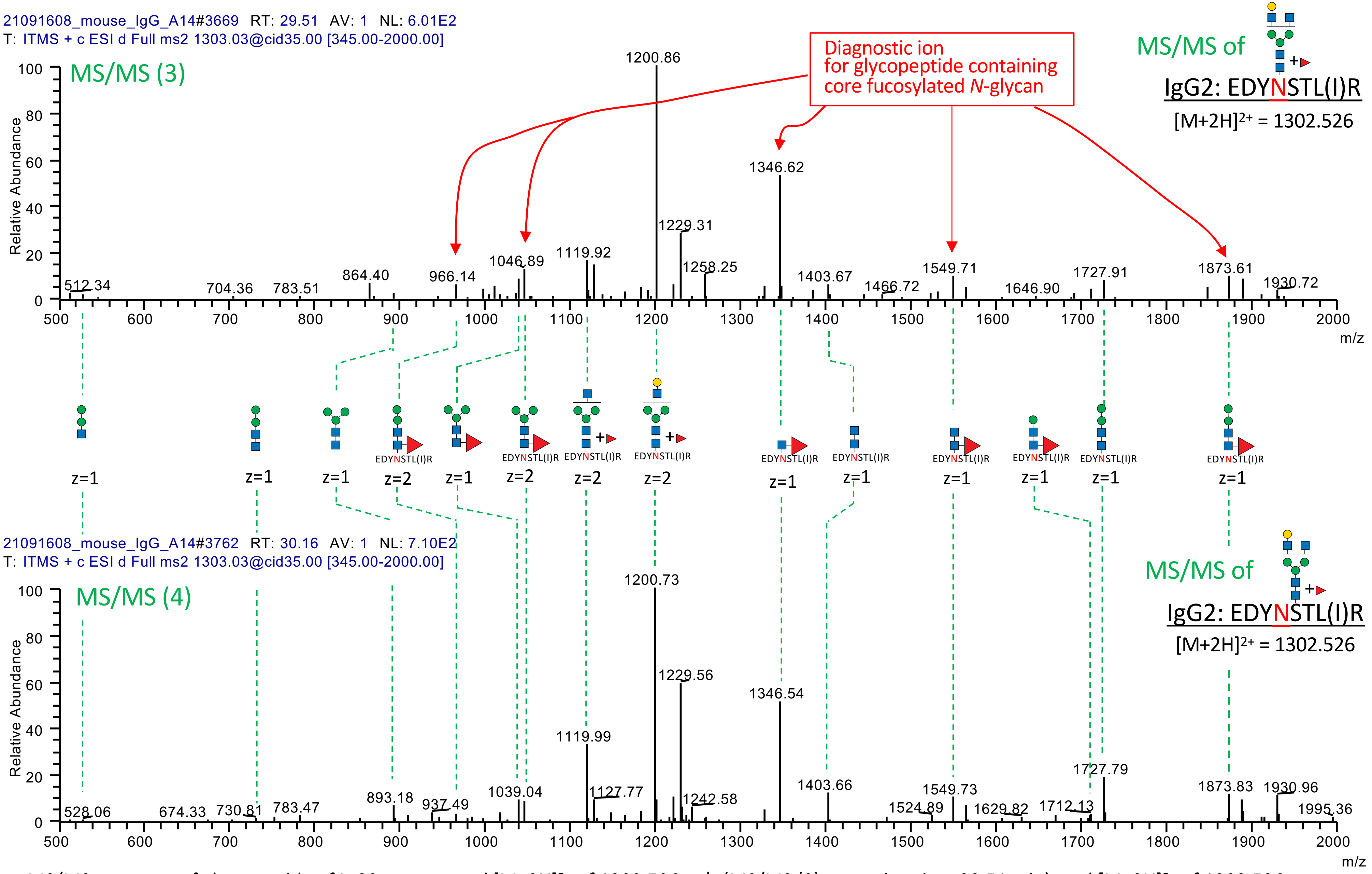

MS/MS spectrum of glycopeptide of IgG2 represented $[M+2H]^{2+}$ of 1302.526 m/z (MS/MS (3), retention time 29.51 min) and $[M+2H]^{2+}$ of 1302.526 m/z (MS/MS (4), retention time 30.16 min). These diagnostic ions revealed that these two glycopeptides contain core fucosylated *N*-glycans.

Supplemental Fig. 16

MS raw data: 21091608

A-14

Identification of the linkage of fucose (Core or Lewis) based on MS/MS fragmentation

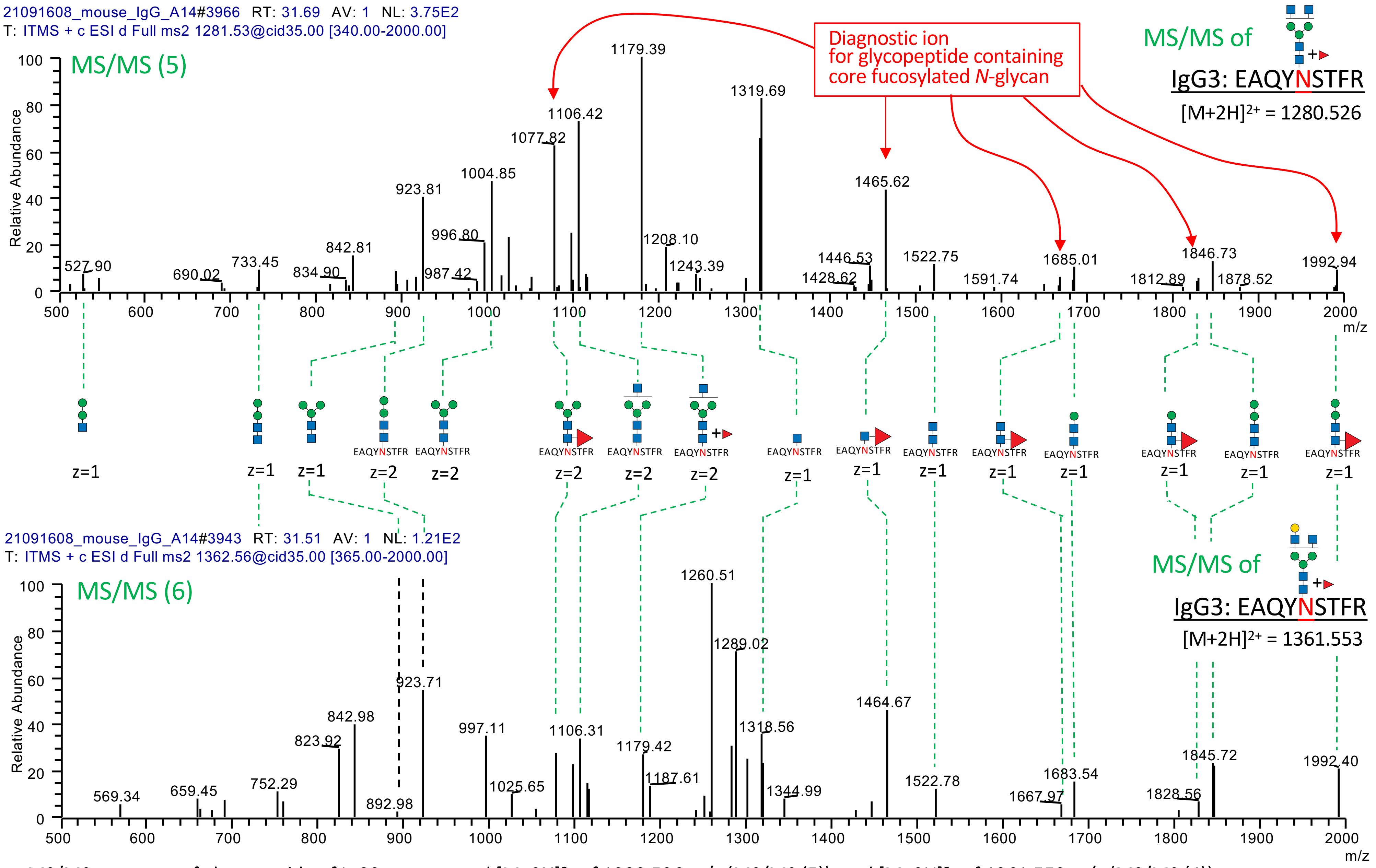

MS/MS spectrum of glycopeptide of IgG3 represented $[M+2H]^{2+}$ of 1280.526 m/z (MS/MS (5)) and $[M+2H]^{2+}$ of 1361.553 m/z (MS/MS (4)).
These diagnostic ions revealed that these two glycopeptides contain core fucosylated *N*-glycans.

Supplemental Fig. 17

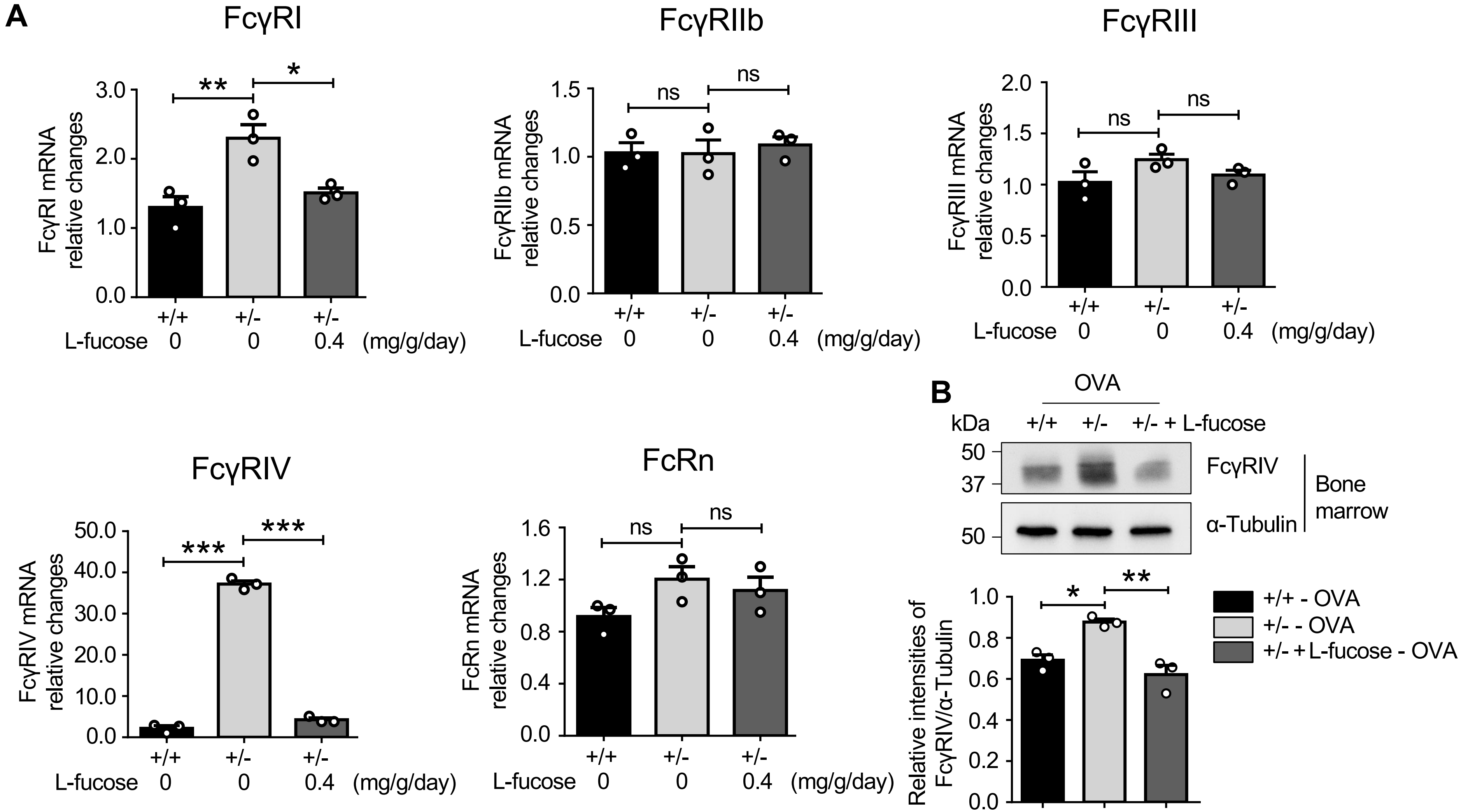

sFig.17. Effects of exogenous L-fucose on FcγRs levels after immunizing with OVA.

(A) After being immunized for the 7th week, the bone marrows were collected, and the mRNA levels of FcγRs in bone marrows were compared among *Fut8*$^{+/+}$, *Fut8*$^{+/-}$, and *Fut8*$^{+/-}$ mice treated with L-fucose at 0.4 mg/g/day. GAPDH was used as an internal control. The data was obtained from three mice, and the data in *Fut8*$^{+/+}$ mice was set as 1.0, which was analyzed by one-way ANOVA with Tukey's *post hoc* analysis as the mean ± SEM. *$p < 0.05$; **$p < 0.01$; ***$p < 0.001$. (B) The expression levels of FcγRIV in the same protein amounts of marrow tissues were detected using Western blot. α-Tubulin was used as a loading control. Data were shown as relative intensities of FcγRIV/α-tubulin, obtained from three mice. The data were qualified by one-way ANOVA with Tukey's *post hoc* analysis as the mean ± SEM. *$p < 0.05$, **$p < 0.01$.

Supplemental Fig. 18

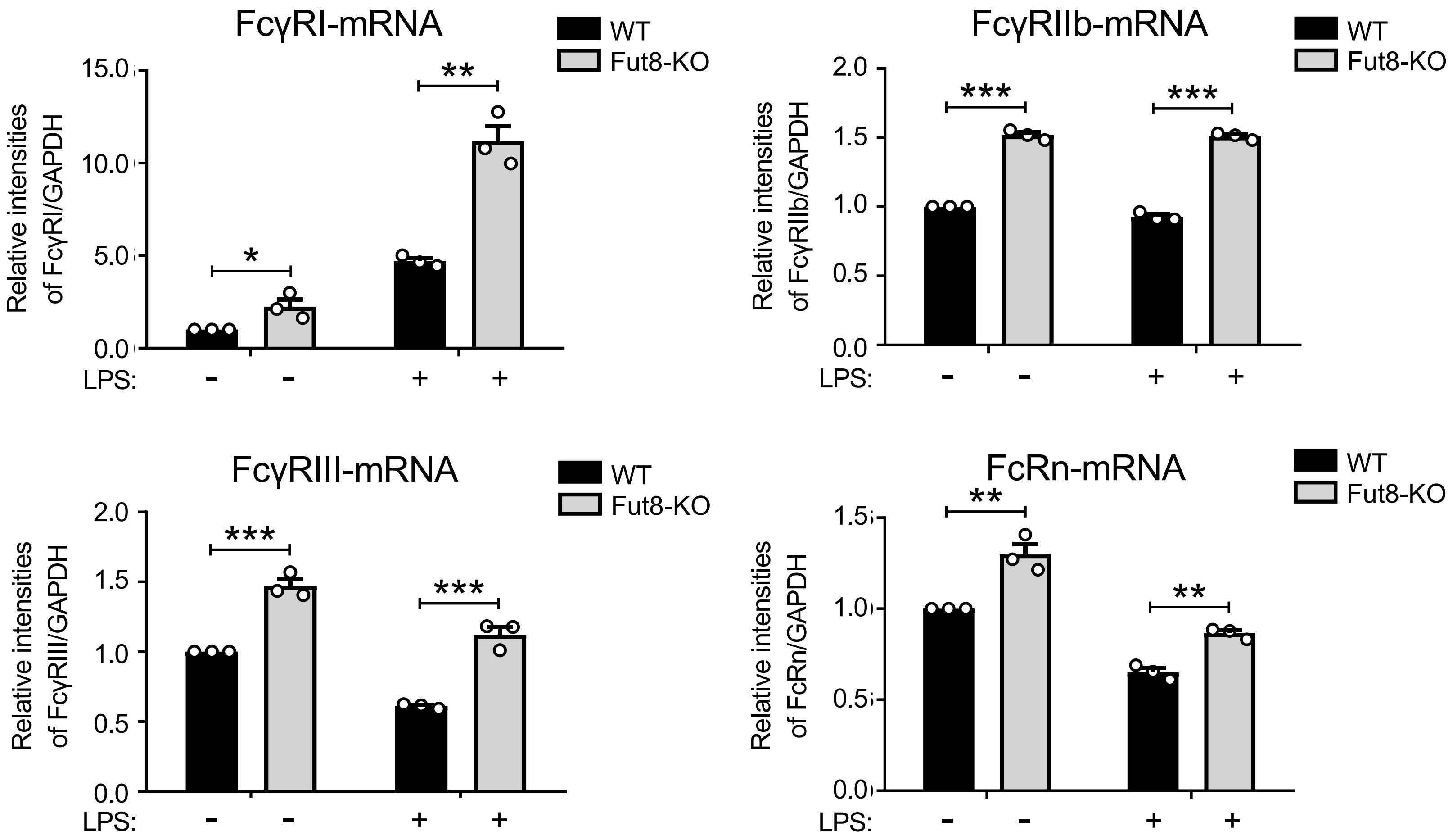

sFig.18. Effects of core fucosylation on the mRNA expression of FcγRs and FcRn.

The WT and Fut8-KO BV2 cells were cultured with or without LPS in a final concentration at 250 ng/ml for 4 h. Real-time PCR was employed to detect the mRNA expression levels of other FcγRs, including FcγRI, FcγRIIb, and FcγRIII, and FcRn. GAPDH was used as an internal control. The value representing relative intensities of FcγRs or FcRn versus GAPDH in WT cells cultured without LPS was set as 1.0. The data were obtained from three independent experiments and subjected to qualification by an unpaired Student *t*-test as the mean ± SEM. *$p < 0.05$, **$p < 0.01$, ***$p < 0.001$.
