# Supplementary material for: Core fucosylation within the Fc-FcγR degradation pathway promotes enhanced IgG levels via exogenous L-fucose
Source: J Biol Chem. 2024 Jul 11;300(8):107558. doi: 10.1016/j.jbc.2024.107558 (PMC11345378; doi:10.1016/j.jbc.2024.107558)

Supplemental Table 1

A-0: before treatment with 0.15 mg/g/day L-fucose (0th day)

MS raw data: 21091602

No.		peptide	Retention time (min)	observed mass			caluculatted mass	throretical mass			△mass	Structure	mass range for EIC (± 6ppm)	Intensity	relative
				M+4H	M+3H	M+2H	M+H	M+3H	M+2H	M+H					
1		EEQFNST FR	36.69			1118.964	2236.920	746.313	1118.966	2236.924	-0.004	H2N3F1	1118.959-1118.973	1176.3	0.10
2			36.43			1199.992	2398.976	800.331	1199.992	2398.977	-0.001	H3N3F1	1199.985-1199.999	13395.4	1.15
3			36.18			1281.021	2561.034	854.349	1281.019	2561.030	0.004	H4N3F1	1281.011-1281.027	3337.6	0.29
4			36.46			1301.534	2602.060	868.024	1301.532	2602.056	0.004	H3N4F1	1301.524-1301.540	94146.7	8.10
			36.46		868.025		2602.059				0.003		868.019-868.029	131993.4	11.36
5			36.21			1382.560	2764.112	922.042	1382.558	2764.109	0.003	H4N4F1	1382.550-1382.566	36295.6	3.12
			36.21		922.043		2764.113				0.004		922.036-922.048	88820.2	7.64
6			36.46		935.718		2805.138	935.717	1403.071	2805.135	0.003	H3N5F1	935.711-935.723	8030.2	0.69
7			37.37		956.713		2868.123	956.712	1434.564	2868.120	0.003	H4N3F1G1	956.706-956.718	1832.2	0.16
8			36.12			1463.588	2926.168	976.059	1463.585	2926.162	0.006	H5N4F1	1463.576-1463.594	5494.9	0.47
			36.12		976.060		2926.164				0.003		976.053-976.065	18563.5	1.60
9			36.21		989.736		2967.192	989.735	1484.098	2967.188	0.004	H4N5F1	989.729-989.741	1555.4	0.13
10			37.15			1536.107	3071.206	1024.405	1536.103	3071.199	0.007	H4N4F1G1	1536.095-1536.113	8918.4	0.77
			37.15		1024.406		3071.202				0.003		1024.399-1024.411	61593	5.30
11			37.03			1617.134	3233.260	1078.423	1617.130	3233.252	0.008	H5N4F1G1	1617.120-1617.140	9514.8	0.82
12			37.12		1092.100		3274.284	1092.098	1637.643	3274.278	0.007	H4N5F1G1	1092.091-1092.105	970.3	0.08
13			36.97		1132.441		3395.307	1132.440	1698.156	3395.305	0.002	H6N4F1G1	1132.433-1132.447	2088.5	0.18
14			37.71			1770.677	3540.346	1180.786	1770.675	3540.342	0.004	H5N4F1G2	1770.664-1770.686	15271.9	1.31
			37.77		1180.787		3540.345				0.003		1180.779-1180.793	1222.2	0.11
15			36.52			1228.504	2456.000	819.338	1228.503	2455.998	0.002	H3N4	1228.496-1228.510	5557.8	0.48
			36.85		819.338		2455.998				0.000		819.333-819.343	7250.2	0.62
16			36.37			1309.534	2618.060	873.356	1309.529	2618.051	0.009	H4N4	1309.521-1309.537	2745.2	0.24
			36.43		873.356		2618.052				0.001		873.351-873.361	2149.8	0.18
17			37.53		975.720		2925.144	975.719	1463.074	2925.141	0.003	H4N4G1	975.713-975.725	2015.3	0.17
18	a	EDYNSTL R	30.95			1119.962	2238.916	746.976	1119.960	2238.913	0.003	H3N3F1	1119.954-1112.968	1855.2	0.16
	b		31.11			1119.961	2238.914				0.001			1910.8	0.16
19			30.71			1200.990	2400.972	800.994	1200.987	2400.966	0.006	H4N3F1	1200.980-1200.994	1835.8	0.16
20	a		30.45			1221.502	2441.996	814.670	1221.500	2441.993	0.003	H3N4F1	1221.493-1221.507	28718.4	2.47
	b		30.93			1221.502	2441.996				0.003			40401.4	3.48
	a		30.45		814.67		2441.994				0.001		814.664-814.674	17007.8	1.46
	b		30.93		814.670		2441.994				0.001			35096.8	3.02
21	a		30.18			1302.528	2604.048	868.687	1302.526	2604.045	0.003	H4N4F1	1302.519-130.535	34176.5	2.94
	b		30.74			1302.529	2604.050				0.005			54564.8	4.69
	a		30.74		868.687		2604.045				0.000		868.682-868.692	35727.2	3.07
	b		30.77		868.687		2604.045				0.000			77318.9	6.65
22			30.61		882.359		2645.061	882.363	1323.040	2645.072	-0.011	H3N5F1	882.357-882.367	1408.7	0.12
23	a		30.12			1383.554	2766.100	922.705	1383.553	2766.098	0.002	H5N4F1	1383.545-1383.561	10473.5	0.90
	b		30.61			1383.554	2766.100				0.002			8950.5	0.77
	a		30.12		922.705		2766.099				0.001		922.699-922.711	10480.2	0.90
	b		30.64		922.705		2766.099				0.001			15168.0	1.30
24	a		30.71			1456.074	2911.140	971.051	1456.072	2911.136	0.004	H4N4F1G1	1456.063-1456.081	7895.5	0.68
	b		31.28			1456.074	2911.140				0.004			7017.8	0.60
	a		30.77		971.051		2911.137				0.001		971.044-971.056	28559.1	2.46
	b		31.28		971.050		2911.134				-0.002			12422.2	1.07
25	a		30.67			1537.099	3073.190	1025.068	1537.098	3073.188	0.002	H5N4F1G1	1537.089-1537.107	9201.7	0.79
	b		31.17			1537.101	3073.194				0.006			5107.7	0.44
	a		30.64		1025.068		3073.188				0.000		1025.062-1025.074	38542.1	3.32
	b		31.17		1025.069		3073.191				0.003			30057.3	2.59
26			30.95		1038.745		3114.219	1038.744	1557.611	3114.215	0.004	H4N5F1G1	1038.738-1038.750	2273.1	0.20
27	a		30.99			1690.644	3380.280	1127.432	1690.643	3380.279	0.001	H5N4F1G2	1690.633-1690.653	2751.7	0.24
	a		31.08		1127.433		3380.283				0.004		1127.424-1127.438	11532.8	0.99
	b		31.49		1127.433		3380.283				0.004			5947.6	0.51
28		EAQYNST FR	32.11			1178.986	2356.964	786.327	1178.987	2356.966	-0.002	H3N3F1	1178.980-1178.994	2047.3	0.18
29			32.05			1280.529	2560.050	854.021	1280.527	2560.046	0.004	H3N4F1	1280.519-1280.535	21012.8	1.81
			32.05		854.021		2560.047				0.001		854.015-854.025	27998.6	2.41
30			31.89			1361.556	2722.104	908.038	1361.553	2722.098	0.006	H4N4F1	1361.545-1361.561	10361.3	0.89
			31.93		908.039		2722.101				0.003		908.033-908.043	21290.7	1.83
31			31.77			1442.584	2884.160	962.056	1442.579	2884.151	0.009	H5N4F1	1442.571-1442.589	2226.9	0.19
			31.80		962.057		2884.155				0.004		962.050-962.062	6087.4	0.52
32			32.75		1010.402		3029.190	1010.402	1515.098	3029.189	0.001	H4N4F1G1	1010.395-1010.407	4199	0.36
33			32.55		1064.420		3191.244	1064.419	1596.125	3191.242	0.002	H5N4F1G1	1064.413-1064.425	5529.7	0.48
34			33.09		1166.786		3498.342	1166.783	1749.670	3498.332	0.010	H5N4F1G2	1166.775-1166.789	1303.9	0.11
													total	1162399.5	100

◇ Neuraminic acid (Neu)
- ◆ *N*-acetyl neuraminic acid (NeuAc)
- ◆ *N*-glycolyl neuraminic acid (NeuGc)

○ Hexose (Hex)
- ● Galactose (Gal)
- ● Mannose (Man)

□ *N*-acetyl hexosamine (HexNAc)
- ■ *N*-acetyl glucosamine (GlcNAc)
- ■ *N*-acetyl galactosamine (GalNAc)

▷ Deoxy hexose (dHex)
- ▶ Fucose (Fuc)

∼ Peptide

LC-MS analysis of *N*-glycans on IgGs obtained from *Fut8*$^{+/-}$ mice before treatment with 0.15 mg/g/day L-fucose_0^{th} day (A-0).

Supplemental Table 2

A-7: treatment with 0.15 mg/g/day L-fucose on the 7th day

MS raw data: 21091605

No.		peptide	Retention time (min)	observed mass M+4H	observed mass M+3H	observed mass M+2H	caluculatted mass M+H	throretical mass M+3H	throretical mass M+2H	throretical mass M+H	△mass	Structure	mass range for EIC (± 6ppm)	Intensity	relative
1		EEQFNSTFR	36.10			1118.971	2236.934	746.313	1118.966	2236.924	0.010	H2N3F1	1118.973-1118.973	3129.2	0.13
			36.23			1199.993	2398.978	800.331	1199.992	2398.977	0.001	H3N3F1	1199.985-1199.999	4511.7	0.18
2			36.31		800.331		2398.977				0.000		800.326-800.336	19375.5	0.79
3			35.93			1281.019	2561.030	854.349	1281.019	2561.03	0.000	H4N3F1	1281.011-1281.027	5014.8	0.20
4			36.20			1301.534	2602.060	868.024	1301.532	2602.056	0.004	H3N4F1	1301.524-1301-540	198760.2	8.08
			36.20		868.025		2602.059				0.003		868.019-868.029	199516.5	8.11
5			35.93			1382.560	2764.112	922.042	1382.558	2764.109	0.003	H4N4F1	1382.550-1382.566	69663.2	2.83
			35.96		922.043		2764.113				0.004		922.036-922.048	101437.2	4.12
6			36.26			1403.074	2805.140	935.717	1403.071	2805.135	0.005	H3N5F1	1403.64-1403.080	5039.6	0.20
			36.17		935.718		2805.138				0.003		935.711-935.723	120011.4	4.88
7			36.02			1463.588	2926.168	976.059	1463.585	2926.162	0.006	H5N4F1	14463.576-1463.594	16463.5	0.67
			35.93		976.060		2926.164				0.002		976.053-976.065	5494.9	0.22
8			35.99		989.738		2967.198	989.735	1484.098	2967.188	0.010	H4N5F1	989.729-989.741	3943.4	0.16
9			37.00			1536.106	3071.204	1024.405	1536.103	3071.199	0.005	H4N4F1G1	1536.095-1536.113	31232.6	1.27
			37.00		1024.406		3071.202				0.003		1024.399-1024.411	77867.2	3.16
10			35.90			1463.588	2926.168	976.059	1463.585	2926.162	0.006	H5N4F1	1463.576-1463.594	9885.4	0.40
11			36.81			1617.131	3233.254	1078.423	1617.130	3233.252	0.002	H5N4F1G1	1617.120-1617.140	7554.9	0.31
			36.78		1078.424		3233.256				0.004		1078.417-1078.429	49825.1	2.02
12			36.97		1092.098		3274.278	1092.098	1637.643	3274.279	-0.001	H4N5F1G1	1092.091-1092.105	2824.9	0.11
13			36.46		1132.438		3395.298	1132.440	1698.156	3395.305	-0.007	H6N4F1G1	1132.433-1132.447	1854.4	0.08
14			37.59			1770.681	3540.354	1180.786	1770.675	3540.342	0.012	H5N4F1G2	1770.664-1770.686	2553.9	0.10
			37.53		1180.787		3540.345				0.003		1180.779-1180.793	21041.1	0.86
15			36.52			1228.505	2456.002	819.338	1228.503	2455.998	0.004	H3N4	1228.496-1228.510	7250.2	0.29
16			36.37			1309.534	2618.060	873.356	1309.529	2618.051	0.009	H4N4	1309.521-1309.537	2745.2	0.11
			36.43		873.356		2618.052				0.001		873.351-873.361	2149.8	0.09
17			37.53		975.720		2925.144	975.719	1463.074	2925.141	0.003	H4N4G1	975.713-975.725	2015.3	0.08
18		EDYNSTLR	30.66			1119.962	2238.916	746.976	1119.960	2238.913	0.003	H3N3F1	1119.954-1119.968	3561.5	0.14
19	a		29.75			1200.989	2400.970	800.994	1200.987	2400.966	0.004	H4N3F1	1200.980-1200.968	4494.1	0.18
	b		30.55			1200.990	2400.972				0.006			2173.4	0.09
20	a		29.97			1221.502	2441.996	814.670	1221.500	2441.993	0.003	H3N4F1	1221.493-1221.507	20743.2	0.84
	b		30.60			1221.502	2441.996				0.003			60698.5	2.47
	a		29.97		814.671		2441.997				0.004		814.664-814.674	13111.7	0.53
	b		30.57		814.670		2441.994				0.001			65069.0	2.64
21	a		29.78			1302.528	2604.048	868.687	1302.526	2604.045	0.003	H4N4F1	1302.519-1302.535	183680.7	7.46
	b		30.35			1302.528	2604.048				0.003			112894	4.59
	a		29.79		868.687		2604.045				0.000		868.682-868.692	145345.6	5.91
	b		30.32		868.688		2604.048				0.003			106375.5	4.32
22			30.29		882.363		2645.073	882.363	1323.040	2645.072	0.001	H3N5F1	882.358-882.368	5913.5	0.24
23			30.66			1354.535	2708.062	903.357	1354.532	2708.056	0.006	H4N3F1G1	1354.524-1354.540	1475.3	0.06
			31.19		903.360		2708.064				0.008		903.352-903.362	1306.7	0.05
24	a		29.78			1383.555	2766.102	922.705	1383.553	2766.098	0.004	H5N4F1	1383.545-1383.561	37207.6	1.51
	b		30.25			1383.554	2766.100				0.002			24186.8	0.98
	a		29.72		922.705		2766.099				0.001		922.699-922.711	47688.9	1.94
	b				922.705		2766.099				0.001			24957.2	1.01
25	a		30.42			1456.074	2911.140	971.051	1456.072	2911.136	0.004	H4N4F1G1	1456.063-1456.081	20015.7	0.81
	b		30.95			1456.074	2911.140				0.004			9150.3	0.37
	a		30.35		971.052		2911.140				0.004		971.044-971.056	47618	1.94
	b		31.01		971.051		2911.137				0.001			20998.8	0.85
26	a		30.25			1537.099	3073.190	1025.068	1537.098	3073.188	0.002	H5N4F1G1	1537.089-1537.107	23204.7	0.94
	b		30.81			1537.101	3073.194				0.006			12838	0.52
	a		30.29		1025.069		3073.191				0.003		1025.062-1025.074	76659.8	3.12
	b		30.84		1025.069		3073.191				0.003			37543.7	1.53
27			30.72			1690.650	3380.292	1127.432	1690.643	3380.279	0.013	H5N4F1G2	1690.633-1690.653	5010.6	0.20
			30.72		1127.433		3380.283				0.004		1127.424-1127.438	28540.8	1.16
28		EAQYNSTFR	31.76			1178.987	2356.966	786.327	1178.987	2356.966	0.000	H3N3F1	1178.981-1178.994	5447.3	0.22
			31.91		786.327		2356.965				-0.001		786.322-786.332	1456.4	0.06
29			31.51			1260.019	2519.030	840.345	1260.013	2519.019	0.011	H4N3F1	1260.005-1260.021	2353.7	0.10
30			31.76			1280.529	2560.050	854.021	1280.527	2560.046	0.004	H3N4F1	1280.519-1280.535	76962	3.13
			31.76		854.021		2560.047				0.001		854.015-854.025	79266.8	3.22
31			31.60			1361.556	2722.104	908.038	1361.553	2722.098	0.006	H4N4F1	1361.545-1361.561	42202.4	1.72
			31.60		908.039		2722.101				0.003		908.033-908.043	67316.2	2.74
32			31.51			1442.583	2884.158	962.056	1442.579	2884.151	0.007	H5N4F1	1442.571-1442.589	8924.7	0.36
			31.51		962.057		2884.155				0.004		962.050-962.062	21703	0.88
33			32.44			1515.101	3029.194	1010.402	1515.098	3029.189	0.005	H4N4F1G1	1515.089-1515.107	5465.8	0.22
			32.44		1010.402		3029.190				0.001		1010.395-1010.407	16763.4	0.68
34			32.29			1596.130	3191.252	1064.419	1596.125	3191.242	0.010	H5N4F1G1	1596.115-1596.135	2839.3	0.12
			32.23		1064.419		3191.241				-0.001		1064.413-1064.425	10966.9	0.45
35			32.98		1166.784		3498.336	1166.783	1749.670	3498.332	0.004	H5N4F1G2	1166.775-1166.789	5351.5	0.22
													total	2460644.1	100

LC-MS analysis of *N*-glycans on IgGs obtained from *Fut8*$^{+/-}$ mice treated with 0.15 mg/g/day L-fucose on the 7th day (A-7).

Supplemental Table 3 A-14: treatment with 0.15 mg/g/day L-fucose on the 14th day

MS raw data: 21091608

No.		peptide	Retention time (min)	observed mass			caluculatted mass	throretical mass			△mass	Structure	mass range for EIC (± 6ppm)	Intensity	relative
				M+4H	M+3H	M+2H	M+H	M+3H	M+2H	M+H					
1		EEQFNSTFR	36.08			1118.968	2236.928	746.313	1118.966	2236.924	0.004	H2N3F1	1118.959-1118.973	5370.6	0.12
2			36.08			1199.994	2398.980	800.331	1199.992	2398.977	0.003	H3N3F1	1199.985-1199.999	29294.3	0.66
			36.25		800.331		2398.977				0.000		800.326-800.336	3238.5	0.07
3			35.90			1281.021	2561.034	854.349	1281.019	2561.030	0.004	H4N3F1	1281.011-1281.027	5818.5	0.13
4			36.05			1301.534	2602.060	868.024	1301.532	2602.056	0.004	H3N4F1	1301.524-1301.540	411288	9.29
			36.05		868.025		2602.059				0.003		868.019-868.029	406701	9.19
5			35.85			1382.560	2764.112	922.042	1382.558	2764.109	0.003	H4N4F1	1382.550-1382.566	140325	3.17
			35.82		922.042		2764.110				0.001		922.036-922.048	212101	4.79
6			35.99			1403.073	2805.138	935.717	1403.071	2805.135	0.003	H3N5F1	1403.064-1403.080	16385.8	0.37
			36.08		935.718		2805.138				0.003		935.711-935.723	34390.9	0.78
7			35.70			1463.586	2926.164	976.059	1463.585	2926.162	0.002	H5N4F1	1463.576-1463.594	21687.7	0.49
			35.73		976.060		2926.164				0.002		976.053-976.065	53573.4	1.21
8			35.85		989.734		2967.186	989.735	1484.098	2967.188	-0.002	H4N5F1	989.729-989.741	7742	0.17
9			36.92			1536.104	3071.200	1024.405	1536.103	3071.199	0.001	H4N4F1G1	1536.095-1536.113	44176.4	1.00
			36.89		1024.406		3071.202				0.003		1024.399-1024.411	164047	3.71
10			36.68			1617.132	3233.256	1078.423	1617.130	3233.252	0.004	H5N4F1G1	1617.120-1617.140	22508.4	0.51
			36.62		1078.424		3233.256				0.004		1078.417-1078.429	82010.8	1.85
11			36.92			1637.645	3274.282	1092.098	1637.643	3274.279	0.003	H4N5F1G1	1637.633-1637.653	2014	0.05
			36.95		1092.100		3274.284				0.005		1092.091-1092.105	4167.5	0.09
12			37.41			1770.675	3540.342	1180.786	1770.675	3540.342	0.000	H5N4F1G2	1770.664-1770.686	4831.2	0.11
			37.46		1180.788		3540.348				0.006		1180.776-1180.793	36164.4	0.82
13			36.42			1228.505	2456.002	819.338	1228.503	2455.998	0.004	H3N4	1228.496-1228.510	10867.2	0.25
			36.39		819.339		2456.001				0.003		819.333-819.343	10508.6	0.24
14			36.14			1309.530	2618.052	873.356	1309.529	2618.051	0.001	H4N4	1309.521-1309.537	4018.9	0.09
			36.17		873.357		2618.055				0.004		873.351-873.361	4084.3	0.09
15		EDYNSTLR	30.47			1119.961	2238.914	746.976	1119.960	2238.913	0.001	H3N3F1	1119.954-1119.968	11607.3	0.26
			30.61		746.976		2238.912				-0.001		746.972-746.980	2219.9	0.05
16			30.15			1200.992	2400.976	800.994	1200.987	2400.966	0.010	H4N3F1	1200.980-1200.994	4862.7	0.11
17	a		29.97			1221.502	2441.996	814.670	1221.500	2441.993	0.003	H3N4F1	1221.493-1221.507	55004.6	1.24
	b		30.41			1221.502	2441.996				0.003			186334	4.21
	a		29.97		814.67		2441.994				0.001		814.664-814.674	44779.7	1.01
	b		30.38		814.670		2441.994				0.001			112349	2.54
18	a		29.56			1302.528	2604.048	868.687	1302.526	2604.045	0.003	H4N4F1	1302.519-1302.535	322589	7.29
	b		30.18			1302.528	2604.048				0.003			249223	5.63
	a		29.59		868.687		2604.045				0.000		868.682-868.692	258274	5.84
	b				868.688		2604.048				0.003			229007	5.18
19	a		30.00			1323.041	2645.074	882.363	1323.040	2645.072	0.002	H3N5F1	1323.032-1323.048	2528.2	0.06
	b		30.06			1323.040	2645.072				0.000			3078.7	0.07
	a		30.09		882.362		2645.070				-0.002		882.357-882.367	5403.3	0.12
	b		30.15		882.363		2645.073				0.001			5913.5	0.13
20			31.08			1354.531	2708.054	903.357	1354.532	2708.056	-0.002	H4N3F1G1	1354.524-1354.540	3171.2	0.07
			31.14		903.357		2708.055				-0.001		903.352-903.362	2194.5	0.05
21	a		29.53			1383.555	2766.102	922.705	1383.553	2766.098	0.004	H5N4F1	1383.545-1383.561	79079.8	1.79
	b		30.12			1383.555	2766.102				0.004			50570.5	1.14
	a		29.50		922.705		2766.099				0.001		922.699-922.711	80177.3	1.81
	b		30.09		922.705		2766.099				0.001			52592.5	1.19
22			29.95		936.381		2807.127	936.380	1404.066	2807.125	0.002	H4N5F1	936.374-936.386	3117.4	0.07
23			30.29		957.372		2870.100	957.375	1435.558	2870.109	-0.009	H5N3F1G1	957.369-957.381	2837.8	0.06
24	a		30.24			1456.073	2911.138	971.051	1456.072	2911.136	0.002	H4N4F1G1	1456.063-1456.081	43446.1	0.98
	b		30.84			1456.074	2911.140				0.004			25337	0.57
	a		30.27		971.052		2911.140				0.004		971.044-971.056	95136.7	2.15
	b		30.90		971.051		2911.137				0.001			43424.5	0.98
25			29.37			1464.577	2928.146	976.722	1464.579	2928.151	-0.005	H6N4F1	1464.570-1461.588	1784.4	0.04
			29.31		976.723		2928.153				0.002		976.716-976.728	3209.2	0.07
26			30.15			1537.102	3073.196	1025.068	1537.098	3073.188	0.008	H5N4F1G1	1537.089-1537.107	47021.8	1.06
27			30.53			1557.618	3114.228	1038.744	1557.611	3114.215	0.013	H4N5F1G1	1557.602-1557.620	3142.6	0.07
28			30.61			1690.642	3380.276	1127.432	1690.643	3380.279	-0.003	H5N4F1G2	1690.633-1690.653	10358	0.23
29		EAQYNSTFR	31.67			1178.989	2356.970	786.327	1178.987	2356.966	0.004	H3N3F1	1178.980-1178.994	2192.9	0.05
			31.79		786.328		2356.968				0.002		786.322-786.332	13471.2	0.30
30			31.48			1260.016	2519.024	840.345	1260.013	2519.019	0.005	H4N3F1	1260.005-1230.021	4439.7	0.10
31			31.64			1280.529	2560.050	854.021	1280.527	2560.046	0.004	H3N4F1	1280.519-1280.535	157569	3.56
			31.61		854.021		2560.047				0.001		854.015-854.025	153216	3.46
32			31.45			1361.555	2722.102	908.038	1361.553	2722.098	0.004	H4N4F1	1361.545-1361.561	109155	2.47
			31.42		908.039		2722.101				0.003		908.033-908.043	79807	1.80
33			31.39			1442.581	2884.154	962.056	1442.579	2884.151	0.003	H5N4F1	1442.571-1442.589	36083.6	0.82
			31.36		962.057		2884.155				0.004		962.050-962.062	17540.9	0.40
34			32.39			1515.104	3029.200	1010.402	1515.098	3029.189	0.011	H4N4F1G1	1515.089-1515.107	28975.5	0.65
			32.36		1010.403		3029.193				0.004		1010.395-1010.407	7252	0.16
35			32.19			1596.130	3191.252	1064.419	1596.125	3191.242	0.010	H5N4F1G1	1596.115-1596.135	23990.1	0.54
			32.19		1064.420		3191.244				0.002		1064.413-1064.425	8224.4	0.19
36			32.88		1166.786		3498.342	1166.783	1749.670	3498.332	0.010	H5N4F1G2	1166.775-1166.789	6059.5	0.14
													total	4425066	100.00

LC-MS analysis of *N*-glycans on IgGs obtained from *Fut8*$^{+/-}$ mice treated with 0.15 mg/g/day L-fucose on the 14th day (A-14).

Supplemental Table 4 B-0: before treatment with 0.4 mg/g/day L-fucose (0th day) MS raw data: 21091603

No.		peptide	Retention time (min)	observed mass			caluculatted mass	throretical mass			△mass	Structure	mass range for EIC (± 6ppm)	Intensity	relative
				M+4H	M+3H	M+2H	M+H	M+3H	M+2H	M+H					
1		EEQFNSTFR	36.25			1199.993	2398.978	800.331	1199.992	2398.977	0.001	H3N3F1	1199.985-1199.999	3150.6	0.19
			36.30		800.33		2398.974				-0.003		800.326-800.336	10969.3	0.66
2			36.04			1281.018	2561.028	854.349	1281.019	2561.030	-0.002	H4N3F1	1281.011-1281.027	3632.4	0.22
3			36.19			1301.534	2602.060	868.024	1301.532	2602.056	0.004	H3N4F1	1301.524-1301.540	109389.4	6.62
			36.16		868.025		2602.059				0.003		868.019-868.029	97861.5	5.93
4			35.98			1382.56	2764.112	922.042	1382.558	2764.109	0.003	H4N4F1	1382.550-1382.566	69817.3	4.23
			35.95		922.042		2764.110				0.001		922.036-922.048	39721.3	2.41
5			36.30		935.72		2805.144	935.717	1403.071	2805.135	0.009	H3N5F1	935.711-935.723	2949.4	0.18
6			35.86			1463.587	2926.166	976.059	1463.585	2926.162	0.004	H5N4F1	1463.576-1463.594	21632	1.31
			35.83		976.06		2926.164				0.002		976.053-976.065	6840.9	0.41
7			36.01		989.738		2967.198	989.735	1484.098	2967.188	0.010	H4N5F1	989.729-989.741	1360.7	0.08
8			36.95			1536.107	3071.206	1024.405	1536.103	3071.199	0.007	H4N4F1G1	1536.095-1536.113	48349.7	2.93
			36.95		1024.406		3071.202				0.003		1024.399-1024.411	7990.2	0.48
9			36.77			1617.135	3233.262	1078.423	1617.130	3233.252	0.010	H5N4F1G1	1617.120-1617.140	29026.7	1.76
			36.71		1078.423		3233.253				0.001		1078.417-1078.429	5825.5	0.35
10			37.55		1180.787		3540.345	1180.786	1770.675	3540.342	0.003	H5N4F1G2	1180.779-1180.793	10414.7	0.63
11			36.62			1228.504	2456.000	819.338	1228.503	2455.998	0.002	H3N4	128.496-1228.510	5779.5	0.35
12			36.28			1309.532	2618.056	873.356	1309.529	2618.051	0.005	H4N4	1309.521-1309.537	2313.9	0.14
			36.39		873.356		2618.052				0.001		873.351-873.361	2354.5	0.14
13			37.38		975.719		2925.141	975.719	1463.074	2925.141	0.000	H4N4G1	975.713-975.725	1339.8	0.08
14	a	EDYNSTLR	30.07			1119.961	2238.914	746.976	1119.960	2238.913	0.001	H3N3F1	1119.954-1119.968	3083.6	0.19
	b		30.62			1119.963	2238.918				0.005			1951	0.12
	a		30.65		746.975		2238.909				-0.004		746.972-746.980	1455.3	0.09
	b		30.77		746.977		2238.915				0.002			5996.8	0.36
15			30.30			1200.990	2400.972	800.994	1200.987	2400.966	0.006	H4N3F1	1200.980-1200.994	1319.6	0.08
			30.56		800.995		2400.969				0.003		800.989-800.999	4016.4	0.24
16	a		29.98			1221.501	2441.994	814.670	1221.500	2441.993	0.001	H3N4F1	1221.493-1221.507	27368.7	1.66
	b		30.56			1221.502	2441.996				0.003			86307.4	5.23
	a		29.98		814.67		2441.994				0.001		814.664-814.674	31505	1.91
	b		30.56		814.67		2441.994				0.001			67404.2	4.08
17	a		29.62			1302.528	2604.048	868.687	1302.526	2604.045	0.003	H4N4F1	1302.519-1302.535	39977.7	2.42
	b		30.33			1302.528	2604.048				0.003			124628.3	7.55
	a		29.73		868.688		2604.048				0.003		868.682-868.692	14949.1	0.91
	b		30.33		868.688		2604.048				0.003			155976.7	9.45
18			31.03		903.358		2708.058	903.380	1354.566	2708.125	-0.067	H4N3F1G1	903.352-903.362	1162.7	0.07
19	a		29.59			1383.555	2766.102	922.705	1383.553	2766.098	0.004	H5N4F1	1383.545-1383.561	10024.8	0.61
	b		30.24			1383.556	2766.104				0.006			22174.5	1.34
	a		29.56		922.705		2766.099				0.001		922.699-922.711	8307.9	0.50
	b		30.17		922.705		2766.099				0.001			26694.3	1.62
20	a		30.39			1456.074	2911.140	971.051	1456.072	2911.136	0.004	H4N4F1G1	1456.063-1456.081	11485	0.70
	b		30.70			1456.074	2911.140				0.004			10063.1	0.61
	a		30.33		971.051		2911.137				0.001		971.044-971.056	31423.1	1.90
	b		30.97		971.051		2911.137				0.001			20923	1.27
21	a		30.24			1537.100	3073.192	1025.068	1537.098	3073.188	0.004	H5N4F1G1	1537.089-1537.107	16135.4	0.98
	b		30.77			1537.1	3073.192				0.004			10719	0.65
	a		30.24		1025.069		3073.191				0.003		1025.062-1025.074	65645.6	3.98
	b		30.80		1025.069		3073.191				0.003			45537.1	2.76
22	a		30.59			1690.648	3380.288	1127.432	1690.643	3380.279	0.009	H5N4F1G2	1690.633-1690.653	2930.3	0.18
	b		31.24			1690.641	3380.274				-0.005			3103.9	0.19
	a		30.65		1127.433		3380.283				0.004		1127.424-1127.438	16771.7	1.02
	b		31.21		1127.432		3380.280				0.001			11104.2	0.67
23		EAQYNSTFR	31.83			1178.989	2356.970	786.327	1178.987	2356.966	0.004	H3N3F1	1178.981-1178.994	1543.2	0.09
			31.89		786.328		2356.968				0.002		786.322-786.332	6371.9	0.39
24			31.58			1260.02	2519.032	840.345	1260.013	2519.019	0.013	H4N3F1	1260.005-1260.021	2374.3	0.14
25			31.76			1280.528	2560.048	854.021	1280.527	2560.046	0.002	H3N4F1	1280.519-1280.535	60041.8	3.64
			31.76		854.021		2560.047				0.001		854.015-854.025	52120.7	3.16
26			31.61			1361.555	2722.102	908.038	1361.553	2722.098	0.004	H4N4F1	1361.545-1361.561	66786.7	4.04
			31.55		908.039		2722.101				0.003		908.033-908.043	34249.1	2.07
27			32.65		942.709		2826.111	942.708	1413.558	2826.109	0.002	H4N3F1G1	942.702-942.714	2165.3	0.13
28			31.48			1442.583	2884.158	962.056	1442.579	2884.151	0.007	H5N4F1	1442.571-1442.589	17440.9	1.06
			31.52		962.056		2884.152				0.001		962.050-962.062	10193	0.62
29			32.44			1515.102	3029.196	1010.402	1515.098	3029.189	0.007	H4N4F1G1	1515.089-1515.107	22260.8	1.35
			32.47		1010.403		3029.193				0.004		1010.395-1010.407	6125.4	0.37
30			32.26			1596.13	3191.252	1064.419	1596.125	3191.242	0.010	H5N4F1G1	1596.115-1596.135	3103	0.19
31			32.18			1207.498	2413.988	805.335	1207.498	2413.988	0.000	H3N4	1207.491-1207.505	1971	0.12
			32.11		805.335		2413.989				0.001		805.329-805.339	2207.7	0.13
32			31.86		859.352		2576.040	859.352	1288.524	2576.041	-0.001	H4N4	859.347-859.357	1575.3	0.10
													total	1651394.8	100

LC-MS analysis of *N*-glycans on IgGs obtained from *Fut8*$^{+/-}$ mice before treatment with 0.4 mg/g/day L-fucose_0th day (B-0).

Supplemental Table 5 B-7: treatment with 0.4 mg/g/day L-fucose on the 7th day MS raw data: 21091606

No.		peptide	Retention time (min)	observed mass M+4H	observed mass M+3H	observed mass M+2H	caluculatted mass M+H	throretical mass M+3H	throretical mass M+2H	throretical mass M+H	△mass	Structure	mass range for EIC (± 6ppm)	Intensity	relative
1		EEQFNSTFR	36.07			1199.993	2398.978	800.331	1199.992	2398.977	0.001	H3N3F1	1199.985-1199.999	12094.9	0.37
			36.24		800.331		2398.977				0.000		800.326-800.336	3898	0.12
2			35.75			1281.019	2561.030	854.349	1281.019	2561.030	0.000	H4N3F1	1281.011-1281.027	5519.9	0.17
3			36.04			1301.533	2602.058	868.024	1301.532	2602.056	0.002	H3N4F1	1301.524-1301.540	188623.7	5.81
			36.07		868.025		2602.059				0.003		868.019-868.029	171123.7	5.27
4			35.78			1382.560	2764.112	922.042	1382.558	2764.109	0.003	H4N4F1	1382.550-1382.566	84264.1	2.60
			35.84		922.043		2764.113				0.004		922.036-922.048	112879.1	3.48
5			36.07		935.720		2805.144	935.717	1403.071	2805.135	0.009	H3N5F1	935.711-935.723	2811	0.09
6			35.72			1463.586	2926.164	976.059	1463.585	2926.162	0.002	H5N4F1	1463.576-1463.594	14657.2	0.45
			35.75		976.060		2926.164				0.002		976.053-976.065	28692.2	0.88
7			36.96			1536.106	3071.204	1024.405	1536.103	3071.199	0.005	H4N4F1G1	1536.095-1536.113	28188.3	0.87
			36.96		1024.406		3071.202				0.003		1024.399-1024.411	82136.3	2.53
8			37.16		1030.072		3088.200	1030.077	1544.611	3088.215	-0.015	H6N4F1	1030.071-1030.083	2777.4	0.09
9			36.63			1617.132	3233.256	1078.423	1617.130	3233.252	0.004	H5N4F1G1	1617.120-1617.140	13644.7	0.42
			36.66		1078.424		3233.256				0.004		1078.417-1078.429	63196.6	1.95
10			37.56		1180.788		3540.348	1180.786	1770.675	3540.342	0.006	H5N4F1G2	1180.779-1180.793	26275.6	0.81
11			36.43			1228.504	2456.000	819.338	1228.503	2455.998	0.002	H3N4	1228.496-1228.510	5582.3	0.17
12			36.32			1309.530	2618.052	873.356	1309.529	2618.051	0.001	H4N4	1309.521-1309.537	5430.4	0.17
			36.12		873.354		2618.046				-0.005		873.351-873.361	2456.4	0.08
13			37.39		975.719		2925.141	975.719	1463.074	2925.141	0.000	H4N4G1	975.713-975.725	2516.4	0.08
14	a	EDYNSTLR	29.92			1119.961	2238.914	746.976	1119.960	2238.913	0.001	H3N3F1	1119.954-1119.968	5156.2	0.16
	b		30.55			1119.963	2238.918				0.005			9782.7	0.30
15			30.18			1200.990	2400.972	800.994	1200.987	2400.966	0.006	H4N3F1	1200.980-1200.994	1553	0.05
			30.44		800.994		2400.966				0.000		800.989-800.999	6491.5	0.20
16	a		29.86			1221.501	2441.994	814.670	1221.500	2441.993	0.001	H3N4F1	1221.493-1221.507	47827.3	1.47
	b		30.38			1221.502	2441.996				0.003			184914.5	5.70
	a		29.80		814.669		2441.991				-0.002		814.664-814.674	49110.4	1.51
	b		30.38		814.670		2441.994				0.001			127808.2	3.94
17	a		29.56			1302.527	2604.046	868.687	1302.526	2604.045	0.001	H4N4F1	1302.519-1302.535	43803.6	1.35
	b		30.14			1302.529	2604.050				0.005			259487.6	7.99
	a		29.56		868.688		2604.048				0.003		868.682-868.692	26838.6	0.83
	b		30.14		868.688		2604.048				0.003			254065	7.82
18			30.72		882.364		2645.076	882.363	1323.040	2645.072	0.004	H3N5F1	882.357-882.367	2268.6	0.07
19			31.15		903.360		2708.064	903.380	1354.566	2708.125	-0.061	H4N3F1G1	903.352-903.362	2132.3	0.07
20	a		29.48			1383.552	2766.096	922.705	1383.553	2766.098	-0.002	H5N4F1	1383.545-1383.561	23857.8	0.73
	b		30.05			1383.552	2766.096				-0.002			39446.8	1.21
	a		29.39		922.705		2766.099				0.001		922.699-922.711	19516.2	0.60
	b		30.05		922.705		2766.099				0.001			45670.3	1.41
21	a		30.21			1456.074	2911.140	971.051	1456.072	2911.136	0.004	H4N4F1G1	1456.063-1456.081	24724.4	0.76
	b		30.77			1456.074	2911.140				0.004			22604.6	0.70
	a		30.21		971.051		2911.137				0.001		971.044-971.056	57719.6	1.78
	b		30.86		971.051		2911.137				0.001			40396.1	1.24
22			29.33		976.722		2928.150	976.722	1464.579	2928.151	-0.001	H6N4F1	976.716-976.728	2224.6	0.07
23	a		30.11			1537.102	3073.196	1025.068	1537.098	3073.188	0.008	H5N4F1G1	1537.602-1537.107	35324.6	1.09
	b		30.66			1537.101	3073.194				0.006			23827.5	0.73
	a		30.08		1025.069		3073.191				0.003		1025.062-1025.074	111328.7	3.43
	b		30.63		1025.069		3073.191				0.003			67805.4	2.09
24			29.92			1618.130	3235.252	1079.086	1618.124	3235.241	0.011	H6N4F1G1	1617.486-1619.860	3670.5	0.11
25	a		30.58			1690.651	3380.294	1127.432	1690.643	3380.279	0.015	H5N4F1G2	1690.633-1690.653	6421.7	0.20
	b		31.18			1690.642	3380.276				-0.003			3009.6	0.09
	a		30.55		1127.433		3380.283				0.004		1127.424-1127.438	32069.8	0.99
	b		31.15		1127.433		3380.283				0.004			18033.4	0.56
26		EAQYNSTFR	31.67			1178.988	2356.968	786.327	1178.987	2356.966	0.002	H3N3F1	1178.980-1178.994	12930.4	0.40
			31.71		786.328		2356.968				0.002		786.322-786.332	4554.6	0.14
27			31.40			1260.018	2519.028	840.345	1260.013	2519.019	0.009	H4N3F1	1260.005-1260.021	7559.2	0.23
			31.49		840.347		2519.025				0.006		840.340-840.350	1566.9	0.05
28			31.65			1280.528	2560.048	854.021	1280.527	2560.046	0.002	H3N4F1	1280.519-1280.535	158008.9	4.87
			31.62		854.021		2560.047				0.001		854.015-854.025	161587.7	4.98
29			31.46			1361.555	2722.102	908.038	1361.553	2722.098	0.004	H4N4F1	1361.545-1361.561	112377.8	3.46
			31.43		908.039		2722.101				0.003		908.033-908.043	134055.9	4.13
30			32.46		942.704		2826.096	942.708	1413.558	2826.109	-0.013	H4N3F1G1	942.702-942.714	1376.2	0.04
31			31.34			1442.582	2884.156	962.056	1442.579	2884.151	0.005	H5N4F1	1442.571-1442.589	23954.6	0.74
			31.34		962.057		2884.155				0.004		962.050-962.062	39923.1	1.23
32			32.28			1515.102	3029.196	1010.402	1515.098	3029.189	0.007	H4N4F1G1	1515.089-1515.107	12538.9	0.39
			32.34		1010.402		3029.190				0.001		1010.395-1010.407	42399.5	1.31
33			32.13			1596.127	3191.246	1064.419	1596.125	3191.242	0.004	H5N4F1G1	1596.115-1596.135	9895.5	0.30
			32.16		1064.420		3191.244				0.002		1064.413-1064.425	40928.7	1.26
34			32.77			1749.671	3498.334	1166.783	1749.670	3498.332	0.002	H5N4F1G2	1749.660-1749.680	4643.5	0.14
			32.83		1166.785		3498.339				0.007		1166.775-1166.789	11413	0.35
35			32.05			1207.500	2413.992	805.335	1207.498	2413.988	0.004	H3N4	1207.491-1207.505	2911.3	0.09
			31.99		805.336		2413.992				0.004		805.329-805.339	3552.6	0.11
36			31.74			1288.527	2576.046	859.352	1288.524	2576.041	0.005	H4N4	1288.516-1288.532	1928.4	0.06
			31.71		859.351		2576.037				-0.004		859.347-859.357	3119.6	0.10
													total	3246885.6	100

LC-MS analysis of *N*-glycans on IgGs obtained from *Fut8*$^{+/-}$ mice treated with 0.4 mg/g/day L-fucose on the 7th day (B-7).

Supplemental Table 6 B-14: treatment with 0.4 mg/g/day L-fucose on the 14th day MS raw data: 21091609

No.		peptide	Retention time (min)	observed mass			caluculatted mass	throretical mass			△mass	Structure	mass range for EIC (± 6ppm)	Intensity	relative
				M+4H	M+3H	M+2H	M+H	M+3H	M+2H	M+H					
1		EEQFNSTFR	36.09			1118.970	2236.932	746.313	1118.966	2236.924	0.008	H2N3F1	1118.959-1118.973	5098.9	0.11
2			36.09			1199.993	2398.978	800.331	1199.992	2398.977	0.001	H3N3F1	1199.985-1199.999	17250.2	0.38
			36.15		800.331		2398.977				0.000		800.326-800.336	3060.8	0.07
3			35.79			1281.018	2561.028	854.349	1281.019	2561.030	-0.002	H4N3F1	1281.011-1281.027	5673.7	0.12
4			36.09			1301.534	2602.060	868.024	1301.532	2602.056	0.004	H3N4F1	1301.524-1301.540	252844	5.54
			36.06		868.025		2602.059				0.003		868.019-868.029	241847	5.30
5			35.79			1382.560	2764.112	922.042	1382.558	2764.109	0.003	H4N4F1	1382.550-1382.566	119347	2.61
			35.85		922.043		2764.113				0.004		922.0366-922.048	139839	3.06
6			36.15		935.715		2805.129	935.717	1403.071	2805.135	-0.006	H3N5F1	935.711-935.723	2741.6	0.06
7			35.64			1463.586	2926.164	976.059	1463.585	2926.162	0.002	H5N4F1	1463.576-1463.594	24612.2	0.54
			35.73		976.060		2926.164				0.002		976.053-976.065	35924.3	0.79
8			36.92			1536.105	3071.202	1024.405	1536.103	3071.199	0.003	H4N4F1G1	1536.095-1536.113	32146.6	0.70
			36.95		1024.406		3071.202				0.003		1024.399-1024.411	113396	2.48
9			37.07		1030.074		3088.206	1030.077	1544.611	3088.215	-0.009	H6N4F1	1030.071-1030.083	3070.7	0.07
10			36.75			1617.134	3233.260	1078.423	1617.130	3233.252	0.008	H5N4F1G1	1617.120-1617.140	19762	0.43
			36.69		1078.424		3233.256				0.004		1078.417-1078.429	71770.3	1.57
11			37.54		1180.788		3540.348	1180.786	1770.675	3540.342	0.006	H5N4F1G2	1180.779-1180.793	34626.6	0.76
12			36.35			1228.504	2456.000	819.338	1228.503	2455.998	0.002	H3N4	1228.496-1228.510	10668.8	0.23
13			36.15			1309.530	2618.052	873.356	1309.529	2618.051	0.001	H4N4	1309.521-1309.537	6465.9	0.14
			36.09		873.356		2618.052				0.001		873.351-873.361	7289.9	0.16
14	a	EDYNSTLR	29.92			1119.960	2238.912	746.976	1119.960	2238.913	-0.001	H3N3F1	1119.954-1119.968	28987.3	0.64
	b		30.45			1119.963	2238.918				0.005			15788.4	0.35
	a		30.50		746.976		2238.912				-0.001		746.972-746.980	4213.3	0.09
15			30.28			1200.990	2400.972	800.994	1200.987	2400.966	0.006	H4N3F1	1200.980-1200.994	7758.6	0.17
16	a		29.87			1221.501	2441.994	814.670	1221.500	2441.993	0.001	H3N4F1	1221.493-1221.507	72722.6	1.59
	b		30.37			1221.502	2441.996				0.003			299884	6.57
	a		29.81		814.669		2441.991				-0.002		814.664-814.674	67337.2	1.48
	b		30.37		814.670		2441.994				0.001			166674	3.65
17	a		29.51			1302.528	2604.048	868.687	1302.526	2604.045	0.003	H4N4F1	1302.519-1302.535	129184	2.83
	b		30.15			1302.528	2604.048				0.003			385042	8.44
	a		29.46		868.687		2604.045				0.000		868.682-868.692	92880.9	2.04
	b		30.12		868.688		2604.048				0.003			331016	7.25
18			30.69			1323.042	2645.076	882.363	1323.040	2645.072	0.004	H3N5F1	1323.032-1323.048	2333.2	0.05
19			31.10			1354.537	2708.066	903.380	1354.566	2708.125	-0.059	H4N3F1G1	1354.524-1354.540	3928.8	0.09
			31.10		903.358		2708.058				-0.067		903.352-903.362	3415.8	0.07
20			30.03		922.705		2766.099	922.705	1383.553	2766.098	0.001	H5N4F1	922.699-922.711	77599.7	1.70
21	a		30.23			1456.075	2911.142	971.051	1456.072	2911.136	0.006	H4N4F1G1	1456.063-1456.081	44562.3	0.98
	b		30.82			1456.073	2911.138				0.002			26353	0.58
	a		30.26		971.052		2911.140				0.004		971.044-971.056	63712.1	1.40
	b		30.90		971.051		2911.137				0.001			49101.7	1.08
22			29.40		976.721		2928.147	976.722	1464.579	2928.151	-0.004	H6N4F1	976.716-976.728	4444.5	0.10
23	a		30.12			1537.102	3073.196	1025.068	1537.098	3073.188	0.008	H5N4F1G1	1537.089-1537.107	59372.2	1.30
	b		30.66			1537.100	3073.192				0.004			46171.5	1.01
	a		30.12		1025.069		3073.191				0.003		1025.062-1025.074	144330	3.16
	b		30.66		1025.069		3073.191				0.003			112133	2.46
24			30.45		1038.745		3114.219	1038.744	1557.611	3114.215	0.004	H4N5F1G1	1038.738-1038.750	3783.3	0.08
25			39.95		1079.085		3235.239	1079.086	1618.124	3235.241	-0.002	H6N4F1G1	1079.080-1079.092	8826.2	0.19
26	a		30.52			1690.649	3380.290	1127.432	1690.643	3380.279	0.011	H5N4F1G2	1690.633-1690.653	11978.1	0.26
	b		31.16			1690.641	3380.274				-0.005			7260.1	0.16
	a		30.58		1127.433		3380.283				0.004		1127.424-1127.438	37694.6	0.83
	b		31.13		1127.434		3380.286				0.007			243806	5.34
27		EAQYNSTFR	31.58			1178.990	2356.972	786.327	1178.987	2356.966	0.006	H3N3F1	1178.980-1178.994	19257.1	0.42
			31.67		786.328		2356.968				0.002		786.322-786.332	2821.6	0.06
28			31.43			1260.016	2519.024	840.345	1260.013	2519.019	0.005	H4N3F1	1260.005-1260.021	9329.7	0.20
29			31.58			1280.529	2560.050	854.021	1280.527	2560.046	0.004	H3N4F1	1280.519-1280.535	202623	4.44
			31.55		854.021		2560.047				0.001		854.015-854.025	160448	3.52
30			31.34			1361.556	2722.104	908.038	1361.553	2722.098	0.006	H4N4F1	1361.545-1361.561	113923	2.50
			31.40		908.039		2722.101				0.003		908.033-908.043	145432	3.19
31			31.34			1442.581	2884.154	962.056	1442.579	2884.151	0.003	H5N4F1	1442.571-1442.589	28572.8	0.63
			31.34		962.057		2884.155				0.004		962.050-962.062	51890.9	1.14
32			32.21			1515.101	3029.194	1010.402	1515.098	3029.189	0.005	H4N4F1G1	1515.089-1515.107	12513.2	0.27
			32.32		1010.402		3029.190				0.001		1010.395-1010.407	50088.9	1.10
33			32.10			1596.129	3191.250	1064.419	1596.125	3191.242	0.008	H5N4F1G1	1596.115-1596.135	10121	0.22
			32.16		1064.421		3191.247				0.005		1064.413-1064.425	33162.2	0.73
34			32.89		1166.784		3498.336	1166.783	1749.670	3498.332	0.004	H5N4F1G2	1166.775-1166.789	15851.4	0.35
35			31.94			1207.499	2413.990	805.335	1207.498	2413.988	0.002	H3N4	1207.491-1207.505	6028.4	0.13
			31.97		805.335		2413.989				0.001		805.329-805.339	2353.6	0.05
													total	4564147	100

LC-MS analysis of *N*-glycans on IgGs obtained from *Fut8*+/− mice treated with 0.4 mg/g/day L-fucose on the 14th day (B-14).

Supplemental Table 7 C-0: before treatment with 1.2 mg/g/day L-fucose (0th day) MS raw data: 21091604

No.		peptide	Retention time (min)	observed mass			caluculatted mass	throretical mass			△mass	Structure	mass range for EIC (± 6ppm)	Intensity	relative
				M+4H	M+3H	M+2H	M+H	M+3H	M+2H	M+H					
1		EEQFNSTFR	36.02			1118.965	2236.922	746.313	1118.966	2236.924	-0.002	H2N3F1	1118.959-1118.973	1822.9	0.13
2			36.15			1199.994	2398.980	800.331	1199.992	2398.977	0.003	H3N3F1	1199.985-1199.999	15689.4	1.11
			36.29		800.331		2398.977				0.000		80.326-800.336	2651.8	0.19
3			35.93			1281.018	2561.028	854.349	1281.019	2561.030	-0.002	H4N3F1	1281.011-1281.027	5050.9	0.36
4			36.15			1301.534	2602.060	868.024	1301.532	2602.056	0.004	H3N4F1	1301.524-1301.540	152752	10.84
			36.15		868.025		2602.059				0.003		868.019-868.029	188721.3	13.39
5			35.96			1382.560	2764.112	922.042	1382.558	2764.109	0.003	H4N4F1	1382.550-1382.566	60509	4.29
			35.93		922.042		2764.110				0.001		922.036-922.048	109802.2	7.79
6			36.12			1403.071	2805.134	935.717	1403.071	2805.135	-0.001	H3N5F1	1403.064-1403.080	2846.9	0.20
			36.12		935.718		2805.138				0.003		935.711-935.723	12206.3	0.87
7			37.17		956.711		2868.117	956.712	1434.564	2868.120	-0.003	H4N3F1G1	956.706-956.718	1148.5	0.08
8			35.81			1463.585	2926.162	976.059	1463.585	2926.162	0.000	H5N4F1	1463.576-1463.594	11578.7	0.82
			35.84		976.060		2926.164				0.002		976.053-976.065	34199.1	2.43
9			36.98			1536.105	3071.202	1024.405	1536.103	3071.199	0.003	H4N4F1G1	1536.095-1536.113	14932.2	1.06
			37.01		1024.406		3071.202				0.003		1024.399-1024.411	70068.2	4.97
10			36.77			1617.133	3233.258	1078.423	1617.130	3233.252	0.006	H5N4F1G1	1617.120-1617.140	8465.8	0.60
			36.74		1078.424		3233.256				0.004		1078.417-1078.429	49376.7	3.50
11			37.11		1092.100		3274.284	1092.098	1637.643	3274.279	0.005	H4N5F1G1	1092.091-1092.105	1419	0.10
12			36.41		1132.441		3395.307	1132.440	1698.156	3395.305	0.002	H6N4F1G1	1132.433-1132.447	3552.1	0.25
13			37.60			1770.676	3540.344	1180.786	1770.675	3540.342	0.002	H5N4F1G2	1770.664-1770.686	2132.6	0.15
			37.60		1180.788		3540.348				0.006		1180.779-1180.793	19728.4	1.40
14			36.53			1228.506	2456.004	819.338	1228.503	2455.998	0.006	H3N4	1228.496-1228.510	4105	0.29
15			36.26		873.355		2618.049	873.356	1309.529	2618.051	-0.002	H4N4	873.351-873.361	2806.2	0.20
16	a	EDYNSTLR	29.95			1119.964	2238.920	746.976	1119.960	2238.913	0.007	H3N3F1	1119.954-1119.968	2300.5	0.16
	b		30.51			1119.963	2238.918				0.005			3181.8	0.23
17			30.20			1200.987	2400.966	800.994	1200.987	2400.966	0.000	H4N3F1	1200.980-1200.994	3417.8	0.24
18	a		29.95			1221.502	2441.996	814.670	1221.500	2441.993	0.003	H3N4F1	1221.493-1221.507	35456.5	2.52
	b		30.48			1221.502	2441.996				0.003			32069.7	2.27
	a		29.95		814.669		2441.991				-0.002		814.664-814.674	34834.7	2.47
	b		30.54		814.670		2441.994				0.001			30217.6	2.14
19	a		29.62			1302.532	2604.056	868.687	1302.526	2604.045	0.011	H4N4F1	1302.519-1302.535	17885	1.27
	b		30.27			1302.528	2604.048				0.003			79644.6	5.65
	a		30.27		868.688		2604.048				0.003		868.682-868.692	94784.5	6.72
20			30.17			1323.042	2645.076	882.363	1323.040	2645.072	0.004	H3N5F1	1323.032-1323.048	1578.2	0.11
21			30.20			1383.554	2766.100	922.705	1383.553	2766.098	0.002	H5N4F1	1383.545-1383.561	24422.8	1.73
			30.17		922.706		2766.102				0.004		922.699-922.711	31530.5	2.24
22			30.04		936.380		2807.124	936.380	1404.066	2807.125	-0.001	H4N5F1	936.374-936.386	2171	0.15
23	a		30.30			1456.073	2911.138	971.051	1456.072	2911.136	0.002	H4N4F1G1	1456.063-1456.081	7282.5	0.52
	b		30.83			1456.073	2911.138				0.002			5949.7	0.42
	a		30.42		971.051		2911.137				0.001		971.044-971.056	18045	1.28
	b		30.86		971.051		2911.137				0.001			15397.5	1.09
24			29.42		976.723		2928.153	976.722	1464.579	2928.151	0.002	H6N4F1	976.716-976.728	1194.4	0.08
25	a		30.20			1537.102	3073.196	1025.068	1537.098	3073.188	0.008	H5N4F1G1	1537.089-1537.107	7469.7	0.53
	b		30.78			1537.100	3073.192				0.004			10291.9	0.73
	a		30.20		1025.069		3073.191				0.003		1025.062-1025.074	32189.5	2.28
	b		30.78		1025.068		3073.188				0.000			33342.7	2.37
26			29.98		1079.085		3235.239	1079.086	1618.124	3235.241	-0.002	H6N4F1G1	1079.080-1079.092	2131.6	0.15
27	b		31.16			1690.642	3380.276	1127.432	1690.643	3380.279	-0.003	H5N4F1G2	1690.633-1690.653	1247.3	0.09
	a		30.57		1127.433		3380.283				0.004		1127.424-1127.438	7387.9	0.52
	b		31.19		1127.433		3380.283				0.004			7896.7	0.56
28		EAQYNSTFR	31.72			1178.990	2356.972	786.327	1178.987	2356.966	0.006	H3N3F1	1178.980-1178.994	1538.5	0.11
29			31.72			1280.528	2560.048	854.021	1280.527	2560.046	0.002	H3N4F1	1280.519-1280.535	16371.1	1.16
			31.69		854.021		2560.047				0.001		854.015-854.025	17934.7	1.27
30			31.57			1361.555	2722.102	908.038	1361.553	2722.098	0.004	H4N4F1	1361.545-1361.561	13314.6	0.94
			31.50		908.038		2722.098				0.000		908.033-908.043	17113.8	1.21
31			31.41			1442.581	2884.154	962.056	1442.579	2884.151	0.003	H5N4F1	1442.571-1442.589	3148.1	0.22
			31.44		962.056		2884.152				0.001		962.050-962.062	6937.8	0.49
32			32.42			1515.100	3029.192	1010.402	1515.098	3029.189	0.003	H4N4F1G1	1515.089-1515.107	1332.7	0.09
			32.26		1010.403		3029.193				0.004		1010.395-1010.407	4572	0.32
33			32.22			1596.127	3191.246	1064.419	1596.125	3191.242	0.004	H5N4F1G1	1596.115-1596.135	1262.1	0.09
			32.22		1064.422		3191.250				0.008		1064.413-1064.425	5380.4	0.38
34			32.90		1166.782		3498.330	1166.783	1749.670	3498.332	-0.002	H5N4F1G2	1166.775-1166.789	1921.2	0.14
													total	1409713.8	100

LC-MS analysis of *N*-glycans on IgGs obtained from *Fut8*$^{+/-}$ mice before treatment with 1.2 mg/g/day L-fucose_0th day (C-0).

Supplemental Table 8 C-7: treatment with 1.2 mg/g/day L-fucose on the 7th day

MS raw data: 21091607

No.		peptide	Retention time (min)	observed mass M+4H	observed mass M+3H	observed mass M+2H	caluculatted mass M+H	throretical mass M+3H	throretical mass M+2H	throretical mass M+H	△mass	Structure	mass range for EIC (± 6ppm)	Intensity	relative
1		EEQFNSTFR	36.15			1118.966	2236.924	746.313	1118.966	2236.924	0.000	H2N3F1	1118.959-1118.973	6134.2	0.18
2			36.09			1199.992	2398.976	800.331	1199.992	2398.977	-0.001	H3N3F1	1199.985-1199.999	26640.9	0.80
			36.27		800.331		2398.977				0.000		800.326-800.336	2875.3	0.09
3			35.90			1281.019	2561.030	854.349	1281.019	2561.030	0.000	H4N3F1	1281.011-1281.027	8871.9	0.27
4			36.12			1301.534	2602.060	868.024	1301.532	2602.056	0.004	H3N4F1	1301.524-1301.540	467532.9	14.08
			36.12		868.025		2602.059				0.003		868.019-868.029	415323.4	12.51
6			36.01			1382.560	2764.112	922.042	1382.558	2764.109	0.003	H4N4F1	1382.550-1382.566	161489.4	4.86
			35.85		922.042		2764.110				0.001		922.036-922.048	215627.8	6.49
7			36.15			1403.073	2805.138	935.717	1403.071	2805.135	0.003	H3N5F1	1403.064-1403.080	6686.1	0.20
			36.15		935.718		2805.138				0.003		935.711-935.723	8841.4	0.27
9			35.73			1463.586	2926.164	976.059	1463.585	2926.162	0.002	H5N4F1	1463.576-1463.594	30056.3	0.91
			35.79		976.059		2926.161				-0.001		976.053-976.065	56050.4	1.69
12			37.04			1536.105	3071.202	1024.405	1536.103	3071.199	0.003	H4N4F1G1	1536.095-1536.113	53135.9	1.60
			37.04		1024.406		3071.202				0.003		1024.399-1024.411	138115	4.16
17			36.79			1617.131	3233.254	1078.423	1617.130	3233.252	0.002	H5N4F1G1	1617.120-1617.140	23973.4	0.72
			36.79		1078.424		3233.256				0.004		1078.417-1078.429	78518.2	2.37
18			37.04		1092.095		3274.269	1092.098	1637.643	3274.279	-0.010	H4N5F1G1	1092.091-1092.105	2496	0.08
19			36.41		1132.442		3395.310	1132.440	1698.156	3395.305	0.005	H6N4F1G1	1132.433-1132.447	3268.1	0.10
22			37.59			1770.681	3540.354	1180.786	1770.675	3540.342	0.012	H5N4F1G2	1770.664-1770.686	8494	0.26
			37.64		1180.788		3540.348				0.006		1180.779-1180.793	41487.6	1.25
23			36.54			1228.508	2456.008	819.338	1228.503	2455.998	0.010	H3N4	1228.496-1228.510	7075.1	0.21
			36.49		819.339		2456.001				0.003		819.333-819.343	5609.9	0.17
24			36.15			1309.531	2618.054	873.356	1309.529	2618.051	0.003	H4N4	1309.521-1309.537	2771.3	0.08
			36.27		873.356		2618.052				0.001		873.351-873.361	4098.9	0.12
24		EDYNSTLR	30.47			1119.962	2238.916	746.976	1119.960	2238.913	0.003	H3N3F1	1119.954-1119.968	6347.6	0.19
25			30.26			1200.989	2400.970	800.994	1200.987	2400.966	0.004	H4N3F1	1200.980-1200.994	3802	0.11
26	a		29.96			1221.500	2441.992	814.670	1221.500	2441.993	-0.001	H3N4F1	1221.493-1221.507	32942	0.99
	b		30.44			1221.502	2441.996				0.003			101725.7	3.06
	a		29.93		814.668		2441.988				-0.005		814.664-814.674	18503.8	0.56
	b		30.37		814.669		2441.991				-0.002			67644.1	2.04
28	a		29.57			1302.528	2604.048	868.687	1302.526	2604.045	0.003	H4N4F1	1302.519-1302.535	146085.7	4.40
	b		30.21			1302.528	2604.048				0.003			155830.6	4.69
	a		29.63		868.687		2604.045				0.000		868.682-868.692	102496.7	3.09
	b		30.21		868.687		2604.045				0.000			107958.4	3.25
33			31.01			1354.534	2708.060	903.357	1354.532	2708.056	0.004	H4N3F1G1	1354.524-1354.540	2702.3	0.08
			31.04		903.357		2708.055				-0.001		903.352-903.362	1251.8	0.04
31	a		29.54			1383.555	2766.102	922.705	1383.553	2766.098	0.004	H5N4F1	1383.545-1383.561	42022.6	1.27
	b		30.07			1383.556	2766.104				0.006			32490	0.98
	a		29.57		922.705		2766.099				0.001		922.699-922.711	45131.4	1.36
	b		30.10		922.705		2766.099				0.001			43696.6	1.32
34	a		30.29			1456.074	2911.140	971.051	1456.072	2911.136	0.004	H4N4F1G1	1456.063-1456.081	28610.6	0.86
	b		30.81			1456.075	2911.142				0.006			11914.4	0.36
	a		30.29		971.051		2911.137				0.001		971.044-971.056	39856.7	1.20
	b		30.81		971.052		2911.140				0.004			21018.6	0.63
39	a		30.12			1537.100	3073.192	1025.068	1537.098	3073.188	0.004	H5N4F1G1	1537.089-1537.107	31407.5	0.95
	b		30.65			1537.099	3073.190				0.002			20226.4	0.61
	a		30.12		1025.069		3073.191				0.003		1025.062-1025.074	70028.9	2.11
	b		30.68		1025.069		3073.191				0.003			38614.7	1.16
41			30.47		1079.087		3235.245	1079.086	1618.124	3235.241	0.004	H6N4F1G1	1079.080-1079.092	2894.2	0.09
44	a		30.63			1690.643	3380.278	1127.432	1690.643	3380.279	-0.001	H5N4F1G2	1690.633-1690.653	5842.3	0.18
	b		31.15			1690.649	3380.290				0.011			2239.1	0.07
	a		30.63		1127.434		3380.286				0.007		1127.424-1127.438	24749.8	0.75
	b		31.13		1127.433		3380.283				0.004			14461.2	0.44
45			36.62			1310.528	2620.048	874.019	1310.524	2620.040	0.008	H5N4	1310.516-1310.532	15792.4	0.48
			36.59		874.020		2620.044				0.004		874.014-874.024	33541.1	1.01
46		EAQYNSTFR	31.62			1178.989	2356.970	786.327	1178.987	2356.966	0.004	H3N3F1	1178.980-1178.994	4832.8	0.15
48			31.53			1280.528	2560.048	854.021	1280.527	2560.046	0.002	H3N4F1	1280.519-1280.535	60359.8	1.82
			31.59		854.021		2560.047				0.001		854.015-854.025	55888.3	1.68
50			31.41			1361.555	2722.102	908.038	1361.553	2722.098	0.004	H4N4F1	1361.545-1361.561	41967.6	1.26
			31.41		908.039		2722.101				0.003		908.033-908.043	52097.6	1.57
53			31.35			1442.581	2884.154	962.056	1442.579	2884.151	0.003	H5N4F1	1442.571-1442.589	10538	0.32
			31.30		962.057		2884.155				0.004		962.050-962.062	19753.7	0.59
56			32.36			1515.103	3029.198	1010.402	1515.098	3029.189	0.009	H4N4F1G1	1515.089-1515.107	5420.8	0.16
			32.33		1010.404		3029.196				0.007		1010.395-1010.407	11586.1	0.35
61			32.08		1064.423		3191.253	1064.419	1596.125	3191.242	0.011	H5N4F1G1	1064.413-1064.425	10541.4	0.32
													total	3319988.7	100

LC-MS analysis of *N*-glycans on IgGs obtained from *Fut8*$^{+/-}$ mice treated with 1.2 mg/g/day L-fucose on the 7th day (C-7).

Supplemental Table 9 C-14: treatment with 1.2 mg/g/day L-fucose on the 14th day MS raw data: 21091610

No.		peptide	Retention time (min)	observed mass			caluculatted mass	throretical mass			△mass	Structure	mass range for EIC (± 6ppm)	Intensity	relative
				M+4H	M+3H	M+2H	M+H	M+3H	M+2H	M+H					
1		EEQFNSTFR	36.06			1118.967	2236.926	746.313	1118.966	2236.924	0.002	H2N3F1	1118.959-1118.973	5838.8	0.18
2			36.02			1199.994	2398.980	800.331	1199.992	2398.977	0.003	H3N3F1	1199.985-1199.999	29476.9	0.91
			36.18		800.331		2398.977				0.000		800.326-800.336	4034.2	0.12
3			35.87			1281.019	2561.030	854.349	1281.019	2561.030	0.000	H4N3F1	1281.011-1281.027	7543.8	0.23
4			36.09			1301.533	2602.058	868.024	1301.532	2602.056	0.002	H3N4F1	1301.524-1301.540	409060	12.56
			36.02		868.024		2602.056				0.000		868.019-868.029	429753	13.20
5			35.82			1382.561	2764.114	922.042	1382.558	2764.109	0.005	H4N4F1	1382.550-1382.566	163247	5.01
			35.82		922.042		2764.110				0.001		922.036-922.048	229969	7.06
6			36.11			1403.074	2805.140	935.717	1403.071	2805.135	0.005	H3N5F1	1403.064-1403.080	5719.9	0.18
			36.09		935.718		2805.138				0.003		935.711-935.723	10239.2	0.31
7			37.04		956.711		2868.117	956.712	1434.564	2868.120	-0.003	H4N3F1G1	956.706-956.718	2284.2	0.07
8			35.72			1463.587	2926.166	976.059	1463.585	2926.162	0.004	H5N4F1	1463.576-1463.594	24090.8	0.74
			35.75		976.060		2926.164				0.002		976.053-976.065	46049.6	1.41
9			35.82		989.733		2967.183	989.735	1484.098	2967.188	-0.005	H4N5F1	989.729-989.741	2670.8	0.08
10			36.92		1010.732		3030.180	1010.730	1515.590	3030.173	0.007	H5N3F1G1	1010.723-1010.735	2010.5	0.06
11			36.95			1536.106	3071.204	1024.405	1536.103	3071.199	0.005	H4N4F1G1	1536.095-1536.113	56405.5	1.73
			36.95		1024.406		3071.202				0.003		1024.399-1024.411	145564	4.47
12			36.67			1617.134	3233.260	1078.423	1617.130	3233.252	0.008	H5N4F1G1	1617.120-1617.140	21574.1	0.66
			36.73		1078.424		3233.256				0.004		1078.417-1078.429	85490.9	2.63
13			37.50			1770.675	3540.342	1180.786	1770.675	3540.342	0.000	H5N4F1G2	1770.664-1770.686	6115.7	0.19
			37.50		1180.788		3540.348				0.006		1180.779-1180.793	42236.3	1.30
14			36.41			1228.503	2455.998	819.338	1228.503	2455.998	0.000	H3N4	1228.496-1228.510	5826.8	0.18
15			36.41		819.340		2456.004				0.006		819.333-819.343	9603.1	0.29
16			36.14			1309.531	2618.054	873.356	1309.529	2618.051	0.003	H4N4	1309.521-1309.537	2930.2	0.09
17			36.26		873.354		2618.046				-0.005		873.351-873.361	2921.4	0.09
18			35.75			1390.558	2780.108	927.373	1390.556	2780.104	0.004	H5N4	1390.548-1390.564	1311.5	0.04
19		EDYNSTLR	30.50			1119.962	2238.916	746.976	1119.960	2238.913	0.003	H3N3F1	1119.954-1119.968	5970.2	0.18
20			30.33			1200.987	2400.966	800.994	1200.987	2400.966	0.000	H4N3F1	1200.980-1200.994	3131.9	0.10
21	a		29.87			1221.499	2441.990	814.670	1221.500	2441.993	-0.003	H3N4F1	1221.493-1221.507	25648.5	0.79
	b		30.36			1221.502	2441.996				0.003			101468	3.12
	a		29.81		814.669		2441.991				-0.002		814.664-814.674	21406.9	0.66
	b		30.36		814.670		2441.994				0.001			78311.6	2.40
22	a		29.55			1302.528	2604.048	868.687	1302.526	2604.045	0.003	H4N4F1	1302.519-1302.535	176524	5.42
	b		30.15			1302.528	2604.048				0.003			145738	4.48
	a		29.52		868.687		2604.045				0.000		868.682-868.692	142518	4.38
	b		30.12		868.688		2604.048				0.003			131016	4.02
23	a		29.52			1383.555	2766.102	922.705	1383.553	2766.098	0.004	H5N4F1	1383.545-1383.561	43454.2	1.33
	b		30.12			1383.555	2766.102				0.004			30779.2	0.95
	a		29.52		922.705		2766.099				0.001		922.699-922.711	52068.3	1.60
	b		30.12		922.705		2766.099				0.001			30911.9	0.95
24	a		30.24			1456.074	2911.140	971.051	1456.072	2911.136	0.004	H4N4F1G1	1456.063-1456.081	22809.1	0.70
	b		30.80			1456.074	2911.140				0.004			12571.3	0.39
	a		30.21		971.052		2911.140				0.004		971.044-971.056	39897.7	1.23
	b		30.88		971.052		2911.140				0.004			25099.8	0.77
25			29.46		976.722		2928.150	976.722	1464.579	2928.151	-0.001	H6N4F1	976.76-976.728	2095.1	0.06
26	a		30.15			1537.100	3073.192	1025.068	1537.098	3073.188	0.004	H5N4F1G1	1537.089-1537.107	26193.7	0.80
	b		30.65			1537.102	3073.196				0.008			16940.4	0.52
	a		30.12		1025.069		3073.191				0.003		1025.062-1025.074	72971.7	2.24
	b		30.65		1025.069		3073.191				0.003			43026.5	1.32
27			30.47		1079.091		3235.257	1079.086	1618.124	3235.241	0.016	H6N4F1G1	1079.080-1079.092	1899	0.06
28	a		30.65			1690.644	3380.280	1127.432	1690.643	3380.279	0.001	H5N4F1G2	1690.633-1690.653	5756.6	0.18
	b		31.22			1690.648	3380.288				0.009			1518.5	0.05
	a		30.59		1127.433		3380.283				0.004		1127.424-1127.438	17690.6	0.54
	b		31.19		1127.433		3380.283				0.004			10756.7	0.33
29			30.27			1383.046	2765.084	922.365	1383.043	2765.078	0.006	H4N4G1	1383.038-1383.054	1059.4	0.03
30		EAQYNSTFR	31.65			1178.991	2356.974	786.327	1178.987	2356.966	0.008	H3N3F1	1178.980-1178.994	4478.8	0.14
			31.65		786.325		2356.959				-0.007		786.322-786.332	1150	0.04
31			31.44			1260.012	2519.016	840.345	1260.013	2519.019	-0.003	H4N3F1	1260.005-1260.021	2657.9	0.08
32			31.62			1280.528	2560.048	854.021	1280.527	2560.046	0.002	H3N4F1	1280.519-1280.535	50023	1.54
			31.62		854.021		2560.047				0.001		854.015-854.025	47557.9	1.46
33			31.40			1361.555	2722.102	908.038	1361.553	2722.098	0.004	H4N4F1	1361.545-1361.561	30395.7	0.93
			31.40		908.039		2722.101				0.003		908.033-908.043	38510.2	1.18
34			31.28			1442.582	2884.156	962.056	1442.579	2884.151	0.005	H5N4F1	1442.571-1442.589	7320.9	0.22
35			32.39			1515.104	3029.200	1010.402	1515.098	3029.189	0.011	H4N4F1G1	1515.089-1515.107	2768.8	0.09
			32.33		1010.402		3029.190				0.001		1010.395-1010.407	10150.6	0.31
36			32.12			1596.130	3191.252	1064.419	1596.125	3191.242	0.010	H5N4F1G1	1596.115-1596.135	2112	0.06
			32.21		1064.421		3191.247				0.005		1064.413-1064.425	12993.3	0.40
37			32.82		1166.784		3498.336	1166.783	1749.670	3498.332	0.004	H5N4F1G2	1166.775-1166.789	5042.4	0.15
													total	3256361	100

LC-MS analysis of *N*-glycans on IgGs obtained from *Fut8*+/− mice treated with 1.2 mg/g/day L-fucose on the 14th day (C-14).

Supplemental Table10

Relative levels of fucosylated and none fucosylated *N*-glycans on IgGs obtained from Fut8$^{+/-}$ mice treated with or without L-fucose at indicated doses

	Relative Abundance[%]		
	A-0	A-7	A-14
fucosylated glycan	98.30	99.42	99.33
none fucosylated glycan	1.70	0.58	0.67
total	100.00	100.00	100.00
	A: 0.15 mg/g/day; 0, 7, or 14 days		

	Relative Abundance[%]		
	B-0	B-7	B-14
fucosylated glycan	98.94	99.25	99.33
none fucosylated glycan	1.06	0.75	0.67
total	100.00	100.00	100.00
	B: 0.4 mg/g/day; 0, 7, or 14 days		

	Relative Abundance[%]		
	C-0	C-7	C-14
fucosylated glycan	99.51	99.41	99.31
none fucosylated glycan	0.49	0.59	0.69
total	100.00	100.00	100.00
	C: 1.2 mg/g/day; 0, 7, or 14 days		

Supplemental Table11

A comparison of IgG subclass and Igκ levels in Fut8$^{+/-}$ mice treated with or without L-fucose at indicated doses

WT and Hetero	Intensity	
	WT	Hetero
MS raw data	21080404	21080405
IgG1	518000	119000
IgG2	3350000	598000
IgG3	314000	211000
IgK	4880000	564000

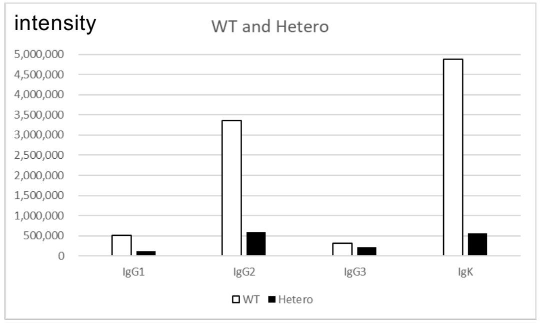

Hetero Fut8$^{+/-}$	Intensity		
A: 0.15 mg/g/day	0 day	7 day	14 day
MS raw data	21091602	21091605	21091608
IgG1	221000	417000	859000
IgG2	943000	2520000	4740000
IgG3	254000	1140000	2310000
IgK	792000	2410000	4530000

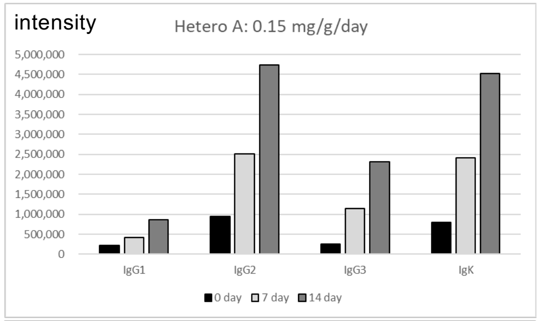

Hetero Fut8$^{+/-}$	Intensity		
B: 0.4 mg/g/day	0 day	7 day	14 day
MS raw data	21091603	21091606	21091609
IgG1	204000	386000	553000
IgG2	1310000	3390000	4740000
IgG3	824000	2500000	3100000
IgK	1410000	3280000	4700000

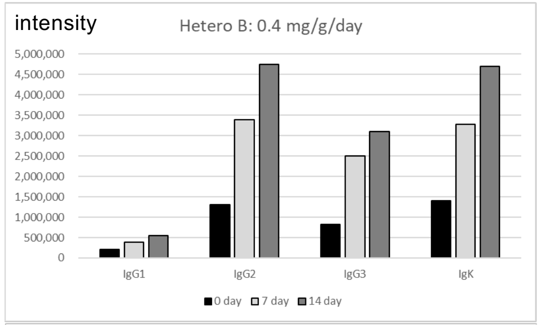

Hetero Fut8$^{+/-}$	Intensity		
C: 1.2 mg/g/day	0 day	7 day	14 day
MS raw data	21091604	21091607	21091610
IgG1	358000	759000	788000
IgG2	736000	3140000	2430000
IgG3	233000	909000	642000
IgK	1260000	3550000	3650000

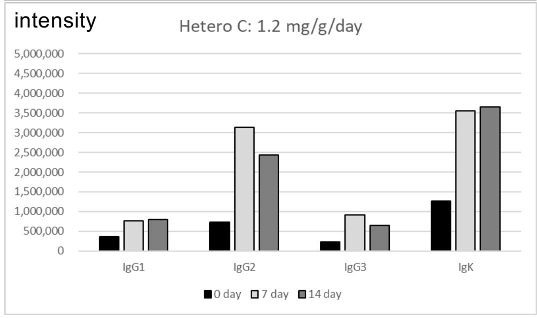

Supplement: Supplementary Tables [file mmc2.pdf]
